# Systematic identification and characterization of regulatory elements derived from human endogenous retroviruses

**DOI:** 10.1371/journal.pgen.1006883

**Published:** 2017-07-12

**Authors:** Jumpei Ito, Ryota Sugimoto, Hirofumi Nakaoka, Shiro Yamada, Tetsuaki Kimura, Takahide Hayano, Ituro Inoue

**Affiliations:** 1 Division of Human Genetics, Department of Integrated Genetics, National Institute of Genetics, 1111 Yata, Mishima, Shizuoka, Japan; 2 Department of Genetics, School of Life Science, SOKENDAI (The Graduate University for Advanced Studies), 1111 Yata, Mishima, Shizuoka, Japan; 3 Department of Pediatrics, Tokai University School of Medicine, 143 Shimokasuya, Isehara, Kanagawa, Japan; University of Utah School of Medicine, UNITED STATES

## Abstract

Human endogenous retroviruses (HERVs) and other long terminal repeat (LTR)-type retrotransposons (HERV/LTRs) have regulatory elements that possibly influence the transcription of host genes. We systematically identified and characterized these regulatory elements based on publicly available datasets of ChIP-Seq of 97 transcription factors (TFs) provided by ENCODE and Roadmap Epigenomics projects. We determined transcription factor-binding sites (TFBSs) using the ChIP-Seq datasets and identified TFBSs observed on HERV/LTR sequences (HERV-TFBSs). Overall, 794,972 HERV-TFBSs were identified. Subsequently, we identified “HERV/LTR-shared regulatory element (HSRE),” defined as a TF-binding motif in HERV-TFBSs, shared within a substantial fraction of a HERV/LTR type. HSREs could be an indication that the regulatory elements of HERV/LTRs are present before their insertions. We identified 2,201 HSREs, comprising specific associations of 354 HERV/LTRs and 84 TFs. Clustering analysis showed that HERV/LTRs can be grouped according to the TF binding patterns; HERV/LTR groups bounded to pluripotent TFs (e.g., SOX2, POU5F1, and NANOG), embryonic endoderm/mesendoderm TFs (e.g., GATA4/6, SOX17, and FOXA1/2), hematopoietic TFs (e.g., SPI1 (PU1), GATA1/2, and TAL1), and CTCF were identified. Regulatory elements of HERV/LTRs tended to locate nearby and/or interact three-dimensionally with the genes involved in immune responses, indicating that the regulatory elements play an important role in controlling the immune regulatory network. Further, we demonstrated subgroup-specific TF binding within LTR7, LTR5B, and LTR5_Hs, indicating that gains or losses of the regulatory elements occurred during genomic invasions of the HERV/LTRs. Finally, we constructed dbHERV-REs, an interactive database of HERV/LTR regulatory elements (http://herv-tfbs.com/). This study provides fundamental information in understanding the impact of HERV/LTRs on host transcription, and offers insights into the transcriptional modulation systems of HERV/LTRs and ancestral HERVs.

## Author summary

Human endogenous retroviruses (HERVs) are genomic “fossils” of ancient exogenous retroviruses and their descendants that were replicated in host germ cells. The traits and evolutionary dynamics of ancient retroviruses and their descendants can be inferred by scrutinizing present-day HERVs. We systematically identified regulatory elements of HERVs based on publicly available datasets of ChIP-Seq of 97 TFs. Clustering analysis showed that HERV/LTRs can be grouped by the TF-binding patterns; HERV/LTR groups bounded by pluripotent TFs (e.g., SOX2, POU5F1, and NANOG), embryonic endoderm/mesendoderm TFs (e.g., GATA4/6, SOX17, and FOXA1/2), and hematopoietic TFs (e.g., SPI1 (PU1), GATA1/2, and TAL1) were identified. By analyzing the three-dimensional chromosomal interactions, we demonstrated that regulatory elements of HERVs tend to interact with host immune-response genes. We further demonstrated heterogeneities of regulatory elements within LTR7; SOX2, POU5F1, and KLF4-binding sites were highly enriched in the youngest subgroup of LTR7, which had the highest transcriptional activity in pluripotent cells. This suggests that the subgroup acquired those regulatory activities for efficient replication in the host germ cells. Finally, we constructed dbHERV-REs, an interactive database of HERV/LTR regulatory elements (http://herv-tfbs.com/). This study provides insights into regulatory elements of HERVs and transcriptional modulations of host genes by HERVs.

## Introduction

Transposable elements (TEs) are mobile genomic DNA sequences that occupy approximately half of the human genome and are capable of autonomous or non-autonomous replication [1]. TEs were initially thought to be parasitic, selfish, and junk DNA [2]. Decades of research accumulated evidences that some TEs are co-opted by the host and acquire new physiological functions as protein-coding/-non-coding genes and regulatory elements for host genes [3–15]. TEs have their own regulatory elements for transcription and replication [9–24]. Such TE-derived regulatory elements are abundant in the human genome and have various effects on transcriptional modulations of host genes as promoters, enhancers, and insulators [9–15, 25–34]. Notably, numerous TE insertions sharing the same regulatory elements can affect multiple genes in a coordinate manner. Several studies have suggested that TE insertions have contributed to the rewiring and evolution of regulatory networks by recruiting multiple genes into the same regulatory circuit [10–15, 33–37].

Human endogenous retroviruses (HERVs) and other long terminal repeat (LTR)-type retrotransposons (HERV/LTRs) are a class of TEs that developed through the infection of host germ cells by ancient retroviruses, followed by their transmission to the offspring (referred to as endogenization) [38]. HERV/LTRs (and other retroviruses) are composed of 5′- and 3′-LTR sequences, which modulate viral transcription and internal sequences containing viral genes [38]. In the host chromosome, HERV/LTRs are present either as a complete structure (referred to as provirus) or as a single LTR structure (referred to as solo LTR) [38]. HERV/LTRs occupy approximately 8% of the human genome [1]. HERVs have lost their replication and transposition activities in germ cells owing to the accumulation of mutations [38]. According to RepeatMasker (20-Mar-2009) (http://www.repeatmasker.org/), 375 and 130 types of LTRs and internal sequences of HERV/LTRs, respectively, have been discovered in the human genome. This indicates that HERV/LTRs show the greatest diversity for all classes of human TEs.

HERV/LTRs are transcribed by the host machinery, including RNA polymerase II (Pol II), and many regulatory elements bounded to Pol II-associated transcription factors (TFs) are present in LTR sequences [38]. HERV/LTRs show the highest enrichment in regulatory sequences such as open chromatin regions among all classes of human TEs [9, 37]. Reflecting the considerable diversity of HERV/LTRs, each type of HERV/LTRs has various regulatory elements involved in regulating diverse host genes [9–15, 20–24]. For instance, LTR7 insertions provide POU5F1- (OCT4-), SOX2-, KLF4-, and NANOG-binding sites for protein-coding/non-coding genes, which are essential for maintaining pluripotency in embryonic stem (ES) and induced pluripotent stem (iPS) cells [10–13, 39]. As a further example, MER41 insertions harboring STAT1- and IRF1-binding sites in several genes contribute to primate-specific interferon responses [14]. Clarifying the properties of HERV/LTRs regulatory elements provides a better understanding of their impact on host transcriptional regulation.

We systematically identified and characterized regulatory elements derived from HERV/LTRs based on publicly available datasets of chromatin immunoprecipitation followed by sequencing (ChIP-Seq) of sequence-specific TFs. The ChIP-Seq datasets were provided by ENCODE [40] and Roadmap Epigenomics (Roadmap) (Tsankov *et al*. [41]) projects. Previous studies have comprehensively investigated regulatory elements of TEs (including HERV/LTRs) based on the ENCODE dataset [9, 37, 40]. Jacques *et al*. demonstrated that the majority of primate-specific regulatory sequences are derived from TEs [9]. Because this particular study was mainly focused on the dataset of DNase I hypersensitive sites (DHSs), it provided limited insight into the specific associations of TEs and TFs [9]. Sundaram *et al*. showed specific associations of TEs and TFs using a dataset of ChIP-Seq for TFs [37]. However, the number of sequence-specific TFs investigated in that study was restricted (15 sequence-specific TFs) owing to the focus on TFs for which ChIP-Seq was performed in both human and mouse cells to compare the binding profiles [37]. In the present study, we performed a more comprehensive study than earlier of regulatory elements on HERV/LTRs by evaluating 519 ChIP-Seq datasets of 97 sequence-specific TFs (S1 and S8 Tables). Furthermore, we constructed dbHERV-REs, a database of HERV/LTR regulatory elements with an interactive interface (http://herv-tfbs.com/). This study provides fundamental information to understand the impact of HERV/LTRs on host transcription.

## Results

### Detection of transcription factor-binding sites (TFBSs) using ChIP-Seq datasets

We analyzed 519 ChIP-Seq datasets provided by ENCODE and Roadmap (S8 Table). The datasets included ChIP-Seq analysis of 97 sequence-specific and Pol II-associated TFs (S1 Table). The ChIP-Seq experiments were performed using 94 cell types. Although ENCODE and Roadmap provided datasets of pre-determined ChIP-Seq peaks (pre-determined TFBSs), there are substantial differences in analytical pipelines between the two projects (S2 Table). Therefore, we determined ChIP-Seq peaks using a uniform analytical pipeline (S1B Fig). When focusing on repetitive elements such as HERV/LTRs, it is important to check whether multiple mapped reads (reads can be mapped to multiple genomic regions) are excluded in data analysis of next generation sequencing [37, 42]. If multiple mapped reads are not excluded, false positive peaks may be detected at regions that have sequences similar to those authentically bounded by the TF. If they are excluded, it is unfeasible to identify ChIP-Seq peaks on recently integrated HERV/LTRs that show low sequence divergence among the copies. Some studies on TEs excluded multiple mapped reads [9], while others did not [10]. Therefore, we generated two types of ChIP-Seq peak datasets: all-read and unique-read TFBSs (S1B Fig). All-read TFBSs are ChIP-Seq peaks that were determined with all reads mapped to the human reference genome. The unique-read TFBSs are ChIP-Seq peaks that were determined with only the reads uniquely mapped to the reference genome; in other words, multiple mapped reads were excluded before the peak calling of ChIP-Seq. Consequently, we identified 7,262,985 and 6,833,767 of all- and unique-read TFBSs, respectively (S2A Fig); for estimating the numbers, overlapped TFBSs of the same TF were merged among cell types. Detailed information on ChIP-Seq is summarized in S8 and S9 Tables.

### Detection of TFBSs on HERV/LTRs (HERV-TFBSs)

**Fig 1:**
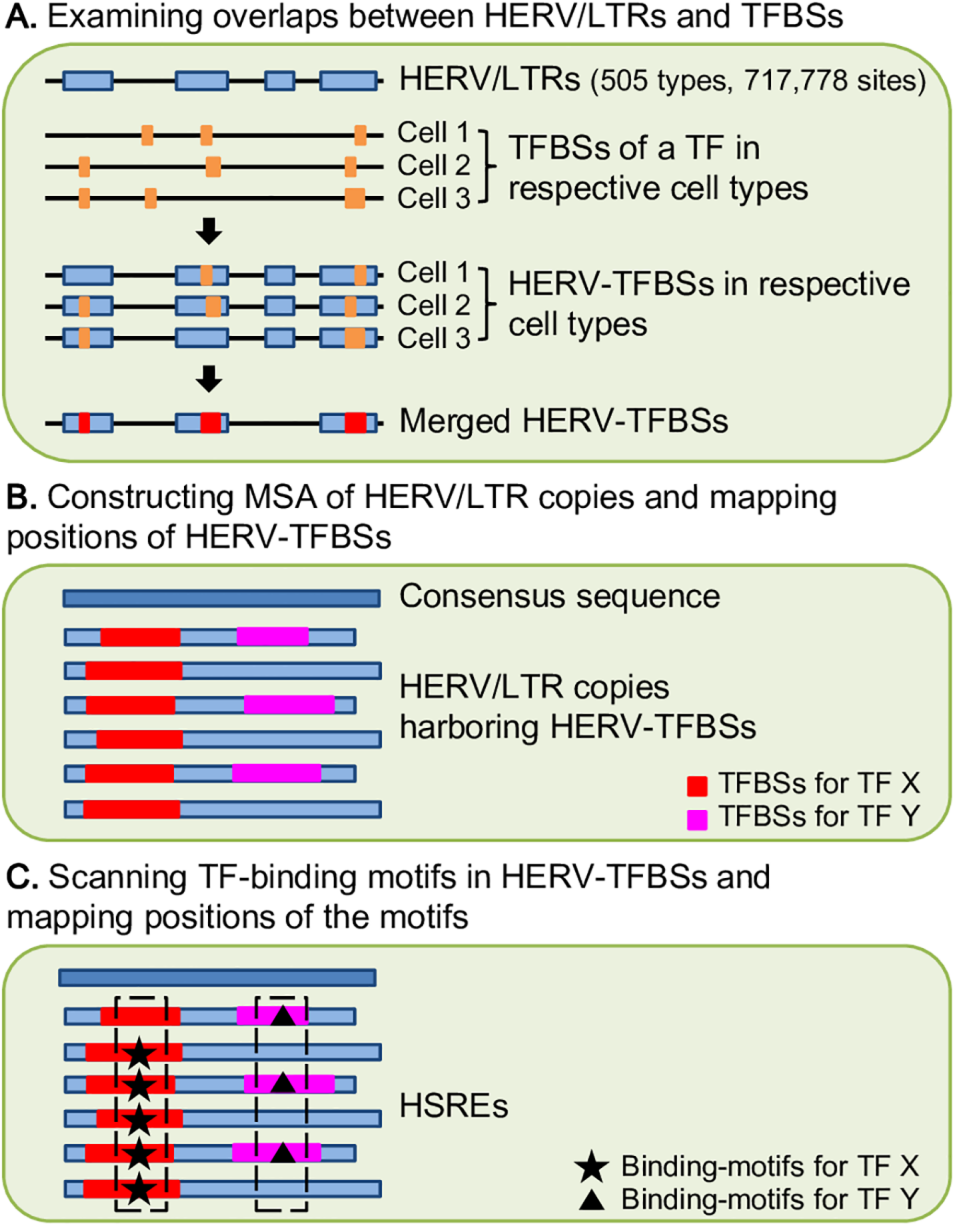
Scheme of identification of HERV-TFBSs and HSREs. HERV-TFBSs and HSREs were identified separately using ENCODE and Roadmap datasets. HERV-TFBSs and HSREs were identified for all- and unique-read TFBSs. A) HERV-TFBSs were identified in respective cell types by examining overlaps between HERV/LTRs and TFBSs. HERV-TFBSs of each TF were merged among cell types (merged HERV-TFBSs). B) In each HERV/LTR type, MSA of HERV/LTR copies was constructed with the consensus sequence, and then the position of the merged HERV-TFBS was mapped on each HERV/LTR sequence in the MSA. Red and pink regions indicate HERV-TFBSs for TF X and Y, respectively. C) TF-binding motif was scanned in HERV-TFBS and mapped on each HERV/LTR sequence in the MSA. Star and triangle marks indicate TF-binding motifs for TF X and Y, respectively. A set of TF-binding motifs was regarded as HSRE if the TF-binding motifs were shared among greater than 60% of HERV-TFBSs at the same position in MSA. Boxed TF-binding motifs are HSREs for TF X and Y, respectively.

We identified TFBSs observed on HERV/LTR sequences (HERV-TFBS overlaps (HERV-TFBSs)) belonging to the all- and unique-read TFBSs (Fig 1A). We first identified HERV-TFBSs in each cell type, and then merged HERV-TFBSs of the same TF in all cell types (merged HERV-TFBSs). Thus, we identified 866,649 merged HERV-TFBSs from all-read TFBSs and 794,972 from unique-read TFBSs (S2A Fig). HERV-TFBSs respectively occupied 11.9% and 11.6% of entire TFBSs in all- and unique-read TFBSs (S2A Fig).

To evaluate the differences between all- and unique-read TFBSs, we compared the number of HERV-TFBSs for both the TFBS datasets. In most HERV/LTR types, the numbers of HERV-TFBSs were approximately the same for all- and unique-read TFBSs; however, the difference was quite large for some HERV/LTR types such as LTR7 and LTR5_Hs (S3A, S3C and S3D Fig). These HERV/LTR types were recently inserted [see dbHERV-REs (http://herv-tfbs.com)] and showed low ‘genomic mappability’ (sequence uniqueness) (S3B and S3E Fig). Therefore, a substantial number of sequence reads was not uniquely mapped on the HERV/LTRs and was discarded. Based on these results, we generally used unique-read TFBSs for further analyses. When we individually focused on HERV/LTR types with low genomic mappability, such as LTR7 and LTR5_Hs, we used all-read TFBSs.

We compared HERV/LTRs with other classes of TEs with respect to the TF binding profiles. In the unique-read TFBSs, LINE, SINE, and DNA transposons were respectively overlapped to 15%, 16%, and 6% of the entire TFBSs (S4A Fig). It is important to check whether a TF binds to a type of TE significantly more than expected, because TEs occupy a large fraction of the genome, and therefore, TF binding would be partially observed on the TEs regardless of the absence of a special association between the TEs and TFs. Therefore, we evaluated statistical enrichment of binding of a TF in respective types of TEs to random expectation. The enrichment of TF binding was measured using a randomization test shuffling genomic positions of TFBSs (see Materials and Methods). Subsequently, we counted the number of TFs bounded significantly to a type of TE, and then the distribution was compared among the TE classes (S4B Fig). We demonstrated that the number of TFs binding significantly to a TE type tended to be substantially higher in the HERV/LTR class than the other TE classes (S4B Fig). In the other TE classes, a few TEs were bounded by a large number of TFs (S4C Fig). Thus, HERV/LTRs were distinguished from the other TEs with respect to numbers of TF bindings. Previous studies reported the same tendency that HERV/LTRs have more regulatory sequences (e.g., DHSs and TFBSs) than the other TEs [9, 37].

### Classification of HERV/LTRs based on TF binding patterns

**Fig 2:**
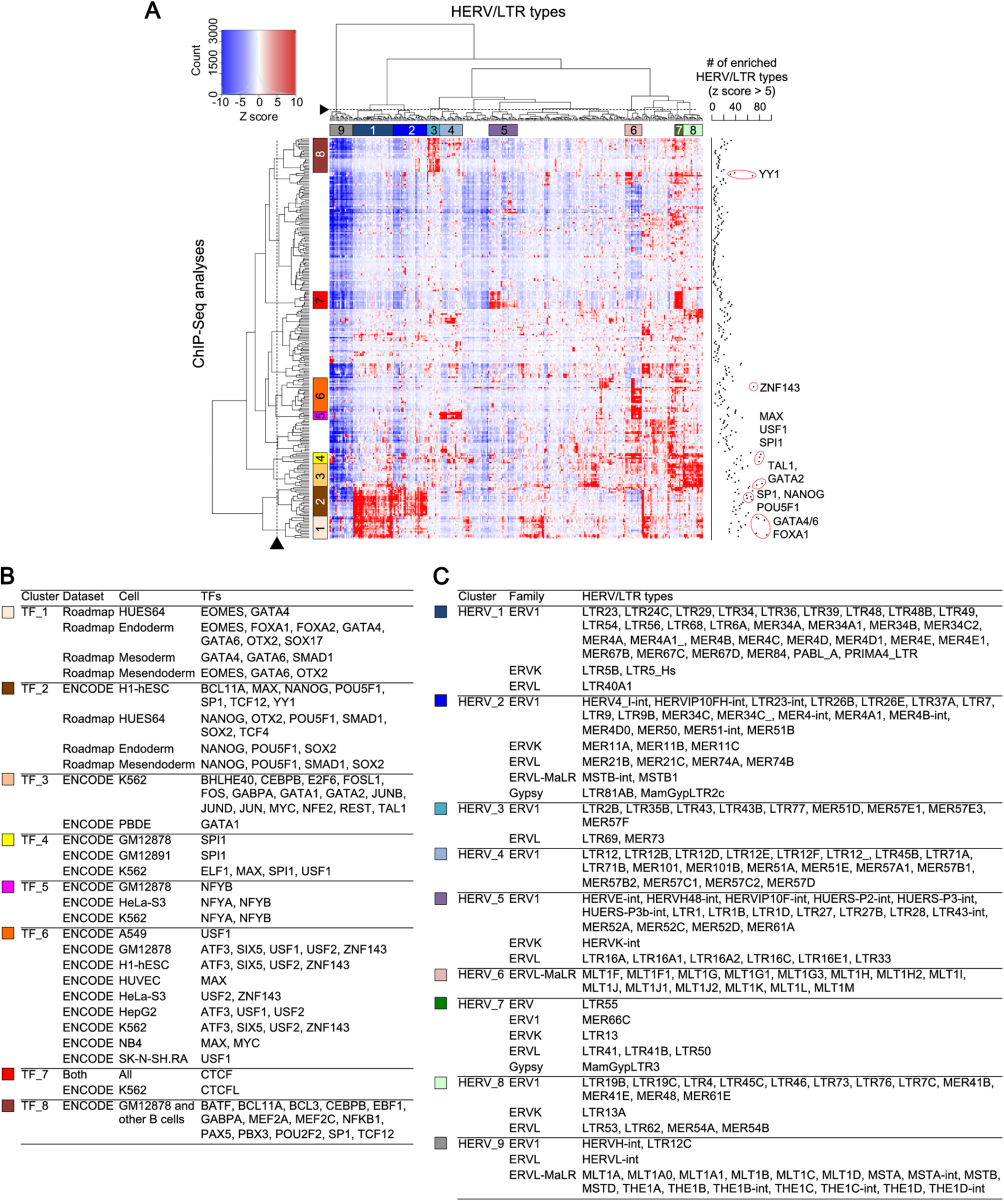
Statistical enrichment of respective TFBSs in each type of HERV/LTRs. Results from unique-read TFBSs are shown. A) The heatmap with hierarchical clustering, which shows statistical enrichment of respective TFBSs in each type of HERV/LTRs. Color in heatmap (from blue to red) indicates enrichment significance (z score) to random expectation. The row indicates TFBSs from a ChIP-Seq analysis. The column indicates a HERV/LTR type. The dendrograms were cut at heights denoted by broken lines. Fourteen clusters were identified for HERV/LTRs and TFBSs. Of these, characteristic clusters of TFBSs (TF_1–8) and HERV/LTRs (HERV_1–9) are shown. The cut heights and the characteristic clusters were manually chosen according to dendrograms and color patterns in heatmap. The number of HERV/LTR types highly enriched in each TFBS dataset (z score >5) is shown on the right side of the heatmap. B) Characteristic clusters of TFBSs (TF_1–8). Ectoderm, endoderm, mesoderm, and mesendoderm were differentiated from HUES64 cells. C) Characteristic clusters of HERV/LTRs (HERV_1–9). Classification of the HERV/LTR family is based on RepeatMasker (20-Mar-2009) (http://www.repeatmasker.org/).

To understand the characteristic patterns of TF binding to HERV/LTRs, we performed hierarchical clustering analysis based on statistical enrichments of TF binding to random expectation (Fig 2A). Enrichment significance was measured for each combination between HERV/LTRs and TFBSs in respective cell types to consider the cell type-specific binding of TFs to HERV/LTRs. Fourteen HERV/LTR and TFBS clusters were identified (Fig 2A), of which, we characterized 8 TFBS clusters (TF_1–8) (Fig 2B) [40, 41, 43–45]: TF_1 contained TFBSs for FOXA1/2, GATA4/6, and SOX17, which are critical for the differentiation of embryonic mesendoderm or endoderm. TF_2 contained TFBSs for POU5F1, SOX2, and NANOG, essential for pluripotency of ES and iPS cells. TF_3 contained TFBSs for GATA1/2 and TAL1, essential in hematopoietic and leukemia cells. TF_4 contained SPI1, which is critical for the differentiation of hematopoietic cells. TF_5 and TF_6 contained TFBSs for NFYA/B, USF1/2, and other TFs expressed in a broad-range of cell types. TF_8 contained TFBSs for PAX5 and PBX3, essential for the differentiation of B lymphocytes. TF_7 contained CTCF-binding sites found in all the cell types, which function as insulators and regulate chromatin architecture. We also characterized 9 HERV/LTR clusters (HERV_1–9) (Fig 2A–2C). HERV_1 was enriched in TF_1 (endoderm TF cluster) and TF_2 (pluripotent TF cluster). HERV_2 was enriched in TF_2 (pluripotent TF cluster). HERV_3 was enriched in TF_8 (B-lymphocyte TF cluster). HERV_4 cluster was enriched in TF_5 cluster. HERV_5 and HERV_7 were enriched in TF_7 (CTCF cluster). HERV_6 was enriched in TF_5 and TF_6 clusters. HERV_8 was enriched in TF_3 and TF_4 (hematopoietic TF clusters). Lastly, HERV_9 was not enriched in most TFBSs. Taken together, we identified the characteristic clusters of HERV/LTRs by the hierarchical clustering analysis, indicating that HERV/LTR types can be classified based on their TFBSs. Each HERV/LTR cluster typically contained several HERV/LTR types belonging to different HERV/LTR families (Fig 2C). This indicates that the pattern of HERV/LTR regulatory elements do not match their phylogenic classifications. TFBSs for FOXA1/2, GATA4/6, NANOG, POU5F1, SP1, GATA2, TAL1, MAX, USF1, SPI1, ZNF143, and YY1 were enriched in various types of HERV/LTRs (Fig 2A right).

### Identification of HERV/LTR-shared regulatory elements (HSREs)

HERV/LTR-shared regulatory element (HSRE) was defined as a TF-binding motif identified in a substantial fraction of HERV-TFBSs at the same consensus position (Fig 1). HSREs can indicate that the regulatory elements of HERV/LTRs are present before their insertions into the respective genomic loci [46]. We identified HSREs according to a scheme shown in Fig 1. HSREs were identified separately from ENCODE and Roadmap dataset. In total, 2,525 and 2,201 types of HSREs were respectively identified from all- and unique-read TFBSs. Regarding all-read TFBSs, HSREs comprised specific associations of 370 HERV/LTRs and 85 TFs. These HSREs were composed of 255,225 genomic loci and present in 21% of the total HERV-TFBSs and in 2.5% of the entire TFBSs (S2A Fig). For unique-read TFBSs, HSREs comprised specific associations between 354 HERV/LTRs and 84 TFs. These HSREs were composed of 178,121 genomic loci and present in 17% of the total HERV-TFBSs and in 2.0% of the entire TFBSs (S2A Fig). In most HERV/LTR types, the numbers of identified HSREs were approximately the same between unique- and all-read TFBSs; however, in HERV/LTR types with low genomic mappability (e.g., LTR7 and LTR5_Hs), more HSREs were identified from all-read TFBSs than unique-read TFBSs (S2B and S2C Fig). This was consistent with the comparison of the number of HERV-TFBSs between the two datasets (S3 Fig). Concerning HERV-TFBSs harboring HSREs, approximately half of HERV-TFBSs had more than one of TF-binding motif corresponding to HSRE (S5A Fig). Most of the HSREs were identified in LTR sequences (87%; 1,935/2,201 combinations in unique-read TFBSs), and the others were identified in the internal sequences of HERV/LTRs (13%; 266/2,201 combinations). Large proportions of copies of LTR12, LTR22, LTR13 groups and LTR6B contained HSREs (with respect to proportions of copies harboring HSREs, top 15 of HERV/LTRs are shown in Table 1). Regarding TFs, MER41B, LTR13/13A, LTR8/8A, LTR10A/10F, LTR9/9B, and LTR5B/5_Hs contained various HSREs (S5B Fig). HSREs were identified in both recently and anciently inserted HERV/LTRs, the latter of which was inserted into the genome of the common ancestor of the clade *Eutheria* (S5C, S5D and S5E Fig). As degrees of divergences (or ‘ages’) of HERV/LTRs increased, proportions of copies harboring HSREs decreased (S5D Fig), indicating regulatory elements of ancient HERV/LTRs were more divergent than those of young HERV/LTRs. As in the case of HERV-TFBSs, HSREs bounded by TFs essential for pluripotent, embryonic endoderm, and hematopoietic cells were frequently identified in addition to CTCF (S5F and S5G Fig). HSREs bounded by CTCF were frequently observed in internal sequences rather than LTR sequences (S5H and S5I Fig). Regarding LTR2B, LTR5B, MER41B, and MLT1J, HSREs identified from unique-read TFBSs are shown in S6 Fig.

**Table 1:** Absolute numbers and proportions of HERV/LTR copies harboring HSREs.

Family	Type	# of copies with HSREs	Proportion
ERV1	LTR12	675	0.87
ERV1	LTR6B	123	0.80
ERVK	LTR22C	294	0.75
ERV1	LTR12_	414	0.75
ERV1	LTR12C	1,993	0.73
ERVK	LTR13	355	0.72
ERVK	LTR13A	130	0.69
ERV1	LTR12D	336	0.69
ERVK	LTR22B	157	0.67
ERV1	MER48	129	0.67
ERVL	LTR18A	170	0.66
ERVK	LTR22A	115	0.61
ERV1	LTR10F	259	0.58
ERV1	LTR10A	181	0.58
ERV1	LTR12B	119	0.56

### Characteristics of HSREs in LTR7

**Fig 3:**
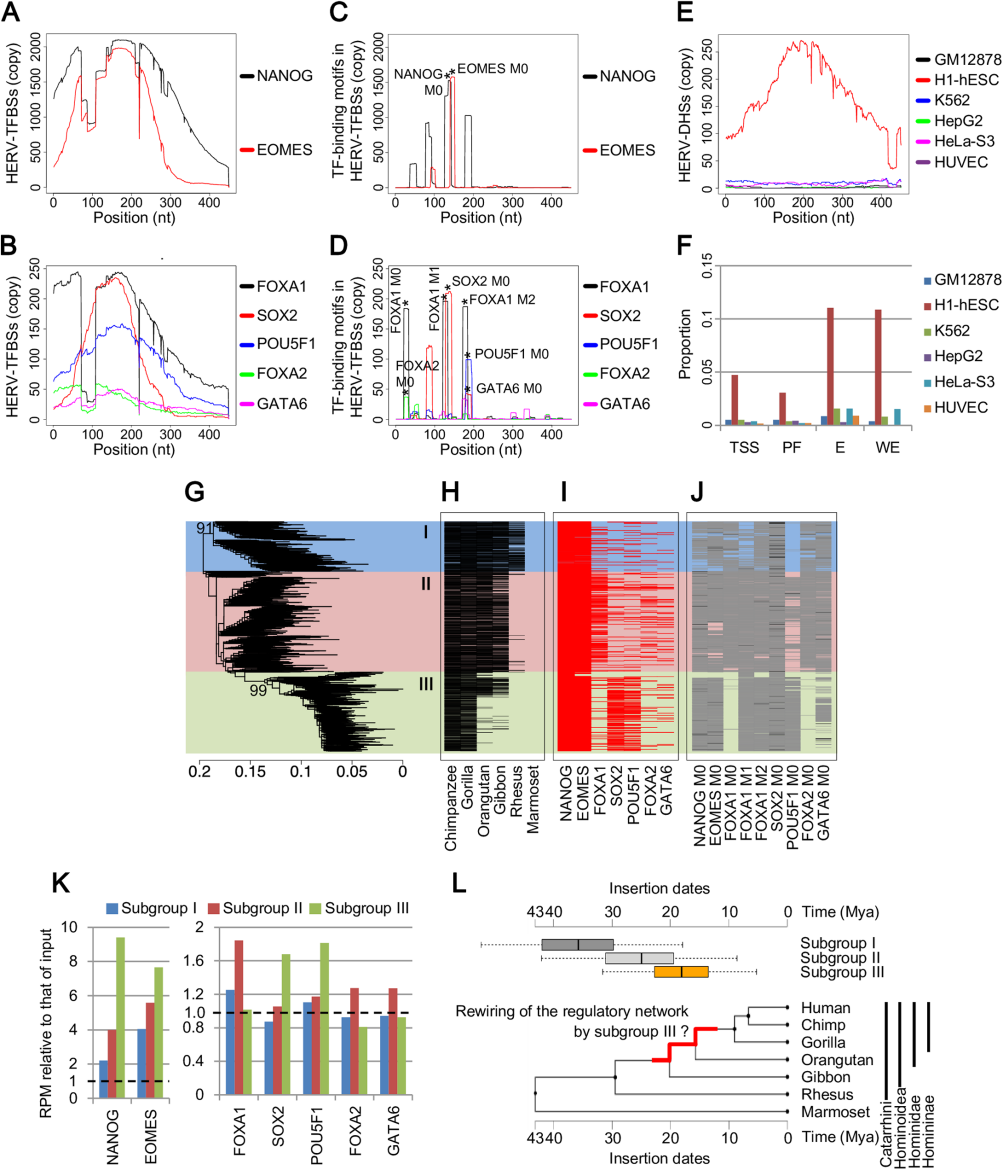
Characteristics of HSREs identified in LTR7 from the Roadmap dataset. Results from all-read TFBSs are shown. A) and B) Number of HERV-TFBSs mapped on each consensus position of LTR7. Results for NANOG and EOMES are shown in (A), and those for FOXA1, SOX2, POU5F1, FOXA2, and GATA6 are sown in (B). The X-axis indicates nucleotide position of the consensus sequence of LTR7. The Y-axis indicates the number of HERV/LTR copies harboring HERV-TFBSs at each position. C) and D) Number of TF-binding motifs in HERV-TFBSs mapped on each consensus position of LTR7. Results for NANOG and EOMES are shown in (C), and those for FOXA1, SOX2, POU5F1, FOXA2, and GATA6 are shown in (D). The X-axis indicates consensus position of LTR7. The Y-axis indicates number of HERV/LTR copies harboring the TF-binding motifs in TFBSs at each position. Peaks of the motifs corresponding to HSREs are denoted by an asterisk (*) with motif names (e.g., SOX2 M0). E) The number of HERV-DHSs (DHSs on HERV/LTRs) mapped on each consensus position of LTR7. The X-axis indicates consensus position of LTR7. The Y-axis indicates the number of HERV/LTR copies harboring HERV-DHSs at each position. F) Proportion of LTR7 copies overlapped with each chromatin state predicted by genome segmentation method [47–49]. TSS, promoter region including TSS; PF, predicted promoter flanking region; E, enhancer; WE, weak enhancer or open chromatin cis regulatory element. G) The unrooted phylogenetic tree of LTR7 copies reconstructed using the maximum likelihood method with RAxML [67]. Fragmented and outlier copies were excluded from the analysis. In total, 1,914 (out of 2,344) of LTR7 copies were included in the tree. Representative supporting values calculated by Shimodaira-Hasegawa (SH)-like test [68] are shown on the corresponding branches. Identified phylogenetic subgroups (subgroups I, II, and III) are shown. H) Orthologous copies of LTR7 in the reference genomes of primates. The order of LTR7 copies is the same to (G). I) TFBSs on each LTR7 copy. The order of LTR7 copies is the same to (G). J) TF-binding motifs at positions corresponding to HSREs on each LTR7 copy. The order of LTR7 copies is the same to (G). Black and gray colors respectively indicate the presences of motifs with p values of <0.0001 and <0.001, identified by FIMO [64]. K) Enrichment of sequence reads mapped to LTR7 copies belonging to respective subgroups. The Y-axis shows reads per million (RPM) relative to that of input control. L) Insertion dates of proviruses of HERVH/LTR7 along with the species tree of primates. Upper panel: The boxplot showing insertion dates of the respective proviruses estimated by sequence comparison between 5′- and 3′-LTRs. Insertion dates of the proviruses are separately shown in the respective subgroups. Categories of subgroups I, II, and III contained 66, 248, and 227 copies of proviruses, respectively. Lower panel: Phylogenetic tree of primates with time scale. The tree was obtained from TIMETREE [72]. Red branch in the tree indicates the period when the rewiring of the core regulatory network of pluripotent cells seems to have occurred.

Characteristics of HSREs in LTR7 identified from the Roadmap dataset are shown in Fig 3. LTR7 showed low genomic mappability (S3B Fig), and, therefore, the results of all-read TFBSs were considered (those of unique-read TFBSs are shown in S7 Fig). LTR7 is an LTR sequence of the HERVH provirus belonging to the ERV1 family. In our clustering analysis, LTR7 belonged to the HERV_2 cluster, whose members were highly bounded by SOX2, POU5F1, and NANOG (Fig 2). These TFBSs were observed at approximately the same consensus positions of LTR7 among those copies (Fig 3A and 3B). For example, a peak of SOX2 binding was observed at around the 150^th^ nucleotide position on the consensus sequence of LTR7 (Fig 3B). Splits of HERV-TFBS peaks were observed in NANOG, EOMES, and FOXA1/2 due to an insertion/deletion in multiple sequence alignment of LTR7 (S8 Fig). TF-binding motifs in HERV-TFBSs were observed at approximately the same consensus position of LTR7 among those copies (Fig 3C and 3D). We identified HSREs according to the scheme described in Fig 1 (and Materials and Methods). To identify HSREs, we compared heights of the peaks between HERV-TFBSs and TF-binding motifs (S9 Fig). If the peak of TF-binding motifs (Fig 3C and 3D) was higher than 60% of that of HERV-TFBSs (Fig 3A and 3B), the set of TF-binding motifs was regarded as HSRE. We identified novel HSREs in LTR7, such as EOMES, FOXA1/2, and GATA6, and confirmed the previous reports showing that NANOG-, SOX2-, and POU5F1-binding sites were shared across the LTR7 copies [10–13]. Although the HSREs of NANOG, EOMES, FOXA1, and SOX2 were recaptured from unique-read TFBSs, the peaks of HERV-TFBSs in unique-read TFBSs were substantially lower than those in all-read TFBSs (S7 Fig). Chromatin accessibilities evaluated by DHSs and chromatin states [47–49] showed that the regulatory elements of LTR7 were specifically active in ES cells (Fig 3E and 3F), consistent with the results of previous studies [9–12, 50].

### Heterogeneity of regulatory elements in LTR7

To approach the evolutionary dynamics of HERV/LTR regulatory elements, we investigated heterogeneity of the regulatory elements. We focused on HSREs that was disproportionately present in a specific subgroup of a HERV/LTR type. LTR7 copies were classified into three main subgroups (subgroups I, II, and III) by phylogenetic analysis based on the sequences (Fig 3G). Examining orthologous copies of LTR7 in primates indicated that these subgroups were inserted at different time points (Fig 3H). NANOG- and EOMES-binding sites were uniformly present among the three subgroups (Fig 3I). SOX2- and POU5F1-binding sites were found to be enriched in subgroup III, and FOXA1-binding sites (and, to a certain extent, FOXA2- and GATA6-binding sites) were enriched in subgroup II (Fig 3I). We referred to the ChIP-Seq dataset provided by Ohnuki *et al*. [10] (S10 Fig) because this dataset contained ChIP-Seq of SOX2, POU5F1, and KLF4 in iPS cells, and the sequence read lengths (75-bp) were much longer than those of ENCODE/Roadmap dataset (25- or 36-bp). We also referred to the ChIP-Seq data of NANOG in ES cells provided by Durruthy-Durruthy *et al*. [15], performing 100-bp pair-ended sequencing (S10 Fig). Genomic mappability of LTR7 substantially improved in the 75-bp sequencing compared with 36-bp (S10A Fig). In this dataset, we demonstrated that binding of SOX2, KLF4, and POU5F1 were enriched in subgroup III (S10D Fig). In particular, the enrichments were observed in both all- and unique-read TFBSs. POU5F1-binding motifs at positions corresponding to HSREs were enriched in subgroup III, while FOXA1/A2-binding motifs were excluded (Fig 3J). To quantitatively compare TF binding among the subgroups, we counted the number of reads mapped on LTR7 copies and summed them in respective subgroups, and then we estimated the enrichment of the reads to input control in respective subgroups (Fig 3K). In NANOG and EOMES, the enrichment was relatively higher in subgroup III although the reads were enriched in all the three subgroups. In SOX2 and POU5F1, the reads were enriched in subgroup III. In FOXA1 (and, to a certain extent, in FOXA2- and GATA6-binding sites), the reads were enriched in subgroup II. Thus, we demonstrated subgroup-specific TF binding in LTR7. In a previous study, LTR7 copies were divided into transcriptionally active and inactive groups based on RNA-Seq using pluripotent cells [11]. We further demonstrated that the active LTR7 copies were enriched in the subgroup III (S11 Fig). Some LTR7 copies fuse with host coding/noncoding genes and play an essential role in maintenance of cell pluripotency [10–12, 39]. We demonstrated that most of the LTR7 copies comprising the chimeric transcripts belonged to the subgroup III (S11 Fig). Finally, we attempted to estimate insertion dates (i.e., ages) of proviruses of HERVH/LTR7 based on sequence comparison between 5′- and 3′-LTRs (see Materials and Methods). As shown in Fig 3L, majority of the subgroup I, II, and, III seem to have been inserted in branch of the genera *Catarrhini* and *Hominoidea* and the span from the end of *Hominoidea* to the beginning of *Homininae* (interquartile range of insertion dates; 29.7–42.0, 19.4–31.1, and 13.5–22.7 million years ago (Mya), respectively). This is consistent with the insertion dates estimated by presence of orthologous copies in primates (Fig 3H).

### Changes in regulatory elements during LTR5 evolution

**Fig 4:**
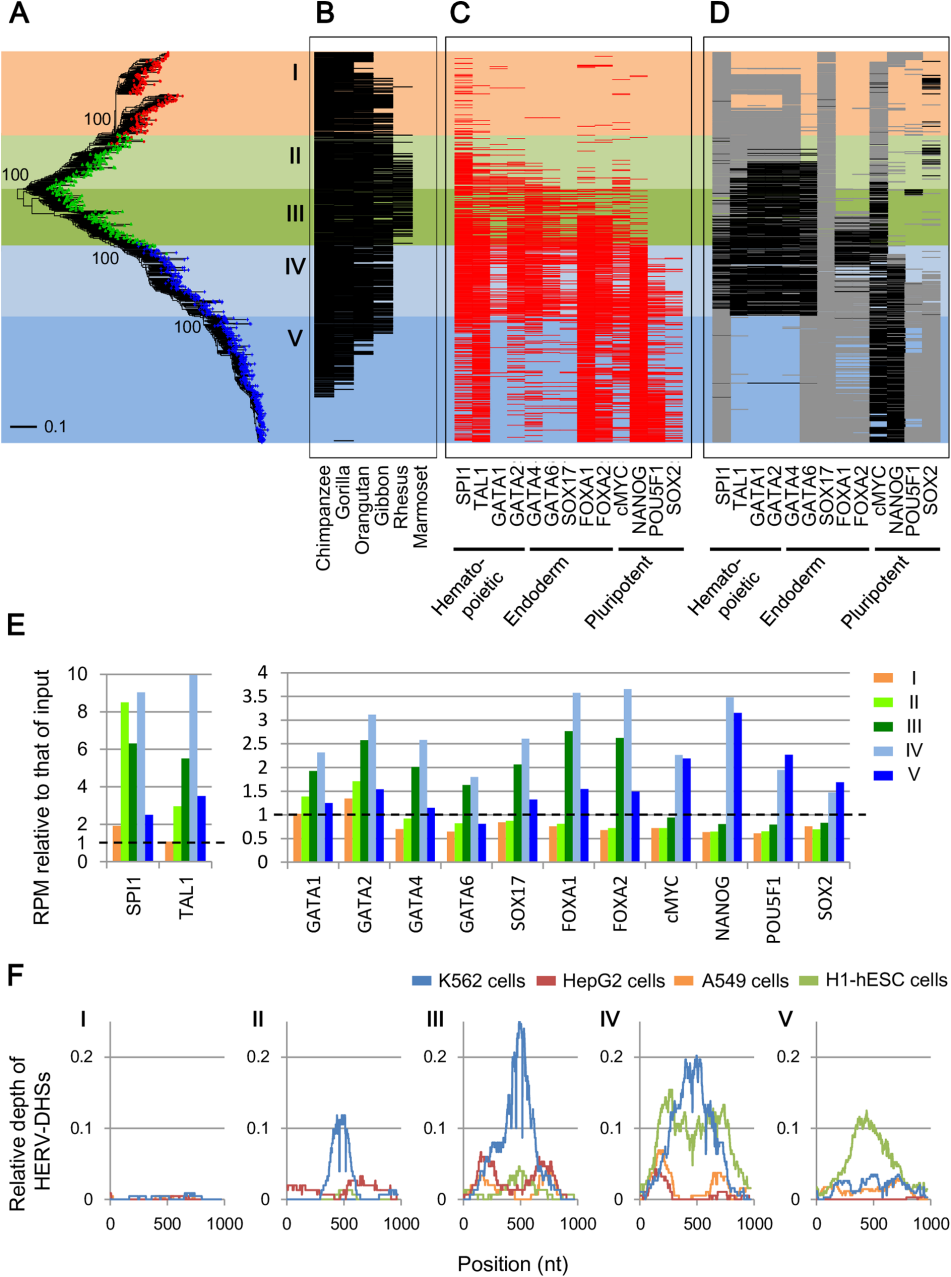
Changes in regulatory elements in LTR5 group. Results from all-read TFBSs are shown. A) The unrooted phylogenetic tree of LTR5A (red), LTR5B (green), and LTR5_Hs (blue) copies constructed using the maximum likelihood method. LTR5 was divided into five groups (I–V) based on the tree and their TFBSs (shown in (C)). Fragmented and outlier copies were excluded from the analysis. Copies of 233, 300, and 532 respectively belonging to LTR5A, LTR5B, and LTR5_Hs were included in the tree (out of 265, 431, and 645, respectively). Representative bootstrap values are shown at the corresponding nodes. B) Orthologous copies in the reference genomes of primates. The order of LTR5 copies is the same to (A). C) TFBSs present on each copy; representative TFBSs are shown. TFBSs of SPI1, TAL1, and GATA1/2 were from the ENCODE dataset, and others were from the Roadmap dataset. The order of LTR5 copies is the same to (A). D) TF-binding motifs at positions corresponding to HSREs on each LTR5 copy. The order of LTR5 copies is the same to (A). Black and gray colors respectively indicate the presence of motifs with p values of <0.0001 and <0.001, as identified by FIMO [64]. E) Enrichment of sequence reads mapped to LTR5 copies belonging to respective subgroups. The Y-axis shows RPM relative to that of the input control. F) Relative number of HERV-DHSs mapped on each consensus position. The X-axis indicates nucleotide position in the consensus sequence of LTR5_Hs. The Y-axis indicates proportion of HERV/LTR copies harboring HERV-DHSs at each position.

We showed that regulatory elements of HERV/LTRs were different within the same HERV/LTR type (Fig 3G–3K). In order to approach evolutionary dynamics of regulatory elements in HERV/LTRs, we examined changes in the regulatory elements in the LTR5 (HERV-K/HML-2) group. LTR5 is composed of LTR5A, LTR5B, and LTR5_Hs. LTR5_Hs is the youngest HERV/LTR type, and a previous study reported that LTR5_Hs has regulatory elements for POU5F1, SOX2, and NANOG [21]. Also consistent with the results of a previous study [51], phylogenetic analysis and examination of orthologous copies indicated that LTR5B was the oldest ancestral type, and LTR5A and LTR5_Hs were independently generated from LTR5B-like viruses (Fig 4A and 4B). Here, we divided LTR5 into five groups (groups I–V) based on their phylogenetic relationship and the TFs binding to them (Fig 4A, 4C and 4E). Group I was rarely bounded by TFs (Fig 4C and 4E). Group II was bounded by SPI1, TAL1, and GATA1/2, which are vital in hematopoietic cells. Group III was bounded by GATA4/6, SOX17, and FOXA1/2, essential in embryonic endoderm cells, together with the hematopoietic TFs. Group IV was bounded by NANOG, MYC, POU5F1, and SOX2, which are critical in pluripotent cells, in addition to the hematopoietic and the endoderm TFs. In group V, which is the youngest group, binding levels of some hematopoietic TFs (SPI1 and GATA1/2) and endoderm TFs (GATA4/6 and SOX17) were low. These differences in TF binding correlated with the differences in TF-binding motifs at positions corresponding to the HSREs (Fig 4D). Chromatin accessibilities evaluated by DHSs indicate that the cell specificity of LTR5 members shifted along with their gain/loss of TFBSs (Fig 4F). Group I was not active in any cell types, as expected owing to the absence of the regulatory elements. Group II was active in K562 (leukemia) cells. Group III was active in HepG2 (hepatoblastoma) and A549 (lung epithelial cancer) cells, in addition to K562 cells. Group IV was active in H1-hESC (ES) cells, in addition to the above cells; group V was not active in K562 cells.

### Signatures of the HERV/LTR regulatory elements

We examined chromatin states [47–49] of HERV/LTRs with and without TFBSs/HSREs. Compared with the entire population of HERV/LTRs, HERV/LTRs harboring HERV-TFBSs or HSREs were enriched in promoter [transcription start site (TSS) and promoter flanking regions (PF)], enhancer (E), weak enhancer (WE), and CTCF-binding regions (CTCF), but not in transcribed (T) and repressed (R) regions (S12A Fig). The HERV/LTR types enriched in enhancer regions were different across different cell types (S12B Fig). These differences seem to reflect the differences of their HSREs; LTR2B [9], LTR7 [9, 11, 50], MER41B, and LTR5B, which were respectively enriched in the enhancer regions of GM12878, H1-hESC, K562/HeLa-S3, and HepG2 cells, had HSREs bounded by TFs essential in the corresponding cell types (S6A Fig, Fig 3A and 3B, S6C Fig and S6B Fig, respectively). Unlike enhancers, HERV/LTRs enriched in CTCF-binding regions remained unchanged among the cell types (S12C Fig), which is consistent with previous findings [41].

We examined TFs in which large fractions of TFBSs were occupied by HERV-TFBSs (S3 Table). Binding sites of NFYA/B, USF1/2, GATA4/6, TAL1, SOX2, SOX17, and TCF4 were highly overlapped with HERV/LTRs. Nearly half of NFYB-binding sites were observed on HERV/LTRs [52]. NFYA/B frequently bound to members of the HERV_4 cluster in Fig 2 (e.g., LTR12, MER51, and MER57 groups) and members of the HERV_6 cluster (MLT1 group) (Fig 2). These HERV/LTRs contained HSREs for NFYA/B [see dbHERV-REs (http://herv-tfbs.com/)].

Then, we investigated specific associations between the insertion dates of HERV/LTRs and TFs that bound to the HERV/LTRs (S13 Fig). HERV/LTRs integrated after the divergence of primates were highly bounded by members of TF_2 (pluripotent cluster) shown in Fig 2, such as POU5F1, SOX2, SMAD1, TCF4, and NANOG (S13 Fig and Fig 2). This is consistent with the results of a previous study showing that SOX2- and POU5F1-binding sites were amplified after the divergence of primates by insertions of HERV/LTRs harboring the binding sites [13]. HERV/LTRs integrated before the divergence of primates were highly bounded by members of the TF_6 cluster, such as SIX5, USF1/2, and ATF3 (S13 Fig and Fig 2). This is because these TFs frequently bound to the MLT1 group (Fig 2), which inserted before the divergence of primates. HERV/LTRs that inserted at the span from *Catarrhini* to *Hominoidea* were highly bounded by NFYA/B and LEF1 (S13 Fig). This is because these TFs bound to the LTR12 group, which inserted at the span from *Catarrhini* to *Hominoidea* [see dbHERV-REs (http://herv-tfbs.com/)].

### Characteristics of host genes in the vicinity of HERV/LTR regulatory elements

**Fig 5:**
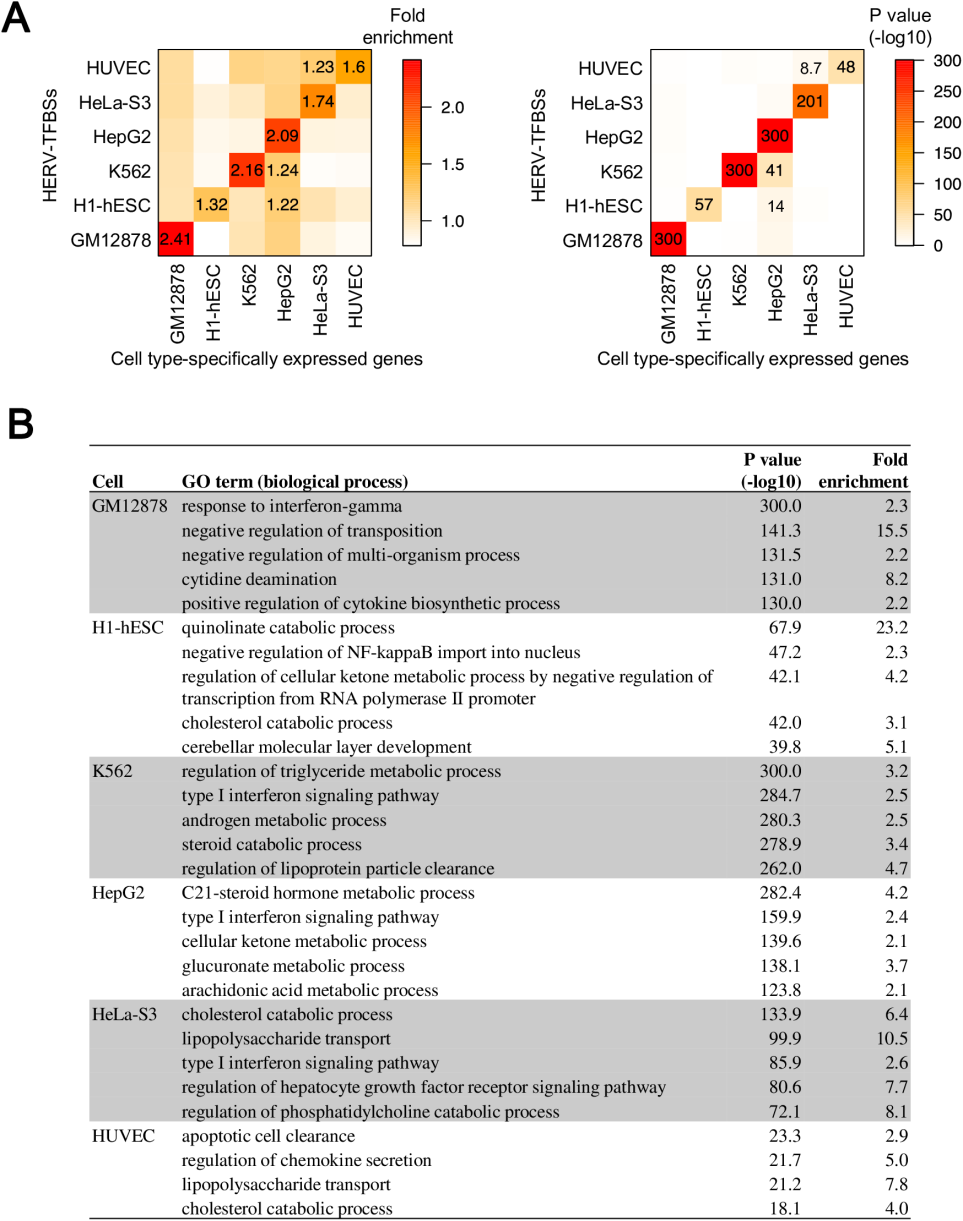
Characteristics of genes in the vicinity of HERV-TFBSs. Results from unique-read TFBSs are shown. A) Enrichment of HERV-TFBSs as seen in regions near cell type-specific genes. In respective cell types, 200 of the specifically expressed genes according to the cell type were identified. Then we measured enrichments of HERV-TFBSs of respective cell types in regions near the cell type-specific genes using the GREAT [53]. Fold enrichment scores (left) and p values (right) are shown as heatmaps. Fold enrichment scores of >1.2 are shown with the corresponding p values. B) Distance-based GO enrichment analysis. GO terms in the category of biological process were examined. The GREAT analyses [53] were performed using sets of all HERV-TFBSs in respective cell types. HERV-TFBSs identified in cells treated with special conditions (e.g., supplement of interferon) were excluded. GO terms were summarized by REVIGO [73]. GO terms with hold enrichment scores of >2 are shown.

It is important to clarify whether HERV-TFBSs contribute to the regulation of host genes, especially in a cell type-specific manner. We examined the association between HERV-TFBSs and genes specifically expressed in a particular cell type. In six cell types (GM12878, H1-hESC, K562, HepG2, HeLa-S3, and HUVEC cells), we identified 200 genes that specifically expressed in each cell type. Subsequently, we examined the enrichment of HERV-TFBSs according to the cell types in regions nearby the genes that were specifically expressed. We demonstrated that HERV-TFBSs in each cell type were enriched in region nearby the specifically expressed genes in the corresponding cell type (Fig 5A). This indicates that HERV-TFBSs are involved in cell type-specific regulation of host genes.

To ascertain which biological functions are associated with HERV-TFBSs/HSREs, we performed Gene Ontology (GO) enrichment analysis with GREAT [53]. First, we performed the analysis using a set of all HERV-TFBSs in one cell type (Fig 5B). HERV-TFBSs in cells such as GM12878 and K562 were highly enriched in regions nearby the genes associated with innate immunity-related pathways such as “response to interferon-gamma” and “type I interferon signal pathway” (Fig 5B). The MER41 and MLT1 groups occupied significant fractions of HERV-TFBSs nearby the genes associated with the above biological processes (S14 Fig; left panel). Regarding TFBSs, binding sites of SPI1, POU2F2, ZNF263, and USF1 were found to be enriched (S14 Fig right panel). Next, we ascertained biological processes in GO term with which HERV-TFBSs were more enriched compared to the other TFBSs (i.e., TFBSs did not overlap with HERV/LTRs). HERV-TFBSs showed significantly stronger associations with biological processes relevant to immune responses compared to the other TFBSs (S4 Table). We also performed GO enrichment analysis to examine biological functions in which HERV-TFBSs were enriched compared to the entire population of HERV/LTRs, and we obtained similar results (S5 Table). Finally, we performed the GO enrichment analyses to infer biological functions with which each type of HSRE is associated. In this analysis, we used sets of HERV-TFBSs harboring each type of HSRE in respective cell types. In total, 39,946 significant associations for combinations of cell types, HSREs, and GO terms were identified [summary data is deposited in dbHERV-REs (http://herv-tfbs.com/)]. Consistent with the above analyses, GO terms associated with the immune response were frequently observed (S6 Table), and the associations between HSREs and various biological processes were identified [see dbHERV-REs (http://herv-tfbs.com/)].

### Long-range interactions between promoters and HERV/LTR regulatory elements

**Fig 6:**
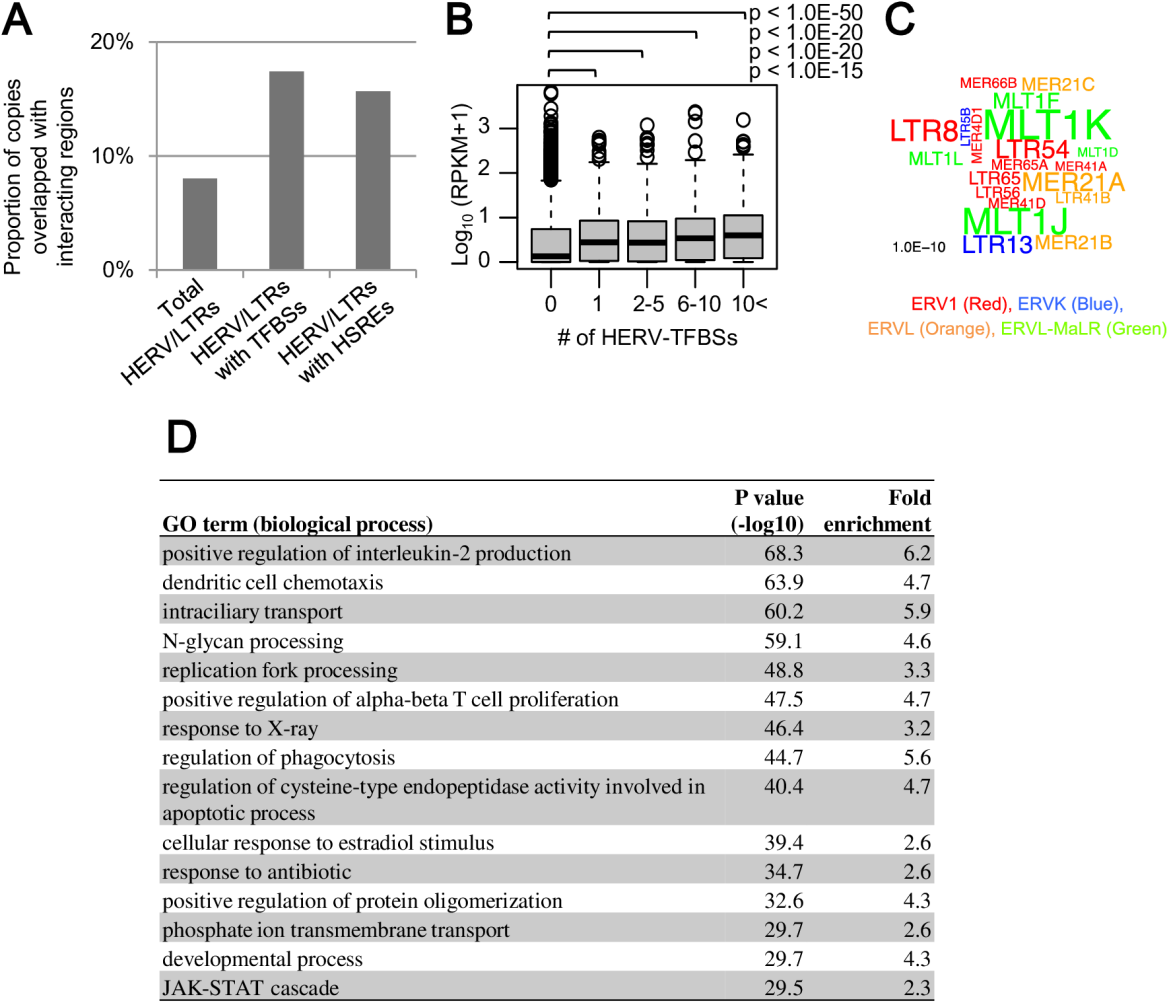
Long-range interactions between HERV-TFBSs/HSREs and promoters of host genes. The interactions were extracted using pcHi-C dataset in GM12878 cells [54, 55]. Results from unique-read TFBSs are shown. A) Proportion of HERV/LTR copies overlapped with promoter-interacting regions. Proportions of total HERV/LTRs, HERV/LTRs with HERV-TFBSs, and HERV/LTRs with HSREs are separately shown. B) Transcription levels (log_10_ (RPKM+1)) of protein-coding genes and number of HERV-TFBSs interacting with the genes. Genes were divided into five categories based on the number of HERV-TFBSs interacting with the genes (0, 1, 2–5, 6–10, and 10<). Categories of the 0, 1, 2–5, 6–10, and 10< respectively contained 13,265, 1,179, 1,946, 822, and 1,639 of genes. P values were calculated using the Mann-Whitney U test with adjustment for multiple tests using the BH method. C) The word cloud indicating HERV/LTR types enriched in the interacting regions. Word sizes are proportional to the −log_10_ (p value) calculated using the Fisher’s exact test. The word colors indicate HERV/LTR families. D) Hi-C-based GO enrichment analysis. A set of all HERV-TFBSs in GM12878 cells was used. HERV-TFBSs identified in cells treated with special conditions (e.g., supplement of interferon) were excluded. GO terms were summarized by REVIGO [73]. GO terms with hold enrichment scores of >2 are shown.

Some regulatory elements affect the remote genes via three-dimensional (3D) interactions by forming chromatin loops [45]. We attempted to extract such 3D interactions between HERV-TFBSs/HSREs and promoters of host genes from the data on promoter-captured Hi-C (pcHi-C) in GM12878 cells [54, 55]. pcHi-C is a modified “chromosome conformation capture” method for a comprehensive identification of the 3D interaction between promoters and other genomic regions [54]. We first examined HERV-TFBSs or HSREs present in promoter-interacting regions (interacting regions). In total, 26,194 and 3,860 of HERV-TFBSs and HSREs-containing HERV-TFBSs, respectively, were present in the interacting regions. Some interacting regions were associated with several genes, and 81,536 or 12,452 of interactions between promoters of genes and HERV-TFBSs or HSREs-containing HERV-TFBSs were identified, respectively. The average interval of interactions between promoters and interacting regions containing HERV-TFBSs was 392 kb (average interval of interactions between promoters and all interacting regions was 411 kb in this dataset). HERV/LTRs harboring TFBSs or HSREs were enriched two-fold in interacting regions compared with the population of the entire HERV/LTRs (Fig 6A). Transcription levels (reads per kilobase per million mapped reads; RPKM) of genes tended to be higher as the number of HERV-TFBSs interacting with the genes increased (Fig 6B). Thus, the HERV/LTR regulatory elements in interacting regions seem to work as transcriptional modulators of host genes via long-range interactions. Members of the MLT1, MER21, and MER41 groups were enriched in interacting regions, together with LTR8, LTR54, and LTR13 (Fig 6C). Next, we developed and performed a “Hi-C-based” GO enrichment analysis by modifying a statistical method used in GREAT [53] (see Materials and Methods). As shown in Fig 6D, HERV-TFBSs were highly enriched in GO terms associated with immune response such as “positive regulation of interleukin-2 production” and “dendritic cell chemotaxis,” consistent with the result of “distance-based” GO enrichment analysis as shown in Fig 5B. Furthermore, using the Hi-C-based GO enrichment analysis, we ascertained biological processes in GO term with which HERV-TFBSs were more enriched compared to the other TFBSs. Consistent with the above results, HERV-TFBSs showed significantly stronger associations with biological processes relevant to immune responses compared to the other TFBSs (S7 Table).

### Construction of dbHERV-REs

We constructed dbHERV-REs, a database of HERV/LTR regulatory elements with an interactive user interface (http://herv-tfbs.com/) (S19 Fig). The database provides (i) general information on HERV/LTRs such as family classification, copy number, and insertion date judged by distribution of orthologous copies among mammalian genome; (ii) positions of HERV-TFBSs, HSREs, and HERV-DHSs in the consensus sequence of HERV/LTRs and in the human reference genome; and (iii) results of GO enrichment analyses with GREAT [53] using sets of respective HSREs. The database also can compare phylogenetic relationship of HERV/LTR copies with the presence of orthologous copies across the mammalian genome, TFBSs, and TF-binding motifs. Results of all- and unique-read TFBSs are available in the database. Additionally, the database provides results on pre-determined TFBSs provided by ENCODE and Roadmap, which were based on their analytical pipelines of ChIP-Seq peak calling (S2 Table). As of May 2017, TFBSs for 97 TFs and DHSs for 125 cell types were deposited. A user can focus on significant associations between HERV/LTRs and TFs by setting statistical and other thresholds.

## Discussion

We showed that HERV/LTRs frequently contained HERV-TFBSs/HSREs for TFs essential in hematopoietic (e.g., SPI1, TAL1, and GATA1/2), pluripotent (e.g., SOX2, POU5F1, and NANOG), and embryonic endoderm/mesendoderm cells (e.g., GATA4/6, SOX17, and FOXA1/2). Hematopoietic regulatory elements of HERV/LTRs seem to descend from ancestral exogenous retroviruses, which would have replicated in the hematopoietic (or blood) cells, considering that modern exogenous retroviruses frequently contain such regulatory elements [38]. Pluripotent regulatory elements seem to have been crucial for efficient replication of HERV/LTRs in germ cells, as with other TEs such as LINE1, because transcriptional environments are similar between pluripotent and early embryonic cells [21, 56]. Endoderm/mesendoderm regulatory elements also seem to be important for HERV/LTRs, possibly for their replication in the host germ cells immediately after the endogenization, as these TFs highly expressed in both somatic and germ cells [41]. A previous study showed that the regulatory elements of HERV/LTRs are active in various cells and tissues by evaluating enrichment of active histone modifications on HERV/LTRs [50]. Therefore, as the number of available ChIP-Seq datasets increase, a greater number of regulatory elements of HERV/LTRs will be identified.

Although the role of retroviral internal sequences in transcription remains unclear, it is known that an internal sequence in Human T-cell Leukemia Virus Type 1 (HTLV-1) contains a CTCF-binding site functioning as an insulator [57]. In the present study, we found that a substantial fraction of HSREs was present in the internal sequences, and the most frequently observed HSRE in the internal sequences was the CTCF-binding site (S5I Fig). These findings suggest that regulatory elements, particularly CTCF-binding sites, would be present in the internal sequences of retroviruses, including HERVs, more than previously considered [38, 57]. Further investigation is needed for clarifying the role of retroviral internal sequences in transcriptional modulation.

Pluripotent regulatory elements seem to be essential for HERVs and other TEs to replicate efficiently in the host germ cells and to expand in the host genome. However, the pluripotent regulatory elements are rarely observed in exogenous retroviruses, even though HERVs descended from ancient exogenous retroviruses [38]. In this study, we demonstrated the heterogeneity of regulatory elements among subgroups in LTR7 (Fig 3G–3K), LTR5 group (Fig 4), LTR6A (S15 Fig), LTR9 (S16 Fig), MER11C (S17 Fig), and MER11B (S18 Fig). Such heterogeneity of regulatory elements was also observed in endogenous retroviruses (ERVs) of other mammals [58, 59]. These indicate that gains or losses of the regulatory elements occurred during genomic expansions of the HERV/LTRs (or the ERVs). We observed a tendency that younger subgroup of HERVs had more regulatory elements for pluripotent TFs (e.g., NANOG, POU5F1, and SOX2) in LTR7, LTR5_Hs, LTR6A, and MER11C (Fig 3G–3K, Fig 4, S15 Fig, and S17 Fig, respectively) although we observed an opposite tendency in MER11B (S18 Fig). Thus, HERVs seem to have frequently acquired pluripotent regulatory elements. We hypothesize that these HERVs acquired the pluripotent regulatory elements after endogenization for efficient replication and genomic expansion in the host germ cells. Thus, investigation of heterogeneity of regulatory elements of HERV/LTRs can illuminate the evolutionary dynamics of transcriptional modulation system of HERVs.

LTR7 is essential for the maintenance of pluripotency in ES and iPS cells, and it has been hypothesized that LTR7 insertions rewired the core regulatory network of the pluripotent cells [10–12]. We further clarified the heterogeneity among subgroups of LTR7 with respect to insertion dates, TF binding profiles, and transcriptional activities. Subgroup III, the youngest subgroup of LTR7, was most frequently bounded by SOX2, POU5F1, and KLF4 (Fig 3G–3K and S10D Fig). Subgroup III also showed the highest enrichment of ChIP-Seq reads of NANOG (Fig 3K). Subgroup III showed the highest transcriptional activity in pluripotent cells (S11 Fig). Most LTR7-chimeric transcripts, which are vital in maintaining pluripotency [10–12, 39], were composed of LTR7 belonging to the subgroup III (S11 Fig). These findings suggest that the evolutionary rewiring of the core regulatory network of pluripotent cells was caused by a specific population of LTR7, i.e., members of the subgroup III, rather than by the entire population of LTR7 (Fig 3L). Moreover, this rewiring seems to have occurred more recently than previously thought [60], the branch from the end of *Hominoidea* to *Homininae*. This is because the rewiring should have occurred during the period when subgroup III was inserted (Fig 3G, 3H and 3L). Further investigation is needed to elucidate the evolution of pluripotent cells due to LTR7 insertions.

The GO enrichment analysis based on genomic positions of HERV-TFBSs/HSREs demonstrated that HERV-TFBSs/HSREs tend to be located near the genes involved in innate immune responses such as cytokine-mediated signaling (Fig 5B and S4, S5 and S6 Tables). This tendency was recaptured by Hi-C-based GO analysis, which used information on 3D interactions between HERV-TFBSs and promoters of host genes in B-lymphocytes (GM12878 cells) (Fig 6D). In those GO enrichment analyses, HERV-TFBSs showed significantly stronger associations with biological processes relevant to innate immune responses compared to the other TFBSs (S4 and S7 Tables). This suggests that HERV/LTR regulatory elements were likely to be associated with regulatory networks controlling innate immune responses. Furthermore, this tendency seems to be more attributable to natural selection of HERV/LTRs after the insertions than preferential insertions in specific genomic regions, because HERV/LTR copies with TFBSs were more enriched in regions near the genes related to innate immune response than HERV/LTRs without TFBSs (S5 Table). The tendency of regulatory elements of HERV/LTRs being associated with innate immune response seemed to be affected by cell types (e.g., B-lymphocytes) in which ChIP-Seq was performed. Therefore, as the number of cell types in which ChIP-Seq are performed increase, more associations between HERV/LTRs with TFBSs and specific biological functions will be identified. Finally, GO enrichment analyses showed that each type of HSRE was statistically associated with various biological processes in addition to the immune response [deposited in dbHERV-REs (http://herv-tfbs.com)]. Further research, especially knockout-based studies such as the one by Chong *et al*. [14], is necessary to prove the causal relationship between regulatory elements of HERV/LTRs and regulatory networks controlling specific biological processes.

To summarize, we identified various HERV/LTR regulatory elements involved in several host regulatory networks. Our study provides the foundation to understand the impact of HERV/LTRs on host transcription, and provides insights into transcriptional modulation systems that HERV/LTRs and ancestral retroviruses of HERVs originally used.

## Materials and methods

### Datasets

Information on the ChIP-Seq dataset is summarized in the “peak calling of ChIP-Seq” section. RepeatMasker output file (http://hgdownload.soe.ucsc.edu/goldenPath/hg19/bigZips/chromOut.tar.gz) was downloaded from the UCSC genome browser (https://genome.ucsc.edu/). This is an annotation file of repetitive elements on the human reference genome (GRCh37/hg19) used in RepeatMasker track in the genome browser. Consensus sequences of HERV/LTRs were obtained from the RepeatMasker library (20140131 release) and Repbase Update (1.1.3 release) in Repbase (http://www.girinst.org/server/RepBase/). DHS datasets were obtained from ENCODE (S10 Table). Genome segmentations in six cell types (combined between ChromHMM and Segway) [47–49] were obtained from ENCODE (S11 Table). Datasets of Cold Spring Harbor Laboratory (CSHL) LongPolyA RNA-Seq were obtained from ENCODE in the GTF format (S12 Table). Ontology file (go-basic.obo, date; 3/16/2016) and GO association file (gene_association.goa_human, submission date; 3/16/2016) were downloaded from the GO Consortium (http://geneontology.org/). The UCSC known genes were downloaded from UCSC (http://hgdownload.cse.ucsc.edu/goldenPath/hg19/database/knownGene.txt.gz). pcHi-C dataset in GM12878 cells [54, 55] (GSE81503_GM12878_PCHiC_merge_final_seqmonk.txt.gz and GSE81503_GM12878_PCHiC_merge_final_washU_text.txt.gz, accession GSE81503) were obtained from the Gene Expression Omnibus (http://www.ncbi.nlm.nih.gov/geo/).

### Peak calling of ChIP-Seq

An analytical pipeline used in this study is summarized in S1B Fig. For the Roadmap dataset, we obtained a sequence read file (fastq format) from the Sequence Read Archive (SRA) using the SRA Toolkit fastq-dump (http://www.ncbi.nlm.nih.gov/books/NBK158900/). For the ENCODE dataset, we downloaded an unfiltered alignment file, if available, for GRCh37/hg19 (bam format) from the ENCODE database (http://www.encodeproject.org/). The unfiltered alignment file was generated using the ENCODE Processing Pipeline with BWA 0.7.10 (aln and samse). If the unfiltered alignment file was not available, we downloaded a fastq file from the ENCODE database. Fastq or bam files of biological replicates were then concatenated. Sequence reads in the fastq files were mapped to human reference genome (GRCh37/hg19) using BWA 0.7.12 (aln and samse/sampe). In the default setting of BWA aln, a multiple mapped read is randomly assigned to a particular genomic position chosen from candidate positions. For the all-read TFBSs, ChIP-Seq peaks were called using MACS2 with default setting. For unique-read TFBSs, multiple mapped reads or reads with low mapping quality (reads with MAPQ score of <10) were removed using samtools view [61], and then ChIP-Seq peaks were called. In peak calling, input control file was used with ChIP-treated file. Information on the ChIP-Seq data is summarized in S8 and S9 Tables.

### Identification of HERV-TFBSs and HSREs

HERV-TFBSs and HSREs were identified separately in ENCODE and Roadmap datasets. HERV-TFBSs and HSREs were identified both in all- and unique-read TFBSs.

We identified HERV-TFBSs in respective cell types by examining the overlaps between HERV/LTRs and TFBSs with bedtools intersect [62]. In the respective TFs, TFBSs or HERV-TFBSs among all cell types or conditions were merged with bedtools merge [62] (referred to as the merged TFBSs or HERV-TFBSs). For counting TFBSs and HERV-TFBSs, the merged TFBSs and HERV-TFBSs were used.

For the identification of HSREs, the merged HERV-TFBSs were used. First, sequences of HERV/LTR copies were extracted from human reference genome (GRCh37/hg19) using bedtools getfasta [62]. Multiple sequence alignment (MSA) of HERV/LTR copies was constructed with a consensus sequence of the corresponding HERV/LTR type. MAFFT v7.239 [63] was used for the construction of MSA with the options—addfragments,—keeplength, and—retree 2. In this setting, the consensus sequence was used as input, and sequences of HERV/LTR copies were used as fragment sequence. In MSA, the position of HERV-TFBSs was mapped on each HERV/LTR sequence, and then the number of the mapped HERV-TFBSs was counted at every consensus position (referred to as “depth” of HERV-TFBSs). For setting the threshold to identify peaks of HERV-TFBSs, randomized (shuffled) TFBS datasets were generated with bedtools shuffle [62] for 500 times. In the respective randomized datasets, the depth of HERV-TFBSs was counted for each consensus position with the above-mentioned procedures. For every consensus position, average and standard deviation of the depth of HERV-TFBSs among randomized datasets was calculated. Standardized score (z score) of HERV-TFBS depth was calculated for every consensus position with the average and standard deviation in randomized datasets (termed as base-wise z score). If base-wise z score of a given region (>50-bp) in the consensus sequence was higher than four, the region was defined as a peak of HERV-TFBSs. Finally, known TF-binding motifs of the corresponding TF were scanned in original HERV-TFBS sequences. For motif scanning, FIMO [64] and known TF-binding motifs recorded in JASPAR [65] and HOCOMOCO [66] were used. The threshold (p value) of the motif scanning was set at 0.001. In MSA, position of the TF-binding motif was mapped on each HERV/LTR sequence, and then the number of the mapped motifs was counted at every consensus position (referred to as “depth” of TF-binding motifs). To identify HSREs, heights of peaks of depths were compared between HERV-TFBSs and TF-binding motifs. If the height of the TF-binding motif peak is (i) greater than or equal 10 and (ii) greater than 60% of the height of the HERV-TFBS peak, we regard the set of TF-binding motifs as HSRE (S9 Fig). For counting the number of genomic positions of HSREs, overlapping HSREs of the same TF were merged for avoiding double counts. This is because some TF-binding motifs were present in both strands at approximately the same positions due to their palindrome signatures.

After identifying HSREs, overlaps between HSREs and HERV-TFBSs in respective cell types were examined, and the cell specificities of HSREs were determined.

### Randomization test shuffling genomic positions of TFBSs

HERV-TFBS overlaps were counted for all combinations. In each dataset of TFBS, we generated 100 times of randomized TFBS datasets using bedtools shuffle [62] and counted the number of HERV-TFBS overlaps in the randomized datasets. Among the randomized datasets, average and standard deviation of numbers of HERV-TFBS overlaps were calculated. In each HERV-TFBS combination, we calculated z score (count-based z score) using the number of HERV-TFBS overlaps in an observed dataset and the average and standard deviation among randomized datasets.

For TEs other than HERV/LTRs, z scores for all combinations of respective TE types and the merged TFBSs were calculated using the same procedures.

### Hierarchical clustering

We used unique-read TFBSs, and separately dealt with TFBSs of the same TF in distinct cell types. If there were several TFBS files for the same ChIP-Seq condition, the TFBS files were merged using bedtools merge [62]. All TFBSs (e.g., SOX2-binding sites in HUES64 cells from Roadmap) were used for the analysis, except for CTCF-binding sites; we used CTCF-binding sites that were determined in tier 1 and 2 cells of ENCODE (GM12878, H1-hESC, K562, HepG2, HeLa-S3, and HUVEC), HUES64 cells, and germ layer (ectoderm, endoderm, mesoderm, and mesendoderm) cells that were differentiated from the HUES64 cells. Z scores were calculated using the method in the “randomization test shuffling genomic positions of TFBSs” section. A matrix containing the z scores was created. HERV/LTR type whose copy number was less than 100 was excluded from the matrix. Rows (TFBSs) and columns (HERV/LTRs) were excluded if they did not contain any elements whose z scores were greater than or equal to 10. Distance matrix was constructed using the Euclid method based on the z score matrix. We performed hierarchical clustering with the distance matrix using Ward’s method. All analyses were performed by packages of amap and ReorderCluster implemented in R.

### Phylogenetic analyses

As listed in S13 Table, phylogenetic trees were constructed for HERV/LTR types satisfying the following criteria: (i) after removal of the fragmented copies (described below), the number of copies fell within the range of 10–2,500; and (ii) greater than 30% of their copies remained after the removal of fragmented copies. Fragmented and outlier copies were excluded from the analysis. For defining the fragmented copies, we constructed preliminary MSA of HERV/LTR copies with the consensus sequence using MAFFT v7.239 [63] with options of—addfragments,—keeplength, and—retree 2 (in this setting, the consensus sequence was used as input, and sequences of HERV/LTR copies were used as fragment sequence). HERV/LTR copies were defined as fragmented if less than 80% of their sequences were only aligned to the consensus sequences in the preliminary MSA. After the removal of fragmented copies, we constructed MSA of HERV/LTR copies using MAFFT v7.239 with—auto options. Sites in the MSA containing gaps were excluded if site coverages of those positions were less than 30%. For defining the outlier copies, a preliminary tree was reconstructed with RAxML v8.2.0 [67]. GTRCAT was used as a nucleotide substitution model. Z score of the length of external branch was calculated for the preliminary tree. Outlier copy, whose z score of the branch length was greater than three, was excluded from the MSA. We constructed the final tree using the same procedures with the preliminary tree. Supporting values were calculated using the SH-like test [68]. In addition to the SH-like test, rapid bootstrap analysis [67] (100 times) was performed for the phylogenetic tree of the LTR5 group.

### Estimation of the insertion dates of HERVH/LTR7 copies

The age of a provirus of ERVs can be estimated by sequence comparison between 5′- and 3′-LTRs of the ERVs, as sequences of both LTRs were identical at the time of insertion, and after the insertion, both LTRs independently accumulated mutations as a part of the host genome [69]. In this analysis, we used the annotation of a provirus of HERVH/LTR7 as reported previously [11]. We only analyzed proviruses of HERVH/LTR7 harboring two LTR7 sequences that were categorized in the same subgroup in the tree (Fig 3G). For each provirus, a pairwise sequence alignment of 5′- and 3′-LTRs was constructed using the EMBOSS Stretcher program [70]. After removal of all gapped sites in the alignment, p-distance of the paired LTRs was calculated, and then the genetic distance of the paired LTRs was computed using the Jukes-Cantor 69 model. A substitution rate of HERVs of 1.0 × 10^−9^ per site per year was used as described previously [71]. Insertion date of the provirus was calculated with the formula, D/2R (D, genetic distance of the paired LTRs; R, substitution rate of HERVs).

### Insertion date (i.e., age) judged by distribution of orthologous HERV/LTR copies in the mammalian genome

For judging whether an orthologous copy of a HERV/LTR copy was present in a certain reference genome, liftOver (http://hgdownload.soe.ucsc.edu/admin/exe/linux.x86_64/liftOver) was used. If liftOver successfully converted the genomic position of a particular HERV/LTR copy in human reference genome to that of a reference genome of other species, we judged an orthologous copy of the HERV/LTR copy was present in the genome of the corresponding species. A minimum match parameter was set at 0.5. Reference genomes of PanTro4 (chimpanzee), GorGor3 (gorilla), PonAbe2 (orangutan), Nomleu3 (gibbon), RheMac3 (rhesus macaque), CalJac3 (marmoset), TarSyr1 (tarsier), MicMur1 (mouse lemur), Mm9 (mouse), Bostau7 (cow), and CanFam3 (dog) were used.

Classification of insertion date of HERV/LTRs was defined as follows: ~*Hominoidea*; greater than 10% of orthologous copies of the HERV/LTR type present in any of the chimpanzee, gorilla, orangutan, and gibbon genomes but absent in that of the rhesus macaque. *Catarrhini*; greater than 10% of orthologous copies of the HERV/LTR type present in the chimpanzee, gorilla, orangutan, gibbon, and rhesus macaque genomes but absent in that of the marmoset. *Simiiformes*; greater than 10% of orthologous copies of the HERV/LTR type present in the chimpanzee, gorilla, orangutan, gibbon, rhesus, and marmoset genomes but absent in those of the tarsier and mouse lemur. *Primates*; greater than 10% of orthologous copies of the HERV/LTR type present in the chimpanzee, gorilla, orangutan, gibbon, rhesus, marmoset, tarsier, and mouse lemur genomes but absent in those of the mouse, cow, and dog. *Eutheria*~; greater than 10% of orthologous copies of the HERV/LTR type present in the chimpanzee, gorilla, orangutan, gibbon, rhesus, marmoset, tarsier, mouse lemur, mouse, cow, and dog genomes. We only analyzed HERV/LTR types whose copy numbers were greater than or equal to 100.

### Gene ontology enrichments analysis

Unique-read TFBSs were used in GO enrichment analyses. GO associations described in gene_association.goa_human were used. GO term associated with greater than or equal to five genes was used in the analyses.

In distance-based GO enrichment analysis, the createRegulatoryDomains command in the local version of GREAT [53] was used for defining regulatory domains of respective GO terms with the option of basal (five kb upstream and one kb downstream of the TSS) plus extension (up to one Mb). We used the TSS annotation based on the UCSC known genes. Enrichment score and p values with binomial test were calculated by the original R script.

To determine the GO term in which TFBSs with HERV/LTRs were more enriched than the other TFBSs (TFBSs not on HERV/LTRs), we counted the number of TFBSs with HERV/LTRs and the entire TFBSs in regulatory domains associated with a certain GO term. Then, the enrichment significance was calculated by Fisher’s exact test.

In order to examine the GO term in which HERV/LTRs harboring TFBSs were more enriched than the entire HERV/LTRs (all HERV/LTRs regardless of overlaps with TFBSs), we estimated the number of HERV/LTRs harboring TFBSs and the entire HERV/LTRs overlapped to regulatory domains associated with a certain GO term. The enrichment significance was calculated by Fisher’s exact test.

To ascertain the GO term in which each type of HSRE was enriched, we performed the GREAT analysis [53] using a set of HERV-TFBSs harboring a HSRE in each cell type. The threshold for statistical significance was set at 0.1, with false discovery rates calculated using the Benjamini–Hochberg (BH) method.

We thus developed the “Hi-C-based” GO enrichment analysis by modifying the GREAT algorithm [53]. Interacting regions in pcHi-C [54, 55] of all genes were merged using bedtools merge [62] and were defined as “total region”. Interacting regions of genes associated with a particular GO term were merged and were defined as “regulatory domain” for the corresponding GO term. The lengths of the total region and regulatory domain were calculated (termed total_length and regdom_length, respectively). HERV-TFBSs overlapping with the total region and regulatory domain were also counted (termed total_count and regdom_count, respectively). For calculating the enrichment significance, we performed a binomial test using the above total_count and regdom_count in addition to the ratio of regdom_length and total_length (regdom_length/total_length).

In Hi-C-based GO enrichment analysis, we performed GO enrichment analysis to determine the GO term in which TFBSs with HERV/LTRs were more enriched than the other TFBSs. We counted the number of TFBSs with HERV/LTRs and the other TFBSs in regulatory domains associated with a certain GO term. Then, the enrichment significance was calculated by Fisher’s exact test.

### Enrichment of HERV-TFBSs near the cell type-specifically expressed genes

In CSHL LongPolyA RNA-Seq, protein-coding genes with RPKM >3 in any cell type were included in the analysis. For every gene, z score of RPKM was calculated for each cell type by using the average and standard deviation of the six cell types (GM12878, H1-hESC, K562, HepG2, HeLa-S3, and HUVEC cells). Regarding the z scores, top 200 genes in each cell type were defined as those expressed specifically in the corresponding cell type. Regulatory domain for genes specifically expressed in a certain cell type was created by using the createRegulatoryDomains command in GREAT [53] with a setting of basal (5 kb upstream and 1kb downstream of TSS) plus extension (up to 1 Mb). Enrichment scores and p values with binomial test were calculated by original R scripts.

### Construction of dbHERV-REs

The system is running on Amazon Web Service (http://aws.amazon.com/). The relational database was constructed with MySQL. The server program was written in Python using Twisted (http://twistedmatrix.com/), an event-driven networking framework. The user interface was designed upon AJAX (Asynchronous JavaScript + XML) philosophy. plotly.js (http://plot.ly/javascript/) is used for data visualizations. jQuery (http://jquery.com/) was used for the browser scripting.

## Supporting information

S1 Fig An analytical pipeline for peak calling of ChIP-Seq. A). TFs for which ChIP-Seq was performed in this study. ChIP-Seq data for MYC, CTCF, FOXA1, FOXA2, HNF4A, NANOG, POU5F1, PRDM1, and SP1 were provided by ENCODE and Roadmap. ChIP-Seq data for other TFs were provided by either ENCODE or Roadmap. Detailed information is summarized in S1 Table. B) An analytical pipeline for peak calling of ChIP-Seq. We generated two types of TFBS datasets: all- and unique-read TFBSs. All-read TFBSs are ChIP-Seq peaks called with all reads mapped to the reference human genome. Unique-read TFBSs are ChIP-Seq peaks called with only reads that were uniquely mapped to the reference human genome.
(TIFF)

S2 Fig TFBSs, HERV-TFBSs, and HSREs identified from all- and unique-read TFBSs. A) Proportions of HERV-TFBSs and HERV-TFBSs with HSREs. The left and right panels show results of all- and unique-read TFBSs, respectively. Proportions of HERV-TFBSs harboring HSREs in entire TFBSs (left value) and in HERV-TFBSs (right value) are shown. In the “merged” dataset, TFBSs of the same TF were merged between ENCODE and Roadmap, and were then counted. B) Comparison between the numbers of HSRE types identified from all- and unique-read TFBSs. A dot indicates a HERV/LTR type. C) Comparison between the numbers of HERV-TFBSs harboring HSREs from all- and unique-read TFBSs. A dot indicates a HERV/LTR type.
(TIFF)

S3 Fig Comparison of all- and unique-read TFBSs. A) Comparison between the numbers of HERV-TFBSs of all- and unique-read TFBSs. The comparison was performed in respective ChIP-Seq experiments, and the results for SPI1 in K562 cells from the ENCODE dataset, POU5F1 in HUES64 cells from the Roadmap dataset, and NANOG in HUES64 cells from the Roadmap dataset are shown. In all the three ChIP-Seq experiments, 36-bp single-end sequencing was performed. A dot indicates a HERV/LTR type. In most HERV/LTRs, numbers of HERV-TFBSs were approximately the same. However, in some HERV/LTRs such as LTR5_Hs and LTR7, numbers of HERV-TFBSs was higher for all-read TFBSs than for unique-read TFBSs. B) Distribution of genomic mappability (uniqueness) scores on HERV/LTR sequences. Scores are normalized between 0 and 1, with 1 representing a unique sequence and 0 representing a sequence that occurs more than 4 times in the genome (see http://genome.ucsc.edu/). Mappability score of 36-bp single-end sequencing was calculated with gem-mappability [74]. Average mappability scores of HERV/LTR copies were calculated, and the distribution was shown separately in respective HERV/LTR types. With respect to median value of the mappability score, the worst 50 of HERV/LTR types are shown. C) Comparison between the numbers of HERV-TFBSs of all- and unique-read TFBSs. The comparison was performed in respective HERV/LTR types. Results for MLT1J and LTR5_Hs are shown. A dot indicates a ChIP-Seq experiment. Linear regression was performed, and the slope was indicated. In MLT1J with high genomic mappability (average score = 0.98), numbers of HERV-TFBSs in respective ChIP-Seq experiments are approximately the same for all- and unique-read TFBSs (slope = 1.0). In LTR5_Hs with low genomic mappability (average score = 0.38), numbers of HERV-TFBSs in respective ChIP-Seq experiments tended to be approximately four times higher for all-read TFBSs than for unique-read TFBSs (slope = 4.0). D) Distribution of slopes of linear regressions (mentioned in (C)) in respective HERV/LTRs. The X-axis is log_2_ scale. HERV/LTRs with slopes >2 are listed in the right table. E) Association between the slopes and average values of genomic mappability scores. A dot indicates a HERV/LTR type. The X-axis is log_2_ scale.
(TIFF)

S4 Fig Comparison of HERV/LTRs with other TE classes with respect to TF binding. Results of unique-read TFBSs are shown. A) Number of TFBSs overlapping with respective TE classes. B) Distribution of the number of TFs significantly binding to respective TE types. Out of 106 TFs (79 ENCODE TFs + 27 Roadmap TFs), the number of TFs that are significantly bounded to a TE type was counted. The distribution is separately shown in respective TE classes. Outliers of TE types are not shown. Enrichment significance values were measured using a randomization test shuffling genomic position of TFBSs. TFs with z score >5 and fold enrichment score >2 were considered as significantly binding to the TE type. To statistically compare HERV/LTR with other TEs with respect to the numbers of TFs, Mann-Whitney U test was performed. C) TE types bounded by many TFs.
(TIFF)

S5 Fig Characteristics of HSREs. Results of unique-read TFBSs are shown. A) Distribution of HSREs present in HERV-TFBSs. The Y-axis indicates the number of HERV-TFBSs containing 1, 2, 3, 4, and greater than or equal to 5 HSREs. B) HERV/LTRs that contained many types of HSREs (TFs). C) and D) average divergence of each HERV/LTR type from the consensus sequence and absolute numbers (C) or proportions (D) of copies containing HSREs. Color of a dot indicates insertion period of the HERV/LTR type judged by distribution of orthologous copies in the mammalian genome. E) average divergence of each HERV/LTR type from the consensus sequence and proportions of HERV-TFBSs containing HSREs. Please note the difference in Y-axis between (D) and (E). F) HSREs (TFs) observed in many HERV/LTR copies. G) HSREs (TFs) observed in many types of HERV/LTRs. H) and I) HSREs (TFs) observed in many types of HERV/LTRs classified into LTR (H) and internal sequence (I).
(TIFF)

S6 Fig HSREs identified from unique-read TFBSs. Left panel: number of HERV-TFBSs mapped on each consensus position of LTR2B (A), LTR5B (B), MER41B (C), and MLI1J (D). The X-axis indicates the nucleotide position on the consensus sequence of the corresponding HERV/LTR type. The Y-axis indicates the number of HERV/LTR copies harboring HERV-TFBSs at each position. Right panel: number of TF-binding motifs in HERV-TFBSs mapped on each consensus position of LTR2B (A), LTR5B (B), MER41B (C), and MLI1J (D). The X-axis indicates the nucleotide position of the consensus sequence. The Y-axis indicates the number of HERV/LTR copies harboring the TF-binding motifs at each position. Peaks of the motifs corresponding to HSREs are indicated with an asterisk (*) with motif names.
(TIFF)

S7 Fig Characteristics of HSREs of LTR7 identified from unique-read TFBSs. A) Number of HERV-TFBSs mapped on each consensus position of LTR7. Results of NANOG and EOMES are shown in the left panel, and those of FOXA1, SMAD1, and SOX2 are shown in the right panel. The X-axis indicates nucleotide position of the consensus sequence of LTR7. The Y-axis indicates the number of HERV/LTR copies harboring HERV-TFBSs at each position. B) Number of TF-binding motifs in HERV-TFBSs mapped on each consensus position of LTR7. Results of NANOG and EOMES are shown in the left panel, and those of FOXA1, SMAD1, and SOX2 are shown in the right panel. The X-axis indicates a consensus position of LTR7. The Y-axis indicates the number of HERV/LTR copies harboring the TF-binding motifs in TFBSs at each position. Peaks of the motifs corresponding to HSREs are indicated by an asterisk (*) with motif names (e.g., SOX2 M0). C) Left, phylogenetic tree of LTR7 copies as seen in Fig 3G. Middle, TFBSs on each LTR7 copy. The order of LTR7 copies is the same to the left tree. Right, TF-binding motifs at positions corresponding to HSREs on each LTR7 copy. The order of LTR7 copies is the same to the left tree. Black and gray colors respectively indicate the presence of motifs with p values of <0.0001 and <0.001.
(TIFF)

S8 Fig A split of HERV-TFBS peak due to an insertion/deletion in multiple sequence alignments. Top, number of HERV-TFBSs mapped on each consensus position of LTR7. The split of HERV-TFBS peaks is indicated by an arrow. Bottom left, phylogenetic tree of LTR7. Bottom right, MSA of LTR7. Order of the LTR7 copies is the same to the left tree. A deletion introducing the split was observed in sequences of subgroup II. FOXA1 peak was especially affected by the deletion because FOXA1 disproportionately bound to subgroup II.
(TIFF)

S9 Fig The method to identify HSREs. A) MSA of HERV/LTR copies (blue) harboring HERV-TFBSs (red). TF-binding motifs in HERV-TFBSs are indicated as star marks. B) Number of HERV-TFBSs (red) and TF-binding motifs (black) mapped on each consensus position of the HERV/LTRs. To identify HSREs, peak heights are compared between HERV-TFBSs and TF-binding motifs. If the height of the TF-binding motif peak is greater than 60% of the height of the HERV-TFBS peak, we regard the set of TF-binding motifs as HSRE.
(TIFF)

S10 Fig TFBSs of LTR7 identified in ChIP-Seq with 75-bp single-end or 100-bp paired-end sequencing. ChIP-Seq data on SOX2, KLF4, and POU5F1 (75-bp single-end) was provided by Ohnuki *et al*. [10]. ChIP-Seq data on NANOG (100-bp paired-end) was provided by Durruthy-Durruthy *et al*. [15]. A) Comparison between genomic mappability scores of LTR7 for 36-bp and 75-bp sequencing. B) Number of HERV-TFBSs mapped on each consensus position of LTR7. Results of all- and unique-read TFBSs are shown in the left and right panels, respectively. C) Number of TF-binding motifs in HERV-TFBSs mapped on each consensus position of LTR7. Results of all- and unique-read TFBSs are shown in the left and right panel, respectively. D) Left, phylogenetic tree of LTR7 copies as seen in Fig 3G. Middle and right, TFBSs on each LTR7 copy in all-read (middle) and unique-read (right) TFBSs. The order of LTR7 copies is the same to the left tree.
(TIFF)

S11 Fig LTR7-chmeric transcripts and transcriptional activities of LTR7. Left, the unrooted tree of LTR7 copies as seen in Fig 3G. LTR7 copies fused with ABHD12B, C4orf51, ESRG, HHLA1, LINC-ROR, and LINC00458 [10, 11, 39] are shown with markers. Right, transcriptional activities of LTR7 copies in pluripotent cells as defined by Wang *et al*. [11]. Red, highly active; yellow, moderately active; blue, inactive.
(TIFF)

S12 Fig HERV/LTRs enriched in regions with various chromatin signatures. A) Proportion of HERV/LTR copies overlapped with each chromatin state. Chromatin states were predicted by genome segmentation method [47–49]. Proportions in total HERV/LTRs, HERV/LTRs with HERV-TFBSs, and HERV/LTRs with HSREs are separately shown. Results of unique-read TFBSs are shown. Averages of the proportions among six cells (GM12878, H1-hESC, K562, HepG2, HeLa-S3, and HUVEC) are shown. TSS, promoter region including TSS; PF, predicted promoter flanking region; E, enhancer; WE, weak enhancer or open chromatin cis regulatory element; CTCF, CTCF enriched element; T, transcribed region; R, repressed or low activity region. B) Word clouds showing HERV/LTRs enriched in enhancer regions of each cell type. The word sizes are proportional to −log_10_ (p values) calculated with Fisher’s exact test. The word colors indicate HERV/LTR families. Word clouds were created by wordcloud package implemented in R. C) Word clouds showing HERV/LTR types enriched in CTCF-binding regions of each cell type.
(TIFF)

S13 Fig Proportions in HERV-TFBSs stratified by insertion date. Results of unique-read TFBSs are shown. In respective TFs, HERV/LTRs with TFBSs were stratified by insertion date. TFs in which HERV-TFBSs overlapped with HERV/LTRs at least 1,000 times are shown. The integration date of HERV/LTR types was judged by distribution of orthologous of HERV/LTRs among the mammalian genome (see Materials and Methods). Proportions in all HERV/LTRs are shown at bottom of the figure.
(TIFF)

S14 Fig HERV/LTRs (left) and TFs (right) occupying significantly large fractions in HERV-TFBSs associated with interferon-related biological processes. Regarding biological processes identified in Fig 5B, enrichment significance values of HERV/LTRs and TFs are shown. The word sizes are proportional to −log_10_ (p value) calculated with Fisher’s exact test. The word colors indicate HERV/LTR families.
(TIFF)

S15 Fig Characteristics of HSREs identified in LTR6A from Roadmap dataset. Results of all-read TFBSs are shown except for (G). A) Number of HERV-TFBSs mapped on each consensus position of LTR6A. The X-axis indicates nucleotide position of the consensus sequence. The Y-axis indicates number of HERV/LTR copies harboring HERV-TFBSs at each position. B) Number of TF-binding motifs in HERV-TFBSs mapped on each consensus position of LTR6A. The X-axis indicates nucleotide position of the consensus sequence. The Y-axis indicates number of HERV/LTR copies harboring the TF-binding motifs at each position. Peaks of the motifs corresponding to HSREs are indicated by an asterisk (*) with motif names. C) The unrooted phylogenetic tree of LTR6A copies constructed by maximum likelihood method. Fragmented and outlier copies were excluded from the analysis. In total, 204 (out of 288) of LTR6A copies were included in the tree. Representative supporting values calculated by SH-like test [68] are shown on the corresponding branches. D) Orthologous copies of LTR6A in the reference genomes of other mammals. E) TFBSs on each LTR6A copy. F) TF-binding motifs on each copy at positions corresponding to HSREs. Black and gray colors respectively indicate presence of motifs with p values of <0.0001 and <0.001. G) TFBSs on each LTR6A copy. Results of unique-read TFBSs are shown.
(TIFF)

S16 Fig Characteristics of HSREs identified in LTR9 from Roadmap dataset. Results of all-read TFBSs are shown except for (G). A) Number of HERV-TFBSs mapped on each consensus position of LTR9. The X-axis indicates nucleotide position of the consensus sequence. The Y-axis indicates number of HERV/LTR copies harboring HERV-TFBSs at each position. B) Number of TF-binding motifs in HERV-TFBSs mapped on each consensus position of LTR9. The X-axis indicates nucleotide position of the consensus sequence. The Y-axis indicates number of HERV/LTR copies harboring the TF-binding motifs at each position. Peaks of the motifs corresponding to HSREs are indicated by an asterisk (*) with motif names. C) An unrooted phylogenetic tree of LTR9 copies constructed using the maximum likelihood method. Fragmented and outlier copies were excluded from the analysis. In total, 1,077 (out of 2,011) of LTR9 copies were included in the tree. Representative supporting values calculated by SH-like test [68] are shown on the corresponding branches. D) Orthologous copies of LTR9 in reference genomes of other mammals. E) TFBSs on each LTR9 copy. F) TF-binding motifs on each copy at positions corresponding to HSREs. The black and gray colors respectively indicate the presence of motifs with p values of <0.0001 and <0.001. G) TFBSs on each LTR9 copy. Results of unique-read TFBSs are shown.
(TIFF)

S17 Fig Characteristics of HSREs identified in MER11C from Roadmap dataset. Results of all-read TFBSs are shown. A) Number of HERV-TFBSs mapped on each consensus position of MER11C. The X-axis indicates nucleotide position of the consensus sequence. The Y-axis indicates number of HERV/LTR copies harboring HERV-TFBSs at each position. B) Number of TF-binding motifs in HERV-TFBSs mapped on each consensus position of MER11C. The X-axis indicates nucleotide position of the consensus sequence. The Y-axis indicates number of HERV/LTR copies harboring the TF-binding motifs at each position. Peaks of the motifs corresponding to HSREs are indicated by an asterisk (*) with motif names. C) An unrooted phylogenetic tree of MER11C copies constructed using the maximum likelihood method. Fragmented and outlier copies were excluded from the analysis. In total, 748 (out of 866) of MER11C copies were included in the tree. Representative supporting values calculated by SH-like test [68] are shown on the corresponding branches. D) Orthologous copies of MER11C in reference genomes of other mammals. E) TFBSs on each MER11C copy. F) TF-binding motifs on each copy at positions corresponding to HSREs. The black and gray colors respectively indicate the presence of motifs with p values of <0.0001 and <0.001.
(TIFF)

S18 Fig Characteristics of HSREs identified in MER11B from Roadmap dataset. Results of all-read TFBSs are shown. A) Number of HERV-TFBSs mapped on each consensus position of MER11B. The X-axis indicates nucleotide position of the consensus sequence. The Y-axis indicates number of HERV/LTR copies harboring HERV-TFBSs at each position. B) Number of TF-binding motifs in HERV-TFBSs mapped on each consensus position of MER11B. The X-axis indicates nucleotide position of the consensus sequence. The Y-axis indicates number of HERV/LTR copies harboring the TF-binding motifs at each position. Peaks of the motifs corresponding to HSREs are indicated by an asterisk (*) with motif names. C) An unrooted phylogenetic tree of MER11B copies constructed using the maximum likelihood method. Fragmented and outlier copies were excluded from the analysis. In total, 377 (out of 548) of MER11B copies were included in the tree. Representative supporting values calculated by SH-like test [68] are shown on the corresponding branches. D) Orthologous copies of MER11B in reference genomes of other mammals. E) TFBSs on each MER11B copy. F) TF-binding motifs on each copy at positions corresponding to HSREs. The black and gray colors respectively indicate the presence of motifs with p values of <0.0001 and <0.001.
(TIFF)

S19 Fig A screenshot of dbHERV-REs (http://herv-tfbs.com/). The screenshot when LTR5B was selected is shown. A) Statistical and other parameters filtering HERV-TFBSs, HSREs, and HERV-DHSs. B) The list of HERV/LTRs that can be selected under the parameters. C) General information of the selected HERV/LTRs. D) Visualized data. In this figure, the graph shows number of HERV-TFBSs mapped on each consensus position.
(TIFF)

S1 Table TFs for which ChIP-Seq data was used in the present study. Bold TFs were used for ChIP-Seq by ENCODE and Roadmap.
(DOCX)

S2 Table Sequencing and analytical pipelines of ChIP-Seq used in ENCODE and roadmap. (DOCX)

S3 Table Proportions of HERV-TFBSs in the entire TFBSs in respective TFs. Results of unique-read TFBSs are shown. Top 25 TFs with respect to proportions of HERV-TFBSs are shown. TFs in which TFBSs overlapped with HERV/LTRs at least 1,000 times are shown.
(DOCX)

S4 Table Distance-based GO enrichment analysis to ascertain biological processes in which HERV-TFBSs were more enriched compared to the other TFBSs. Distance-based GO enrichment analysis using GREAT [53] algorithm was performed. Results of unique-read TFBSs are shown. TFBSs or HERV-TFBSs identified in cells treated with special conditions (e.g., supplement of interferon) were excluded. GO terms were summarized by REVIGO [73]. GO terms with hold enrichment scores >2 are shown.
(DOCX)

S5 Table Distance-based GO enrichment analysis to ascertain biological processes in which HERV/LTRs harboring TFBSs were more enriched compared to entire HERV/LTRs. Distance-based GO enrichment analysis using GREAT algorithm [53] was performed. Results of unique-read TFBSs are shown. HERV-TFBSs identified in cells treated with special conditions (e.g., supplement of interferon) were excluded. GO terms were summarized by REVIGO [73]. GO terms with hold enrichment scores >2 are shown.
(DOCX)

S6 Table Biological processes in which many types of HSREs were enriched. Results in unique-read TFBSs are shown. The GREAT enrichment analyses [53] were performed using sets of HERV-TFBSs harboring each type of HSRE in respective cell types, and then GO terms associated with many kinds of HSREs (>10) were summarized separately in cell types (up to 15 in each cell type).
(DOCX)

S7 Table Hi-C-based GO enrichment analysis to ascertain biological processes in which HERV-TFBSs were more enriched than the other TFBSs. Hi-C-based GO enrichment analysis with GREAT [53] algorithm was performed. Results of unique-read TFBSs are shown. TFBSs or HERV-TFBSs identified in cells treated with special conditions (e.g., supplement of interferon) were excluded. GO terms were summarized by REVIGO [73]. GO terms with hold enrichment scores >2 are shown.
(DOCX)

S8 Table ChIP-Seq datasets used in this study. (XLSX)

S9 Table ChIP-Seq files used in this study. (XLSX)

S10 Table DHS datasets used in this study. (XLSX)

S11 Table Genome segmentation datasets used in this study. (XLSX)

S12 Table CSHL LongPolyA RNA-Seq datasets used in this study. (XLSX)

S13 Table List of HERV/LTR types whose phylogenetic trees were reconstructed in this study. (XLSX)

## References

[1] Lander ES, Linton LM, Birren B, Nusbaum C, Zody MC, Baldwin J, et al Initial sequencing and analysis of the human genome. Nature. 2001;409: 860–921. doi: 10.1038/35057062 11237011 10.1038/35057062

[2] Hurst GD, Werren JH. The role of selfish genetic elements in eukaryotic evolution. Nat Rev Genet. 2001;2: 597–606. doi: 10.1038/35084545 11483984 10.1038/35084545

[3] Feschotte C, Gilbert C. Endogenous viruses: insights into viral evolution and impact on host biology. Nat Rev Genet. 2012;13: 283–296. doi: 10.1038/nrg3199 22421730 10.1038/nrg3199

[4] Mi S, Lee X, Li X, Veldman GM, Finnerty H, Racie L, et al Syncytin is a captive retroviral envelope protein involved in human placental morphogenesis. Nature. 2000;403: 785–789. doi: 10.1038/35001608 10693809 10.1038/35001608

[5] Blaise S, de Parseval N, Benit L, Heidmann T. Genomewide screening for fusogenic human endogenous retrovirus envelopes identifies syncytin 2, a gene conserved on primate evolution. Proc Natl Acad Sci U S A. 2003;100: 13013–13018. doi: 10.1073/pnas.2132646100 14557543 10.1073/pnas.2132646100 PMC240736

[6] Best S, Le Tissier P, Towers G, Stoye JP. Positional cloning of the mouse retrovirus restriction gene Fv1. Nature. 1996;382: 826–829. doi: 10.1038/382826a0 8752279 10.1038/382826a0

[7] Ikeda H, Laigret F, Martin MA, Repaske R. Characterization of a molecularly cloned retroviral sequence associated with Fv-4 resistance. J Virol. 1985;55: 768–777. 2991595 10.1128/jvi.55.3.768-777.1985 PMC255061

[8] Kapusta A, Kronenberg Z, Lynch VJ, Zhuo X, Ramsay L, Bourque G, et al Transposable elements are major contributors to the origin, diversification, and regulation of vertebrate long noncoding RNAs. PLoS Genet. 2013;9: e1003470 doi: 10.1371/journal.pgen.1003470 23637635 10.1371/journal.pgen.1003470 PMC3636048

[9] Jacques PE, Jeyakani J, Bourque G. The majority of primate-specific regulatory sequences are derived from transposable elements. PLoS Genet. 2013;9: e1003504 doi: 10.1371/journal.pgen.1003504 23675311 10.1371/journal.pgen.1003504 PMC3649963

[10] Ohnuki M, Tanabe K, Sutou K, Teramoto I, Sawamura Y, Narita M, et al Dynamic regulation of human endogenous retroviruses mediates factor-induced reprogramming and differentiation potential. Proc Natl Acad Sci U S A. 2014;111: 12426–12431. doi: 10.1073/pnas.1413299111 25097266 10.1073/pnas.1413299111 PMC4151758

[11] Wang J, Xie G, Singh M, Ghanbarian AT, Rasko T, Szvetnik A, et al Primate-specific endogenous retrovirus-driven transcription defines naive-like stem cells. Nature. 2014;516: 405–409. doi: 10.1038/nature13804 25317556 10.1038/nature13804

[12] Lu X, Sachs F, Ramsay L, Jacques PE, Goke J, Bourque G, et al The retrovirus HERVH is a long noncoding RNA required for human embryonic stem cell identity. Nat Struct Mol Biol. 2014;21: 423–425. doi: 10.1038/nsmb.2799 24681886 10.1038/nsmb.2799

[13] Kunarso G, Chia NY, Jeyakani J, Hwang C, Lu X, Chan YS, et al Transposable elements have rewired the core regulatory network of human embryonic stem cells. Nat Genet. 2010;42: 631–634. doi: 10.1038/ng.600 20526341 10.1038/ng.600

[14] Chuong EB, Elde NC, Feschotte C. Regulatory evolution of innate immunity through co-option of endogenous retroviruses. Science. 2016;351: 1083–1087. doi: 10.1126/science.aad5497 26941318 10.1126/science.aad5497 PMC4887275

[15] Durruthy-Durruthy J, Sebastiano V, Wossidlo M, Cepeda D, Cui J, Grow EJ, et al The primate-specific noncoding RNA HPAT5 regulates pluripotency during human preimplantation development and nuclear reprogramming. Nat Genet. 2016;48: 44–52. doi: 10.1038/ng.3449 26595768 10.1038/ng.3449 PMC4827613

[16] Becker KG, Swergold GD, Ozato K, Thayer RE. Binding of the ubiquitous nuclear transcription factor YY1 to a cis regulatory sequence in the human LINE-1 transposable element. Hum Mol Genet. 1993;2: 1697–1702. 8268924 10.1093/hmg/2.10.1697

[17] Minakami R, Kurose K, Etoh K, Furuhata Y, Hattori M, Sakaki Y. Identification of an internal cis-element essential for the human L1 transcription and a nuclear factor(s) binding to the element. Nucleic Acids Res. 1992;20: 3139–3145. 1320255 10.1093/nar/20.12.3139 PMC312450

[18] Tchenio T, Casella JF, Heidmann T. Members of the SRY family regulate the human LINE retrotransposons. Nucleic Acids Res. 2000;28: 411–415. 10606637 10.1093/nar/28.2.411 PMC102531

[19] Mathias SL, Scott AF. Promoter binding proteins of an active human L1 retrotransposon. Biochem Biophys Res Commun. 1993;191: 625–632. doi: 10.1006/bbrc.1993.1263 8384847 10.1006/bbrc.1993.1263

[20] Fuchs NV, Kraft M, Tondera C, Hanschmann KM, Lower J, Lower R. Expression of the human endogenous retrovirus (HERV) group HML-2/HERV-K does not depend on canonical promoter elements but is regulated by transcription factors Sp1 and Sp3. J Virol. 2011;85: 3436–3448. doi: 10.1128/JVI.02539-10 21248046 10.1128/JVI.02539-10 PMC3067833

[21] Grow EJ, Flynn RA, Chavez SL, Bayless NL, Wossidlo M, Wesche DJ, et al Intrinsic retroviral reactivation in human preimplantation embryos and pluripotent cells. Nature. 2015;522: 221–225. doi: 10.1038/nature14308 25896322 10.1038/nature14308 PMC4503379

[22] Manghera M, Douville RN. Endogenous retrovirus-K promoter: a landing strip for inflammatory transcription factors? Retrovirology. 2013;10: 16 doi: 10.1186/1742-4690-10-16 23394165 10.1186/1742-4690-10-16 PMC3598470

[23] Sjottem E, Anderssen S, Johansen T. The promoter activity of long terminal repeats of the HERV-H family of human retrovirus-like elements is critically dependent on Sp1 family proteins interacting with a GC/GT box located immediately 3' to the TATA box. J Virol. 1996;70: 188–198. 8523525 10.1128/jvi.70.1.188-198.1996 PMC189804

[24] Yu X, Zhu X, Pi W, Ling J, Ko L, Takeda Y, et al The long terminal repeat (LTR) of ERV-9 human endogenous retrovirus binds to NF-Y in the assembly of an active LTR enhancer complex NF-Y/MZF1/GATA-2. J Biol Chem. 2005;280: 35184–35194. doi: 10.1074/jbc.M508138200 16105833 10.1074/jbc.M508138200

[25] Gerlo S, Davis JR, Mager DL, Kooijman R. Prolactin in man: a tale of two promoters. Bioessays. 2006;28: 1051–1055. doi: 10.1002/bies.20468 16998840 10.1002/bies.20468 PMC1891148

[26] Jordan IK, Rogozin IB, Glazko GV, Koonin EV. Origin of a substantial fraction of human regulatory sequences from transposable elements. Trends Genet. 2003;19: 68–72. 12547512 10.1016/s0168-9525(02)00006-9

[27] van de Lagemaat LN, Landry JR, Mager DL, Medstrand P. Transposable elements in mammals promote regulatory variation and diversification of genes with specialized functions. Trends Genet. 2003;19: 530–536. doi: 10.1016/j.tig.2003.08.004 14550626 10.1016/j.tig.2003.08.004

[28] Bejerano G, Lowe CB, Ahituv N, King B, Siepel A, Salama SR, et al A distal enhancer and an ultraconserved exon are derived from a novel retroposon. Nature. 2006;441: 87–90. doi: 10.1038/nature04696 16625209 10.1038/nature04696

[29] Pi W, Zhu X, Wu M, Wang Y, Fulzele S, Eroglu A, et al Long-range function of an intergenic retrotransposon. Proc Natl Acad Sci U S A. 2010;107: 12992–12997. doi: 10.1073/pnas.1004139107 20615953 10.1073/pnas.1004139107 PMC2919959

[30] Suntsova M, Gogvadze EV, Salozhin S, Gaifullin N, Eroshkin F, Dmitriev SE, et al Human-specific endogenous retroviral insert serves as an enhancer for the schizophrenia-linked gene PRODH. Proc Natl Acad Sci U S A. 2013;110: 19472–19477. doi: 10.1073/pnas.1318172110 24218577 10.1073/pnas.1318172110 PMC3845128

[31] Chuong EB, Rumi MA, Soares MJ, Baker JC. Endogenous retroviruses function as species-specific enhancer elements in the placenta. Nat Genet. 2013;45: 325–329. doi: 10.1038/ng.2553 23396136 10.1038/ng.2553 PMC3789077

[32] Roman AC, Gonzalez-Rico FJ, Molto E, Hernando H, Neto A, Vicente-Garcia C, et al Dioxin receptor and SLUG transcription factors regulate the insulator activity of B1 SINE retrotransposons via an RNA polymerase switch. Genome Res. 2011;21: 422–432. doi: 10.1101/gr.111203.110 21324874 10.1101/gr.111203.110 PMC3044856

[33] Wang J, Vicente-Garcia C, Seruggia D, Molto E, Fernandez-Minan A, Neto A, et al MIR retrotransposon sequences provide insulators to the human genome. Proc Natl Acad Sci U S A. 2015;112: E4428–4437. doi: 10.1073/pnas.1507253112 26216945 10.1073/pnas.1507253112 PMC4538669

[34] Chuong EB, Elde NC, Feschotte C. Regulatory activities of transposable elements: from conflicts to benefits. Nat Rev Genet. 2017;18: 71–86. doi: 10.1038/nrg.2016.139 27867194 10.1038/nrg.2016.139 PMC5498291

[35] Feschotte C. Transposable elements and the evolution of regulatory networks. Nat Rev Genet. 2008;9: 397–405. doi: 10.1038/nrg2337 18368054 10.1038/nrg2337 PMC2596197

[36] Lynch VJ, Nnamani MC, Kapusta A, Brayer K, Plaza SL, Mazur EC, et al Ancient transposable elements transformed the uterine regulatory landscape and transcriptome during the evolution of mammalian pregnancy. Cell Rep. 2015;10: 551–561. doi: 10.1016/j.celrep.2014.12.052 25640180 10.1016/j.celrep.2014.12.052 PMC4447085

[37] Sundaram V, Cheng Y, Ma Z, Li D, Xing X, Edge P, et al Widespread contribution of transposable elements to the innovation of gene regulatory networks. Genome Res. 2014;24: 1963–1976. doi: 10.1101/gr.168872.113 25319995 10.1101/gr.168872.113 PMC4248313

[38] Coffin JM, Hughes SH, Varmus HE, editors. Retroviruses. Cold Spring Harbor (NY): Cold Spring Harbor Laboratory Press; 1997. 21433340

[39] Koyanagi-Aoi M, Ohnuki M, Takahashi K, Okita K, Noma H, Sawamura Y, et al Differentiation-defective phenotypes revealed by large-scale analyses of human pluripotent stem cells. Proc Natl Acad Sci U S A. 2013;110: 20569–20574. doi: 10.1073/pnas.1319061110 24259714 10.1073/pnas.1319061110 PMC3870695

[40] ENCODE Project Consortium. An integrated encyclopedia of DNA elements in the human genome. Nature. 2012;489: 57–74. doi: 10.1038/nature11247 22955616 10.1038/nature11247 PMC3439153

[41] Tsankov AM, Gu H, Akopian V, Ziller MJ, Donaghey J, Amit I, et al Transcription factor binding dynamics during human ES cell differentiation. Nature. 2015;518: 344–349. doi: 10.1038/nature14233 25693565 10.1038/nature14233 PMC4499331

[42] Jin Y, Tam OH, Paniagua E, Hammell M. TEtranscripts: a package for including transposable elements in differential expression analysis of RNA-seq datasets. Bioinformatics. 2015;31: 3593–3599. doi: 10.1093/bioinformatics/btv422 26206304 10.1093/bioinformatics/btv422 PMC4757950

[43] Goode DK, Obier N, Vijayabaskar MS, Lie ALM, Lilly AJ, Hannah R, et al Dynamic Gene Regulatory Networks Drive Hematopoietic Specification and Differentiation. Dev Cell. 2016;36: 572–587. doi: 10.1016/j.devcel.2016.01.024 26923725 10.1016/j.devcel.2016.01.024 PMC4780867

[44] Wang J, Zhuang J, Iyer S, Lin X, Whitfield TW, Greven MC, et al Sequence features and chromatin structure around the genomic regions bound by 119 human transcription factors. Genome Res. 2012;22: 1798–1812. doi: 10.1101/gr.139105.112 22955990 10.1101/gr.139105.112 PMC3431495

[45] Dekker J, Marti-Renom MA, Mirny LA. Exploring the three-dimensional organization of genomes: interpreting chromatin interaction data. Nat Rev Genet. 2013;14: 390–403. doi: 10.1038/nrg3454 23657480 10.1038/nrg3454 PMC3874835

[46] Cohen CJ, Lock WM, Mager DL. Endogenous retroviral LTRs as promoters for human genes: a critical assessment. Gene. 2009;448: 105–114. doi: 10.1016/j.gene.2009.06.020 19577618 10.1016/j.gene.2009.06.020

[47] Ernst J, Kellis M. ChromHMM: automating chromatin-state discovery and characterization. Nat Methods. 2012;9: 215–216. doi: 10.1038/nmeth.1906 22373907 10.1038/nmeth.1906 PMC3577932

[48] Hoffman MM, Buske OJ, Wang J, Weng Z, Bilmes JA, Noble WS. Unsupervised pattern discovery in human chromatin structure through genomic segmentation. Nat Methods. 2012;9: 473–476. doi: 10.1038/nmeth.1937 22426492 10.1038/nmeth.1937 PMC3340533

[49] Hoffman MM, Ernst J, Wilder SP, Kundaje A, Harris RS, Libbrecht M, et al Integrative annotation of chromatin elements from ENCODE data. Nucleic Acids Res. 2013;41: 827–841. doi: 10.1093/nar/gks1284 23221638 10.1093/nar/gks1284 PMC3553955

[50] Leung D, Jung I, Rajagopal N, Schmitt A, Selvaraj S, Lee AY, et al Integrative analysis of haplotype-resolved epigenomes across human tissues. Nature. 2015;518: 350–354. doi: 10.1038/nature14217 25693566 10.1038/nature14217 PMC4449149

[51] Subramanian RP, Wildschutte JH, Russo C, Coffin JM. Identification, characterization, and comparative genomic distribution of the HERV-K (HML-2) group of human endogenous retroviruses. Retrovirology. 2011;8: 90 doi: 10.1186/1742-4690-8-90 22067224 10.1186/1742-4690-8-90 PMC3228705

[52] Fleming JD, Pavesi G, Benatti P, Imbriano C, Mantovani R, Struhl K. NF-Y coassociates with FOS at promoters, enhancers, repetitive elements, and inactive chromatin regions, and is stereo-positioned with growth-controlling transcription factors. Genome Res. 2013;23: 1195–1209. doi: 10.1101/gr.148080.112 23595228 10.1101/gr.148080.112 PMC3730095

[53] McLean CY, Bristor D, Hiller M, Clarke SL, Schaar BT, Lowe CB, et al GREAT improves functional interpretation of cis-regulatory regions. Nat Biotechnol. 2010;28: 495–501. doi: 10.1038/nbt.1630 20436461 10.1038/nbt.1630 PMC4840234

[54] Mifsud B, Tavares-Cadete F, Young AN, Sugar R, Schoenfelder S, Ferreira L, et al Mapping long-range promoter contacts in human cells with high-resolution capture Hi-C. Nat Genet. 2015;47: 598–606. doi: 10.1038/ng.3286 25938943 10.1038/ng.3286

[55] Cairns J, Freire-Pritchett P, Wingett SW, Varnai C, Dimond A, Plagnol V, et al CHiCAGO: robust detection of DNA looping interactions in Capture Hi-C data. Genome Biol. 2016;17: 127 doi: 10.1186/s13059-016-0992-2 27306882 10.1186/s13059-016-0992-2 PMC4908757

[56] Goke J, Lu X, Chan YS, Ng HH, Ly LH, Sachs F, et al Dynamic transcription of distinct classes of endogenous retroviral elements marks specific populations of early human embryonic cells. Cell Stem Cell. 2015;16: 135–141. doi: 10.1016/j.stem.2015.01.005 25658370 10.1016/j.stem.2015.01.005

[57] Satou Y, Miyazato P, Ishihara K, Yaguchi H, Melamed A, Miura M, et al The retrovirus HTLV-1 inserts an ectopic CTCF-binding site into the human genome. Proc Natl Acad Sci U S A. 2016. 10.1073/pnas.1423199113 PMC4801255 26929370

[58] Collins PL, Kyle KE, Egawa T, Shinkai Y, Oltz EM. The histone methyltransferase SETDB1 represses endogenous and exogenous retroviruses in B lymphocytes. Proc Natl Acad Sci U S A. 2015;112: 8367–8372. doi: 10.1073/pnas.1422187112 26100872 10.1073/pnas.1422187112 PMC4500218

[59] Kuse K, Ito J, Miyake A, Kawasaki J, Watanabe S, Makundi I, et al Existence of Two Distinct Infectious Endogenous Retroviruses in Domestic Cats and Their Different Strategies for Adaptation to Transcriptional Regulation. J Virol. 2016;90: 9029–9045. doi: 10.1128/JVI.00716-16 27466428 10.1128/JVI.00716-16 PMC5044828

[60] Izsvak Z, Wang J, Singh M, Mager DL, Hurst LD. Pluripotency and the endogenous retrovirus HERVH: Conflict or serendipity? Bioessays. 2016;38: 109–117. doi: 10.1002/bies.201500096 26735931 10.1002/bies.201500096

[61] Li H, Handsaker B, Wysoker A, Fennell T, Ruan J, Homer N, et al The Sequence Alignment/Map format and SAMtools. Bioinformatics. 2009;25: 2078–2079. doi: 10.1093/bioinformatics/btp352 19505943 10.1093/bioinformatics/btp352 PMC2723002

[62] Quinlan AR. BEDTools: The Swiss-Army Tool for Genome Feature Analysis. Curr Protoc Bioinformatics. 2014;47: 11.12.11–34. 10.1002/0471250953.bi1112s47 PMC4213956 25199790

[63] Katoh K, Standley DM. MAFFT multiple sequence alignment software version 7: improvements in performance and usability. Mol Biol Evol. 2013;30: 772–780. doi: 10.1093/molbev/mst010 23329690 10.1093/molbev/mst010 PMC3603318

[64] Grant CE, Bailey TL, Noble WS. FIMO: scanning for occurrences of a given motif. Bioinformatics. 2011;27: 1017–1018. doi: 10.1093/bioinformatics/btr064 21330290 10.1093/bioinformatics/btr064 PMC3065696

[65] Mathelier A, Fornes O, Arenillas DJ, Chen CY, Denay G, Lee J, et al JASPAR 2016: a major expansion and update of the open-access database of transcription factor binding profiles. Nucleic Acids Res. 2016;44: D110–115. doi: 10.1093/nar/gkv1176 26531826 10.1093/nar/gkv1176 PMC4702842

[66] Kulakovskiy IV, Vorontsov IE, Yevshin IS, Soboleva AV, Kasianov AS, Ashoor H, et al HOCOMOCO: expansion and enhancement of the collection of transcription factor binding sites models. Nucleic Acids Res. 2016;44: D116–125. doi: 10.1093/nar/gkv1249 26586801 10.1093/nar/gkv1249 PMC4702883

[67] Stamatakis A. RAxML version 8: a tool for phylogenetic analysis and post-analysis of large phylogenies. Bioinformatics. 2014;30: 1312–1313. doi: 10.1093/bioinformatics/btu033 24451623 10.1093/bioinformatics/btu033 PMC3998144

[68] Guindon S, Dufayard JF, Lefort V, Anisimova M, Hordijk W, Gascuel O. New algorithms and methods to estimate maximum-likelihood phylogenies: assessing the performance of PhyML 3.0. Syst Biol. 2010;59: 307–321. doi: 10.1093/sysbio/syq010 20525638 10.1093/sysbio/syq010

[69] Johnson WE, Coffin JM. Constructing primate phylogenies from ancient retrovirus sequences. Proc Natl Acad Sci U S A. 1999;96: 10254–10260. 10468595 10.1073/pnas.96.18.10254 PMC17875

[70] Myers EW, Miller W. Optimal alignments in linear space. Comput Appl Biosci. 1988;4: 11–17. 3382986 10.1093/bioinformatics/4.1.11

[71] Magiorkinis G, Blanco-Melo D, Belshaw R. The decline of human endogenous retroviruses: extinction and survival. Retrovirology. 2015;12: 8 doi: 10.1186/s12977-015-0136-x 25640971 10.1186/s12977-015-0136-x PMC4335370

[72] Hedges SB, Marin J, Suleski M, Paymer M, Kumar S. Tree of life reveals clock-like speciation and diversification. Mol Biol Evol. 2015;32: 835–845. doi: 10.1093/molbev/msv037 25739733 10.1093/molbev/msv037 PMC4379413

[73] Supek F, Bosnjak M, Skunca N, Smuc T. REVIGO summarizes and visualizes long lists of gene ontology terms. PLoS One. 2011;6: e21800 doi: 10.1371/journal.pone.0021800 21789182 10.1371/journal.pone.0021800 PMC3138752

[74] Derrien T, Estelle J, Marco Sola S, Knowles DG, Raineri E, Guigo R, et al Fast computation and applications of genome mappability. PLoS One. 2012;7: e30377 doi: 10.1371/journal.pone.0030377 22276185 10.1371/journal.pone.0030377 PMC3261895

