# Supplementary figures and images for: Systematic identification and characterization of regulatory elements derived from human endogenous retroviruses
Source: PLoS Genet. 2017 Jul 12;13(7):e1006883. doi: 10.1371/journal.pgen.1006883 (PMC5529029; doi:10.1371/journal.pgen.1006883)

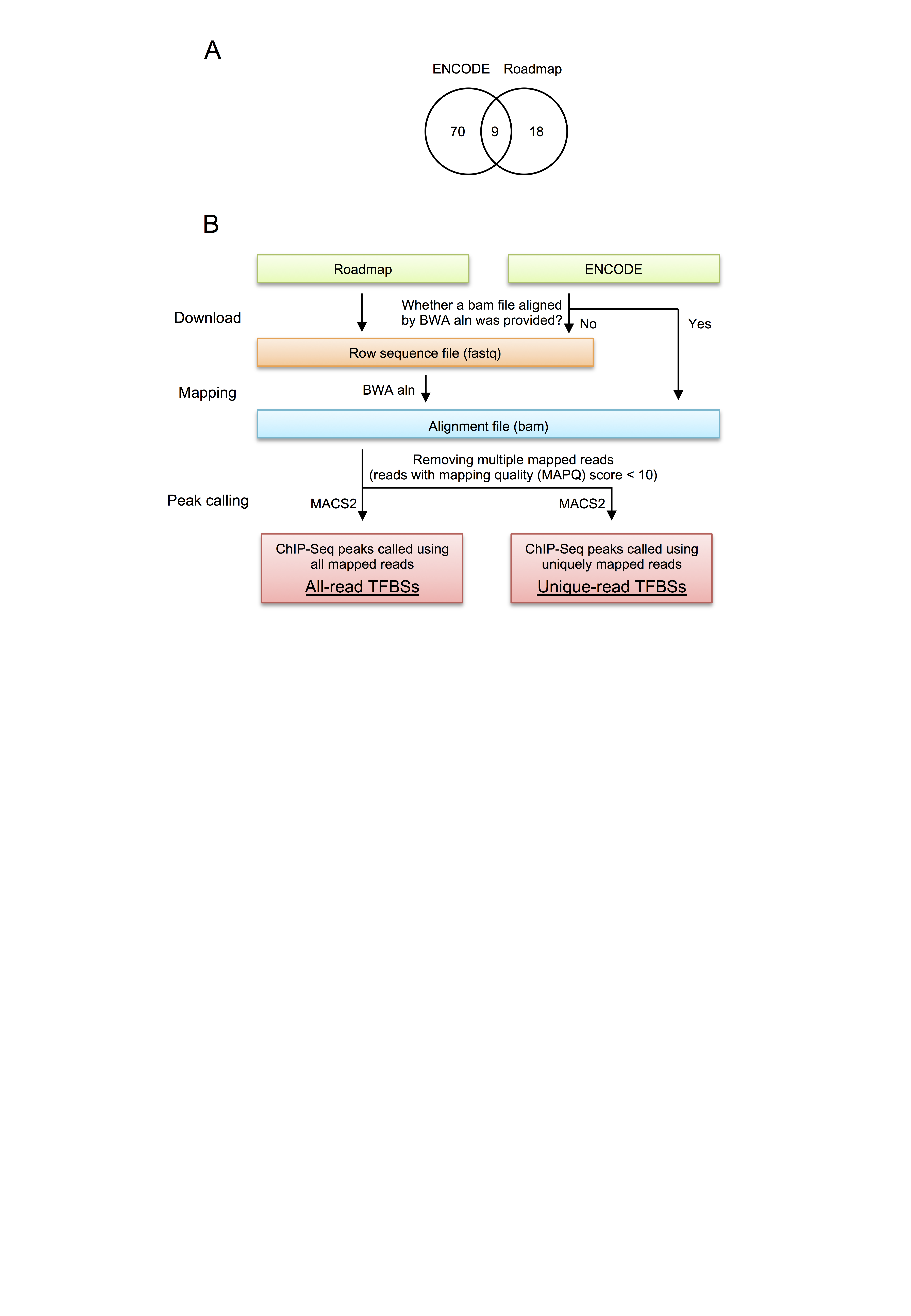

Supplement: S1 Fig — A). TFs for which ChIP-Seq was performed in this study. ChIP-Seq data for MYC, CTCF, FOXA1, FOXA2, HNF4A, NANOG, POU5F1, PRDM1, and SP1 were provided by ENCODE and Roadmap. ChIP-Seq data for other TFs were provided by either ENCODE or Roadmap. Detailed information is summarized in S1 Table. B) An analytical pipeline for peak calling of ChIP-Seq. We generated two types of TFBS datasets: all- and unique-read TFBSs. All-read TFBSs are ChIP-Seq peaks called with all reads mapped to the reference human genome. Unique-read TFBSs are ChIP-Seq peaks called with only reads that were uniquely mapped to the reference human genome. (TIFF) [file pgen.1006883.s001.tiff]

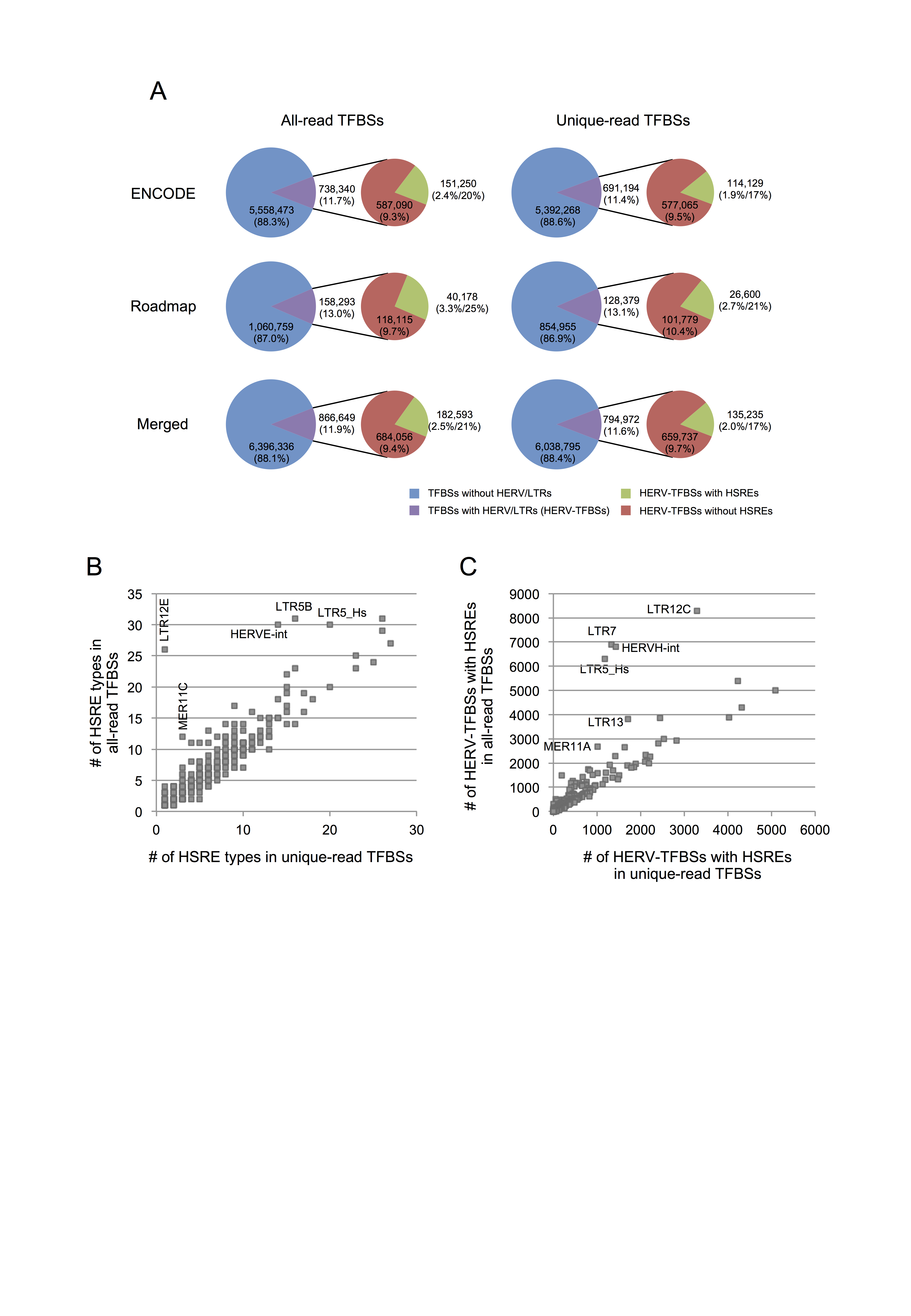

Supplement: S2 Fig — A) Proportions of HERV-TFBSs and HERV-TFBSs with HSREs. The left and right panels show results of all- and unique-read TFBSs, respectively. Proportions of HERV-TFBSs harboring HSREs in entire TFBSs (left value) and in HERV-TFBSs (right value) are shown. In the “merged” dataset, TFBSs of the same TF were merged between ENCODE and Roadmap, and were then counted. B) Comparison between the numbers of HSRE types identified from all- and unique-read TFBSs. A dot indicates a HERV/LTR type. C) Comparison between the numbers of HERV-TFBSs harboring HSREs from all- and unique-read TFBSs. A dot indicates a HERV/LTR type. (TIFF) [file pgen.1006883.s002.tiff]

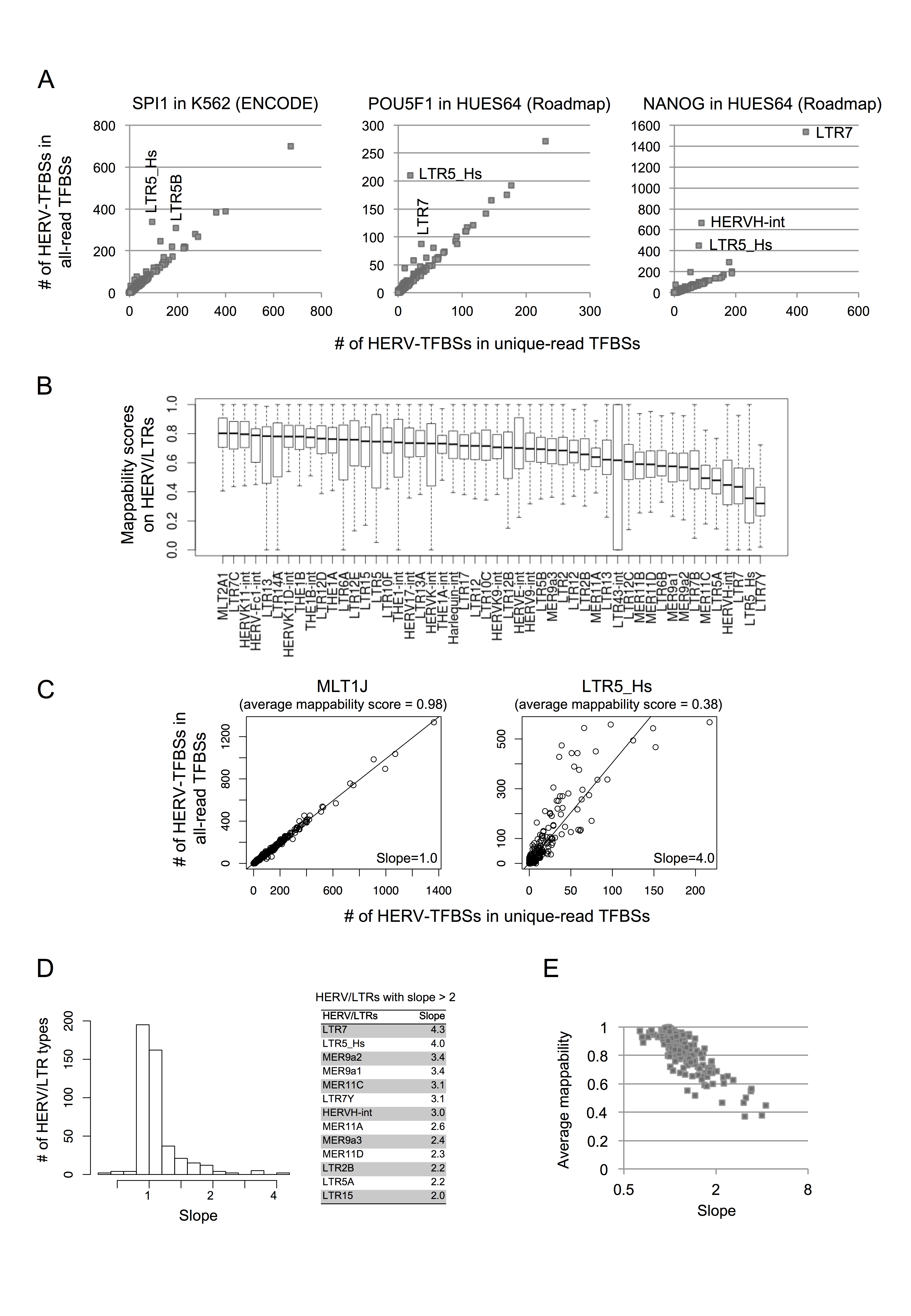

Supplement: S3 Fig — A) Comparison between the numbers of HERV-TFBSs of all- and unique-read TFBSs. The comparison was performed in respective ChIP-Seq experiments, and the results for SPI1 in K562 cells from the ENCODE dataset, POU5F1 in HUES64 cells from the Roadmap dataset, and NANOG in HUES64 cells from the Roadmap dataset are shown. In all the three ChIP-Seq experiments, 36-bp single-end sequencing was performed. A dot indicates a HERV/LTR type. In most HERV/LTRs, numbers of HERV-TFBSs were approximately the same. However, in some HERV/LTRs such as LTR5_Hs and LTR7, numbers of HERV-TFBSs was higher for all-read TFBSs than for unique-read TFBSs. B) Distribution of genomic mappability (uniqueness) scores on HERV/LTR sequences. Scores are normalized between 0 and 1, with 1 representing a unique sequence and 0 representing a sequence that occurs more than 4 times in the genome (see http://genome.ucsc.edu/). Mappability score of 36-bp single-end sequencing was calculated with gem-mappability [74]. Average mappability scores of HERV/LTR copies were calculated, and the distribution was shown separately in respective HERV/LTR types. With respect to median value of the mappability score, the worst 50 of HERV/LTR types are shown. C) Comparison between the numbers of HERV-TFBSs of all- and unique-read TFBSs. The comparison was performed in respective HERV/LTR types. Results for MLT1J and LTR5_Hs are shown. A dot indicates a ChIP-Seq experiment. Linear regression was performed, and the slope was indicated. In MLT1J with high genomic mappability (average score = 0.98), numbers of HERV-TFBSs in respective ChIP-Seq experiments are approximately the same for all- and unique-read TFBSs (slope = 1.0). In LTR5_Hs with low genomic mappability (average score = 0.38), numbers of HERV-TFBSs in respective ChIP-Seq experiments tended to be approximately four times higher for all-read TFBSs than for unique-read TFBSs (slope = 4.0). D) Distribution of slopes of linear regressions (mentioned in (C)) in respec [file pgen.1006883.s003.tiff]

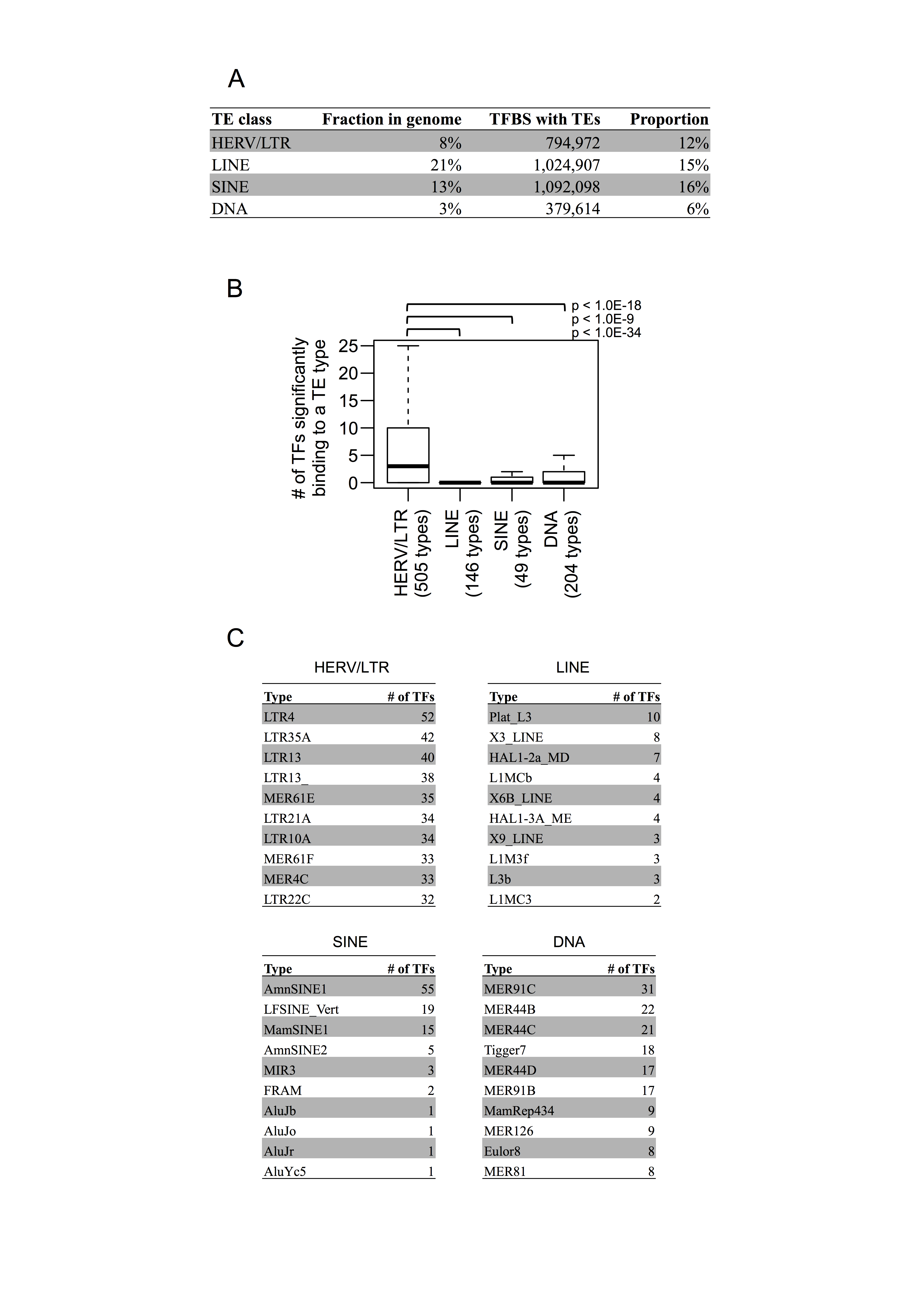

Supplement: S4 Fig — Results of unique-read TFBSs are shown. A) Number of TFBSs overlapping with respective TE classes. B) Distribution of the number of TFs significantly binding to respective TE types. Out of 106 TFs (79 ENCODE TFs + 27 Roadmap TFs), the number of TFs that are significantly bounded to a TE type was counted. The distribution is separately shown in respective TE classes. Outliers of TE types are not shown. Enrichment significance values were measured using a randomization test shuffling genomic position of TFBSs. TFs with z score >5 and fold enrichment score >2 were considered as significantly binding to the TE type. To statistically compare HERV/LTR with other TEs with respect to the numbers of TFs, Mann-Whitney U test was performed. C) TE types bounded by many TFs. (TIFF) [file pgen.1006883.s004.tiff]

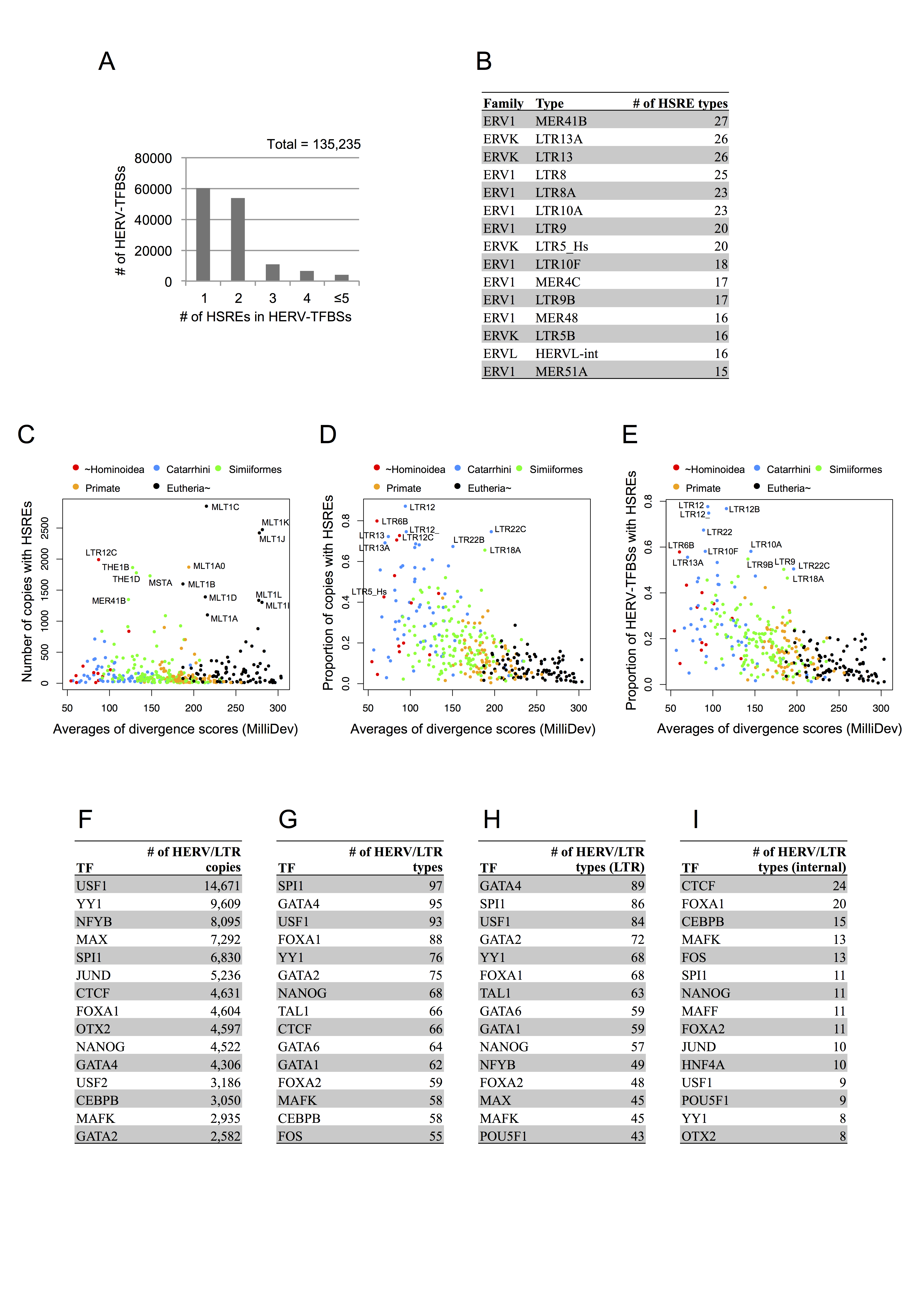

Supplement: S5 Fig — Results of unique-read TFBSs are shown. A) Distribution of HSREs present in HERV-TFBSs. The Y-axis indicates the number of HERV-TFBSs containing 1, 2, 3, 4, and greater than or equal to 5 HSREs. B) HERV/LTRs that contained many types of HSREs (TFs). C) and D) average divergence of each HERV/LTR type from the consensus sequence and absolute numbers (C) or proportions (D) of copies containing HSREs. Color of a dot indicates insertion period of the HERV/LTR type judged by distribution of orthologous copies in the mammalian genome. E) average divergence of each HERV/LTR type from the consensus sequence and proportions of HERV-TFBSs containing HSREs. Please note the difference in Y-axis between (D) and (E). F) HSREs (TFs) observed in many HERV/LTR copies. G) HSREs (TFs) observed in many types of HERV/LTRs. H) and I) HSREs (TFs) observed in many types of HERV/LTRs classified into LTR (H) and internal sequence (I). (TIFF) [file pgen.1006883.s005.tiff]

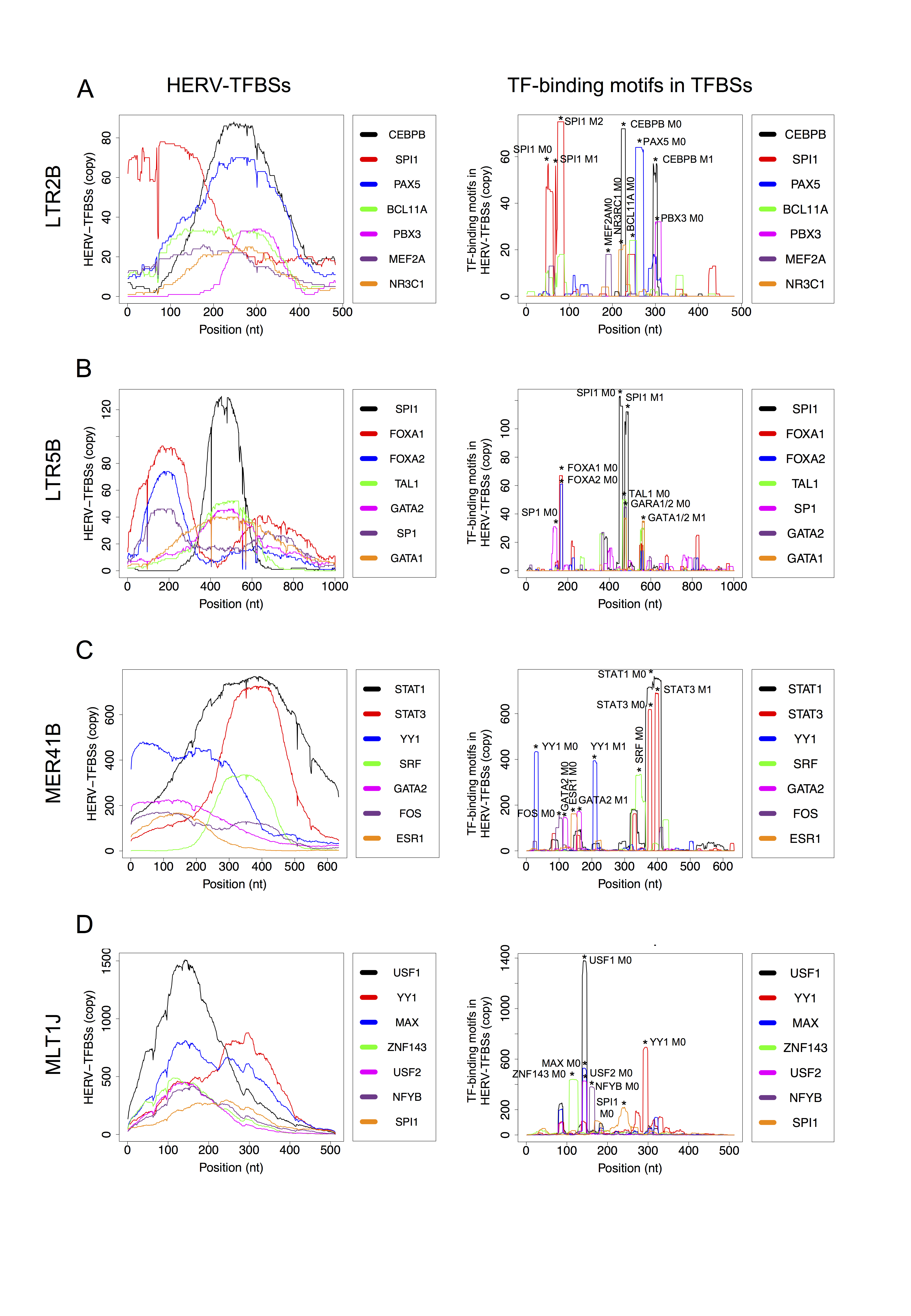

Supplement: S6 Fig — Left panel: number of HERV-TFBSs mapped on each consensus position of LTR2B (A), LTR5B (B), MER41B (C), and MLI1J (D). The X-axis indicates the nucleotide position on the consensus sequence of the corresponding HERV/LTR type. The Y-axis indicates the number of HERV/LTR copies harboring HERV-TFBSs at each position. Right panel: number of TF-binding motifs in HERV-TFBSs mapped on each consensus position of LTR2B (A), LTR5B (B), MER41B (C), and MLI1J (D). The X-axis indicates the nucleotide position of the consensus sequence. The Y-axis indicates the number of HERV/LTR copies harboring the TF-binding motifs at each position. Peaks of the motifs corresponding to HSREs are indicated with an asterisk (*) with motif names. (TIFF) [file pgen.1006883.s006.tiff]

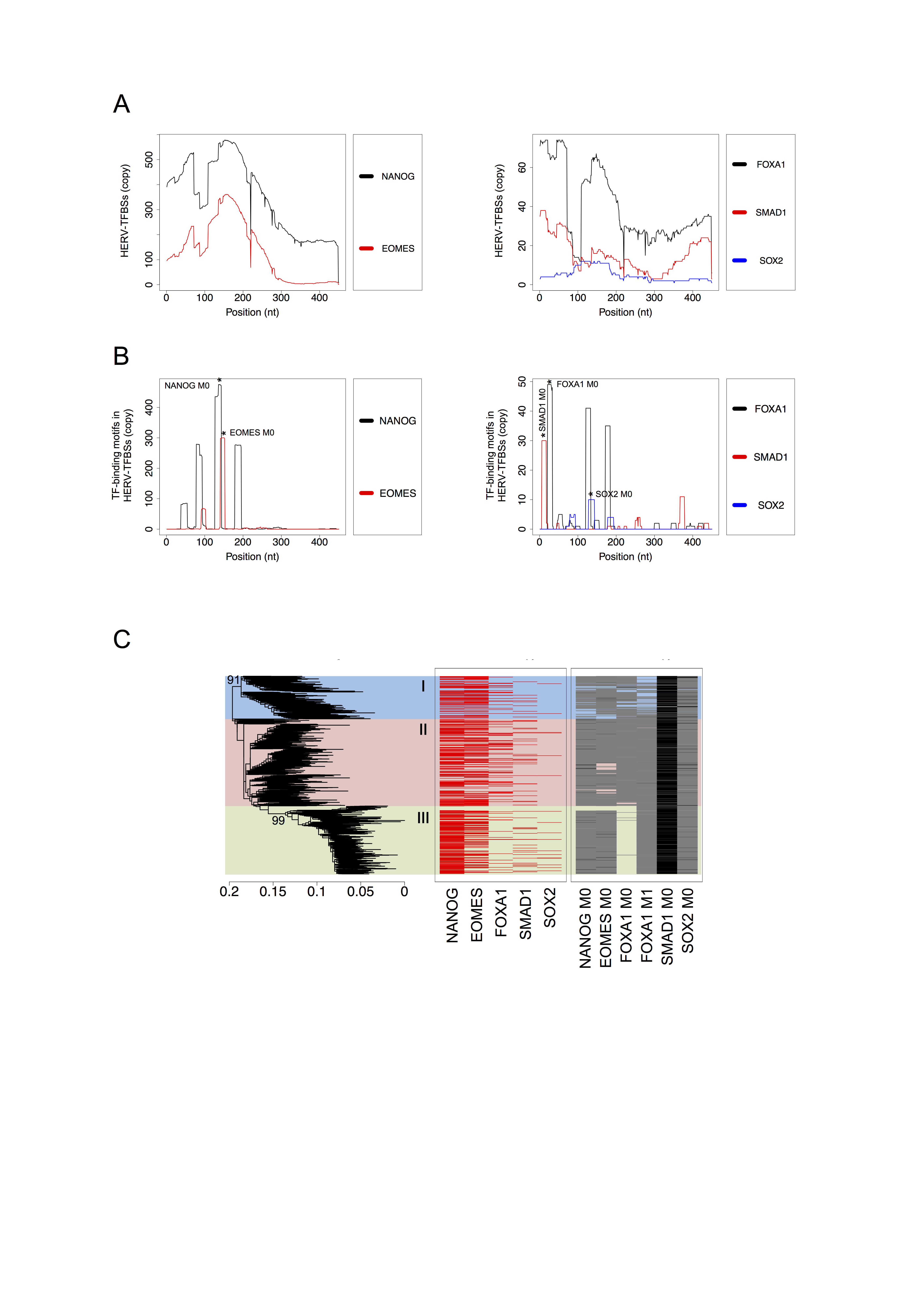

Supplement: S7 Fig — A) Number of HERV-TFBSs mapped on each consensus position of LTR7. Results of NANOG and EOMES are shown in the left panel, and those of FOXA1, SMAD1, and SOX2 are shown in the right panel. The X-axis indicates nucleotide position of the consensus sequence of LTR7. The Y-axis indicates the number of HERV/LTR copies harboring HERV-TFBSs at each position. B) Number of TF-binding motifs in HERV-TFBSs mapped on each consensus position of LTR7. Results of NANOG and EOMES are shown in the left panel, and those of FOXA1, SMAD1, and SOX2 are shown in the right panel. The X-axis indicates a consensus position of LTR7. The Y-axis indicates the number of HERV/LTR copies harboring the TF-binding motifs in TFBSs at each position. Peaks of the motifs corresponding to HSREs are indicated by an asterisk (*) with motif names (e.g., SOX2 M0). C) Left, phylogenetic tree of LTR7 copies as seen in Fig 3G. Middle, TFBSs on each LTR7 copy. The order of LTR7 copies is the same to the left tree. Right, TF-binding motifs at positions corresponding to HSREs on each LTR7 copy. The order of LTR7 copies is the same to the left tree. Black and gray colors respectively indicate the presence of motifs with p values of <0.0001 and <0.001. (TIFF) [file pgen.1006883.s007.tiff]

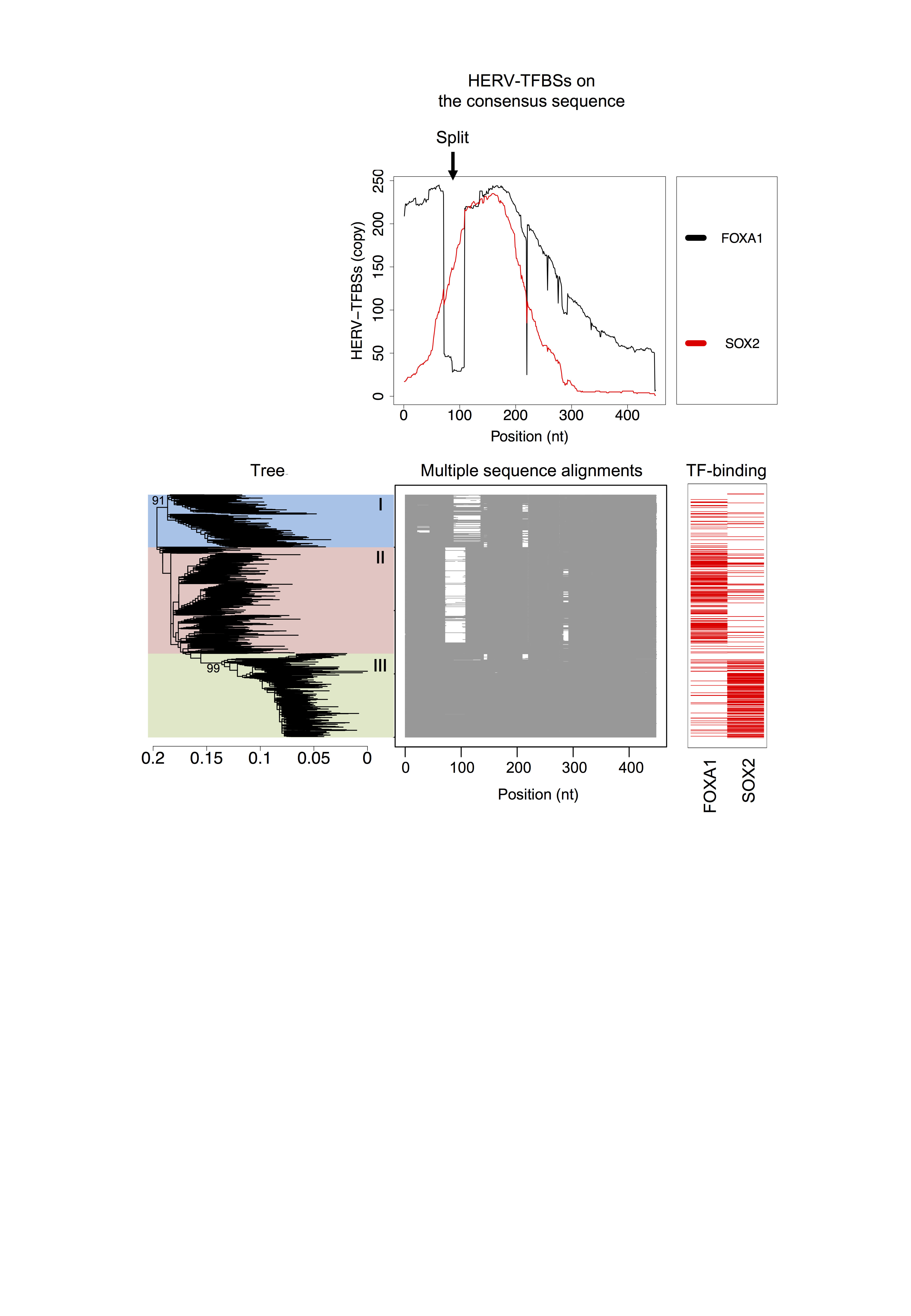

Supplement: S8 Fig — Top, number of HERV-TFBSs mapped on each consensus position of LTR7. The split of HERV-TFBS peaks is indicated by an arrow. Bottom left, phylogenetic tree of LTR7. Bottom right, MSA of LTR7. Order of the LTR7 copies is the same to the left tree. A deletion introducing the split was observed in sequences of subgroup II. FOXA1 peak was especially affected by the deletion because FOXA1 disproportionately bound to subgroup II. (TIFF) [file pgen.1006883.s008.tiff]

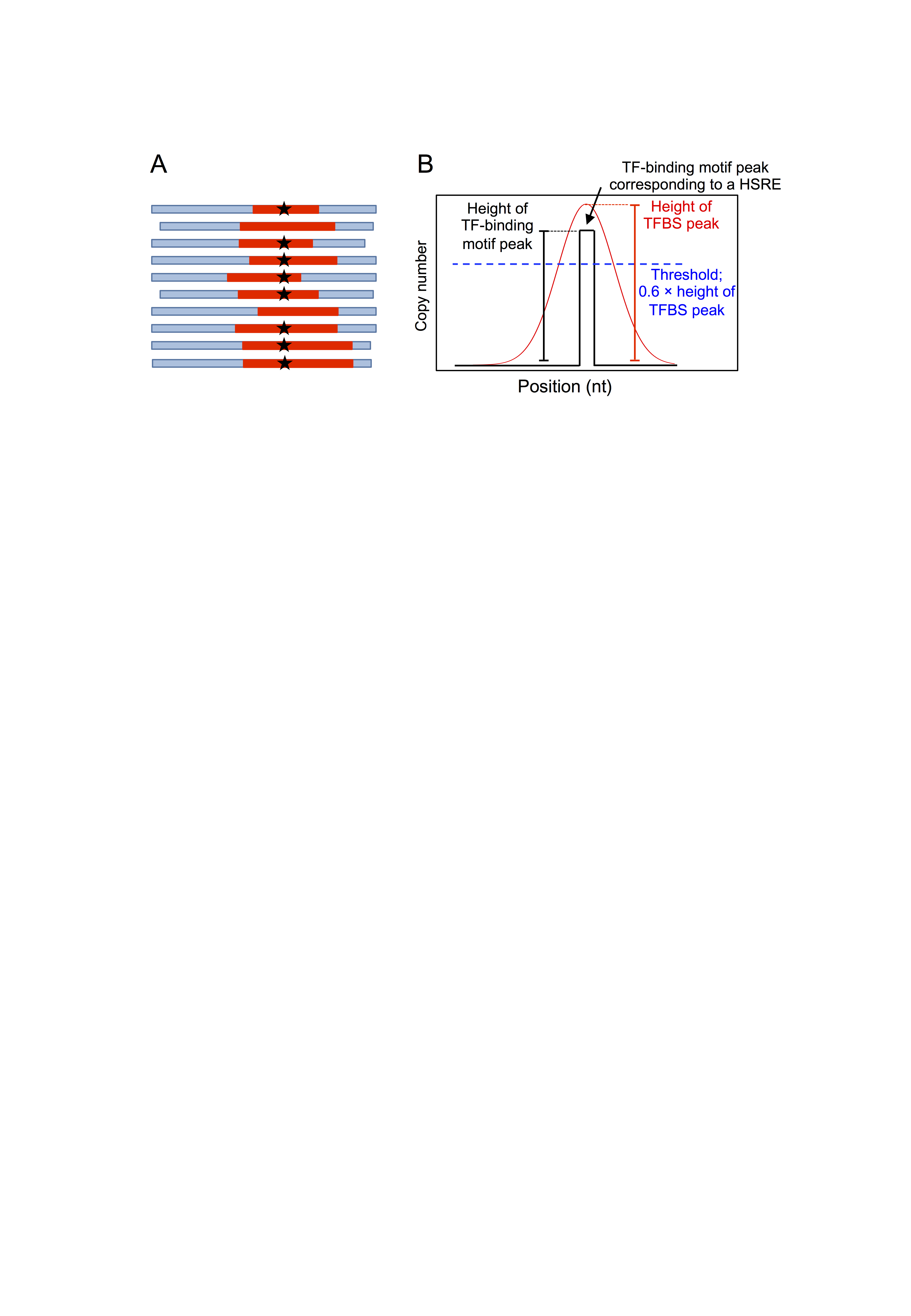

Supplement: S9 Fig — A) MSA of HERV/LTR copies (blue) harboring HERV-TFBSs (red). TF-binding motifs in HERV-TFBSs are indicated as star marks. B) Number of HERV-TFBSs (red) and TF-binding motifs (black) mapped on each consensus position of the HERV/LTRs. To identify HSREs, peak heights are compared between HERV-TFBSs and TF-binding motifs. If the height of the TF-binding motif peak is greater than 60% of the height of the HERV-TFBS peak, we regard the set of TF-binding motifs as HSRE. (TIFF) [file pgen.1006883.s009.tiff]

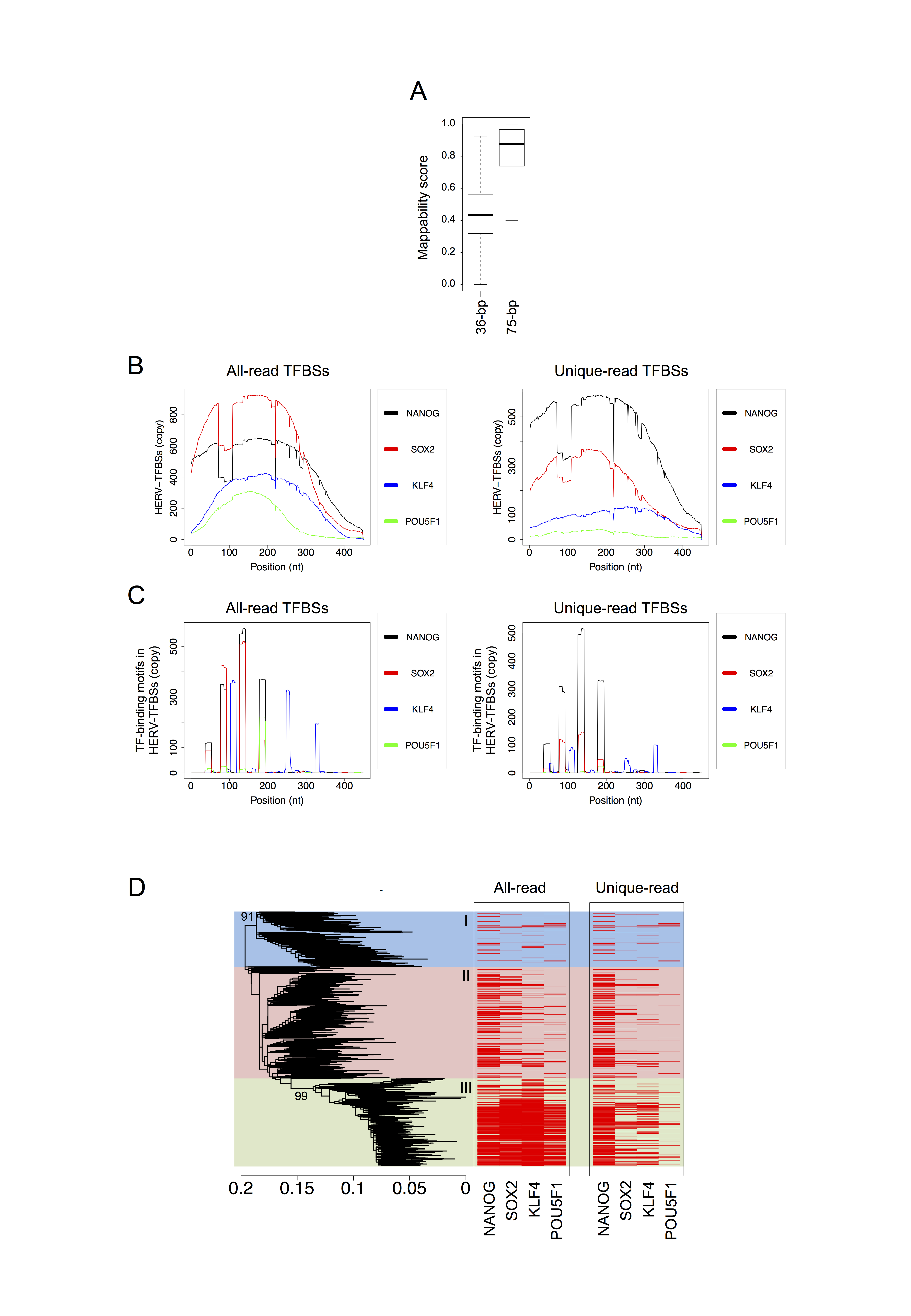

Supplement: S10 Fig — ChIP-Seq data on SOX2, KLF4, and POU5F1 (75-bp single-end) was provided by Ohnuki et al. [10]. ChIP-Seq data on NANOG (100-bp paired-end) was provided by Durruthy-Durruthy et al. [15]. A) Comparison between genomic mappability scores of LTR7 for 36-bp and 75-bp sequencing. B) Number of HERV-TFBSs mapped on each consensus position of LTR7. Results of all- and unique-read TFBSs are shown in the left and right panels, respectively. C) Number of TF-binding motifs in HERV-TFBSs mapped on each consensus position of LTR7. Results of all- and unique-read TFBSs are shown in the left and right panel, respectively. D) Left, phylogenetic tree of LTR7 copies as seen in Fig 3G. Middle and right, TFBSs on each LTR7 copy in all-read (middle) and unique-read (right) TFBSs. The order of LTR7 copies is the same to the left tree. (TIFF) [file pgen.1006883.s010.tiff]

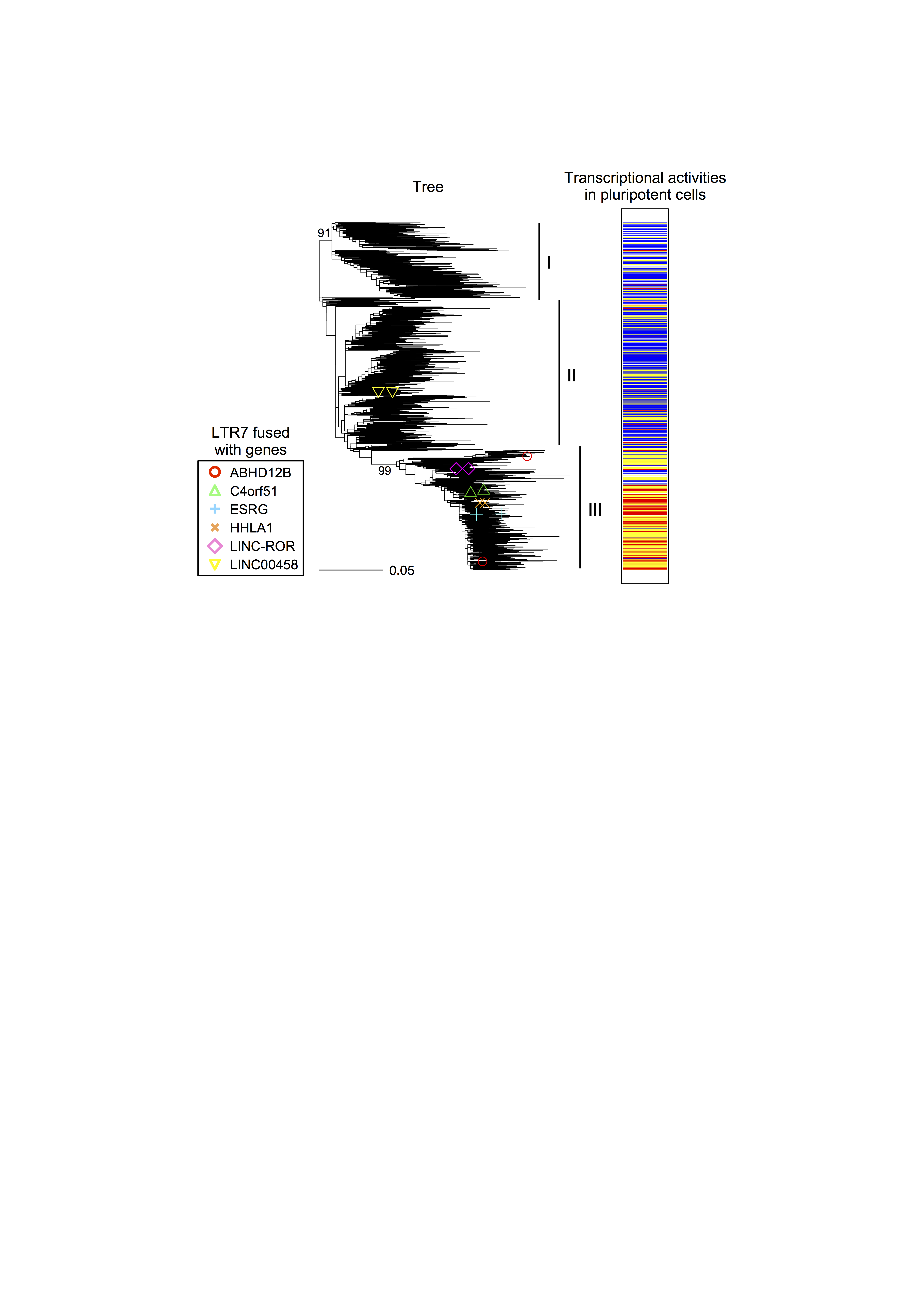

Supplement: S11 Fig — Left, the unrooted tree of LTR7 copies as seen in Fig 3G. LTR7 copies fused with ABHD12B, C4orf51, ESRG, HHLA1, LINC-ROR, and LINC00458 [10, 11, 39] are shown with markers. Right, transcriptional activities of LTR7 copies in pluripotent cells as defined by Wang et al. [11]. Red, highly active; yellow, moderately active; blue, inactive. (TIFF) [file pgen.1006883.s011.tiff]

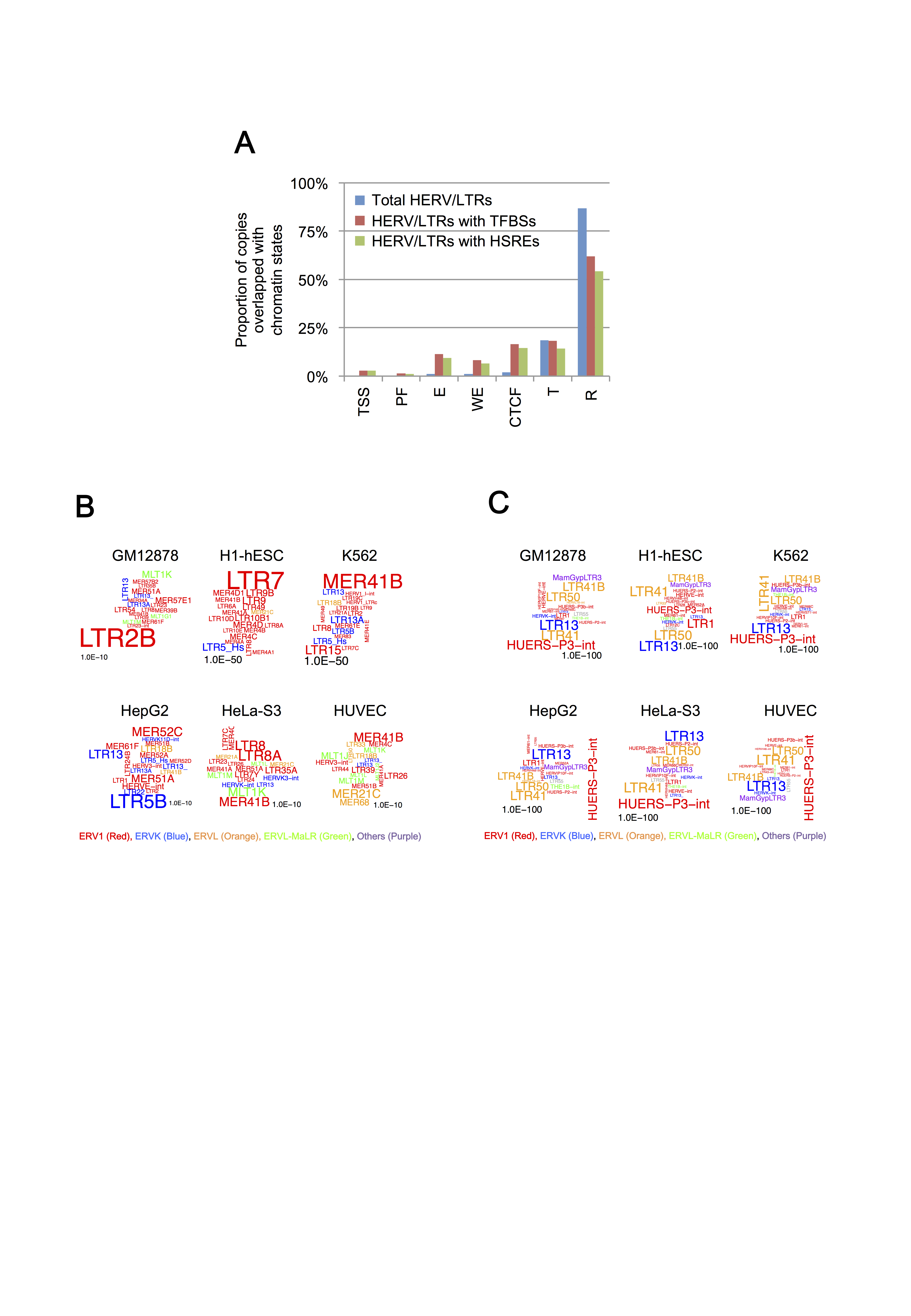

Supplement: S12 Fig — A) Proportion of HERV/LTR copies overlapped with each chromatin state. Chromatin states were predicted by genome segmentation method [47–49]. Proportions in total HERV/LTRs, HERV/LTRs with HERV-TFBSs, and HERV/LTRs with HSREs are separately shown. Results of unique-read TFBSs are shown. Averages of the proportions among six cells (GM12878, H1-hESC, K562, HepG2, HeLa-S3, and HUVEC) are shown. TSS, promoter region including TSS; PF, predicted promoter flanking region; E, enhancer; WE, weak enhancer or open chromatin cis regulatory element; CTCF, CTCF enriched element; T, transcribed region; R, repressed or low activity region. B) Word clouds showing HERV/LTRs enriched in enhancer regions of each cell type. The word sizes are proportional to −log10 (p values) calculated with Fisher’s exact test. The word colors indicate HERV/LTR families. Word clouds were created by wordcloud package implemented in R. C) Word clouds showing HERV/LTR types enriched in CTCF-binding regions of each cell type. (TIFF) [file pgen.1006883.s012.tiff]

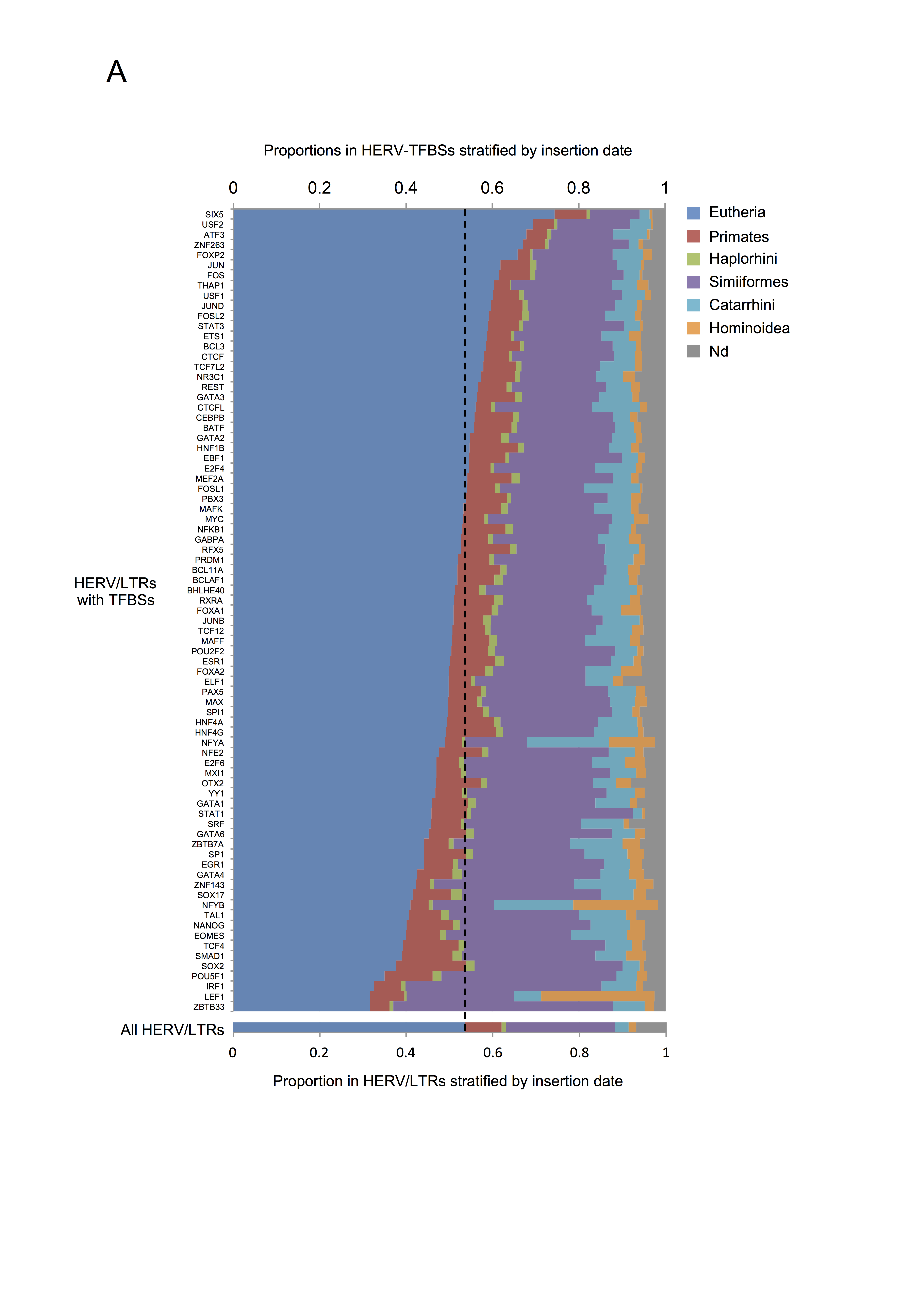

Supplement: S13 Fig — Results of unique-read TFBSs are shown. In respective TFs, HERV/LTRs with TFBSs were stratified by insertion date. TFs in which HERV-TFBSs overlapped with HERV/LTRs at least 1,000 times are shown. The integration date of HERV/LTR types was judged by distribution of orthologous of HERV/LTRs among the mammalian genome (see Materials and Methods). Proportions in all HERV/LTRs are shown at bottom of the figure. (TIFF) [file pgen.1006883.s013.tiff]

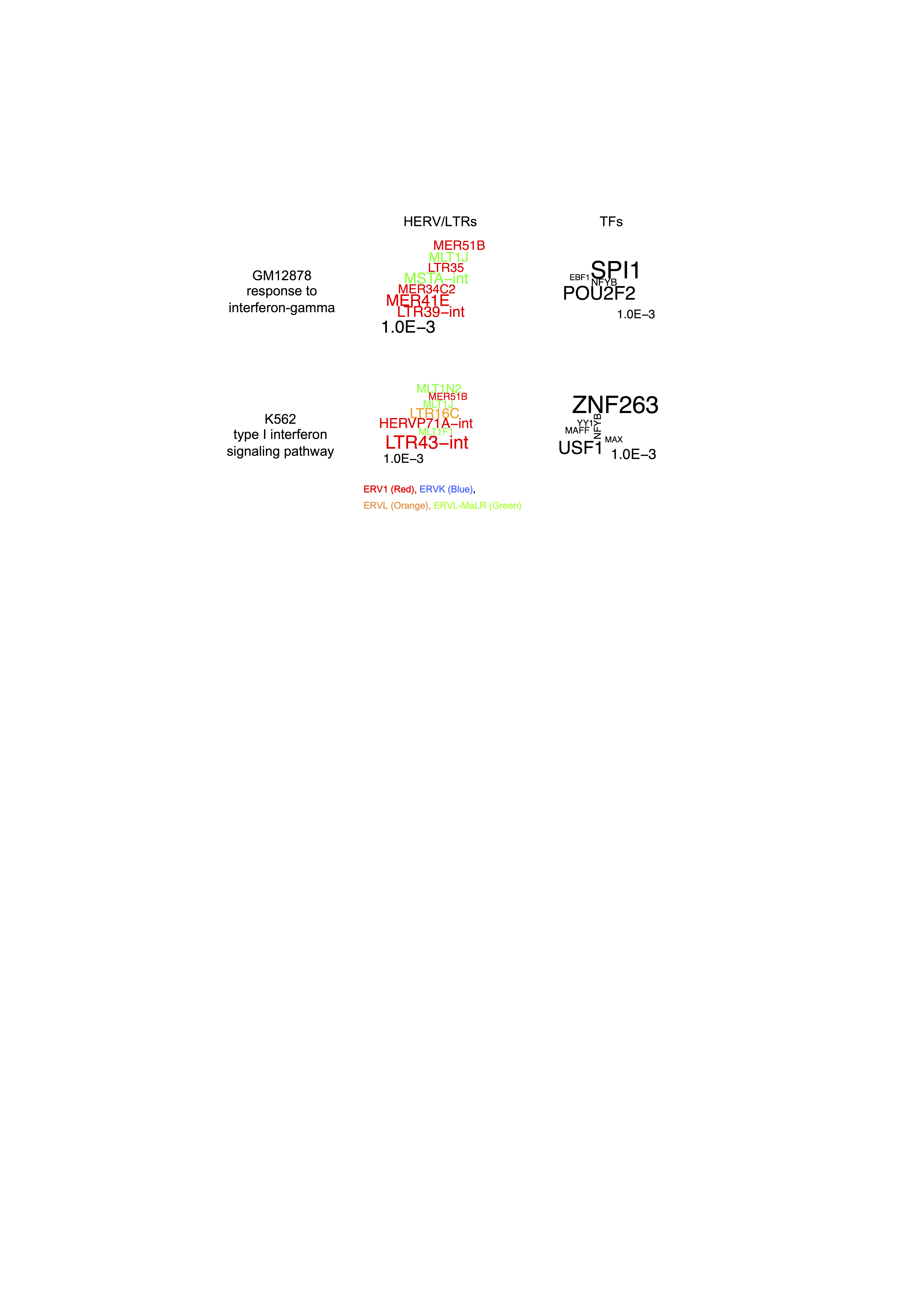

Supplement: S14 Fig — Regarding biological processes identified in Fig 5B, enrichment significance values of HERV/LTRs and TFs are shown. The word sizes are proportional to −log10 (p value) calculated with Fisher’s exact test. The word colors indicate HERV/LTR families. (TIFF) [file pgen.1006883.s014.tiff]

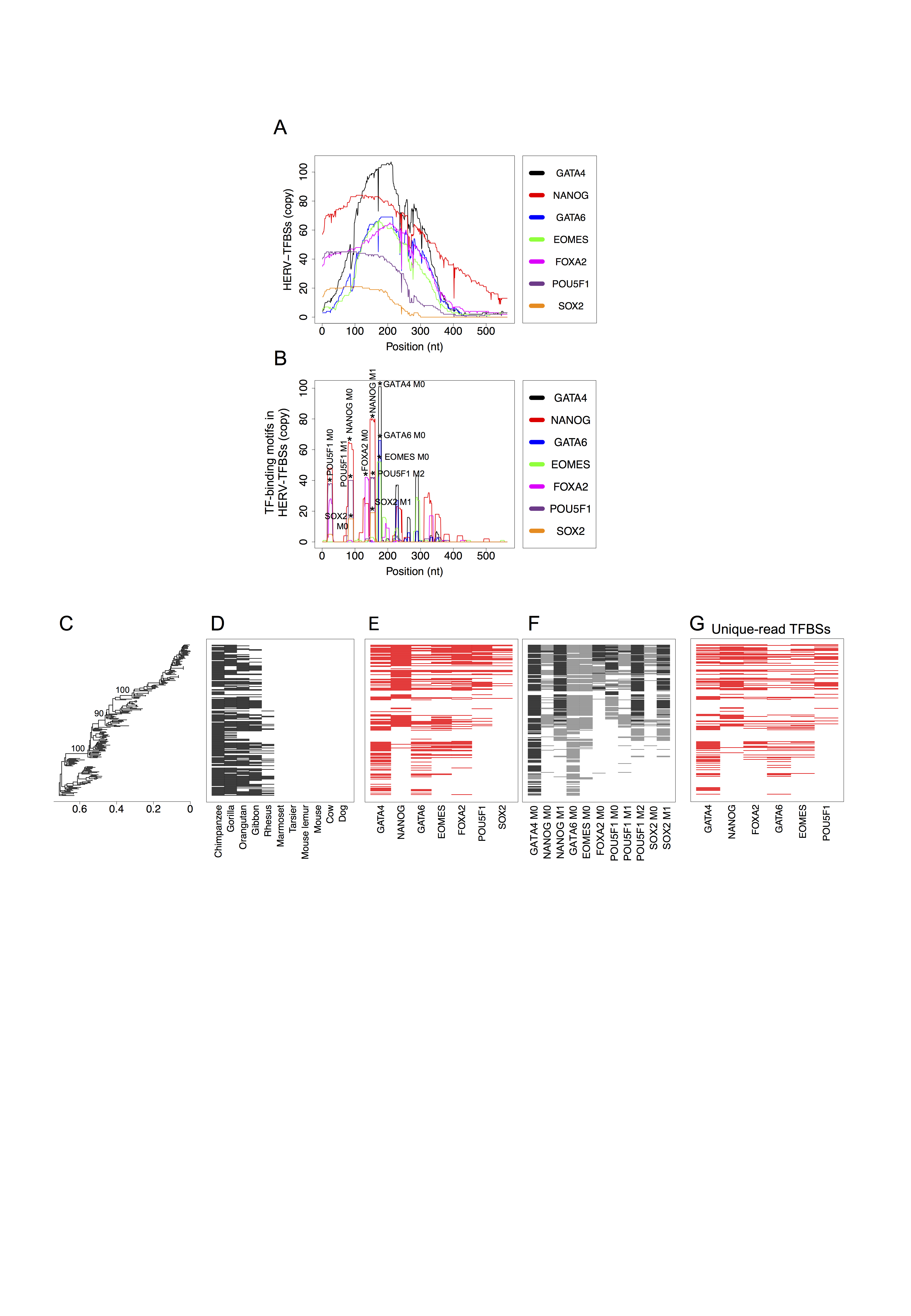

Supplement: S15 Fig — Results of all-read TFBSs are shown except for (G). A) Number of HERV-TFBSs mapped on each consensus position of LTR6A. The X-axis indicates nucleotide position of the consensus sequence. The Y-axis indicates number of HERV/LTR copies harboring HERV-TFBSs at each position. B) Number of TF-binding motifs in HERV-TFBSs mapped on each consensus position of LTR6A. The X-axis indicates nucleotide position of the consensus sequence. The Y-axis indicates number of HERV/LTR copies harboring the TF-binding motifs at each position. Peaks of the motifs corresponding to HSREs are indicated by an asterisk (*) with motif names. C) The unrooted phylogenetic tree of LTR6A copies constructed by maximum likelihood method. Fragmented and outlier copies were excluded from the analysis. In total, 204 (out of 288) of LTR6A copies were included in the tree. Representative supporting values calculated by SH-like test [68] are shown on the corresponding branches. D) Orthologous copies of LTR6A in the reference genomes of other mammals. E) TFBSs on each LTR6A copy. F) TF-binding motifs on each copy at positions corresponding to HSREs. Black and gray colors respectively indicate presence of motifs with p values of <0.0001 and <0.001. G) TFBSs on each LTR6A copy. Results of unique-read TFBSs are shown. (TIFF) [file pgen.1006883.s015.tiff]

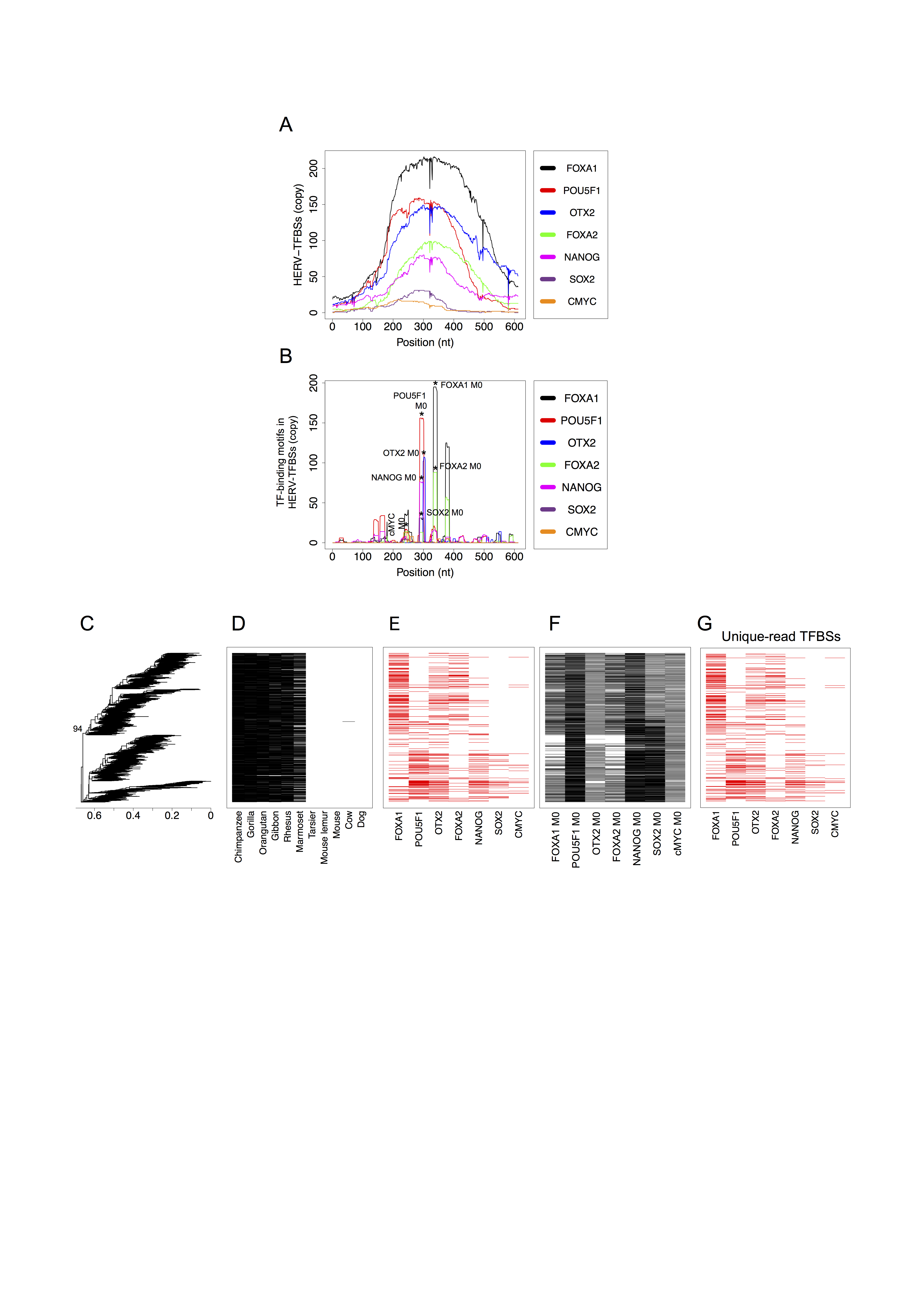

Supplement: S16 Fig — Results of all-read TFBSs are shown except for (G). A) Number of HERV-TFBSs mapped on each consensus position of LTR9. The X-axis indicates nucleotide position of the consensus sequence. The Y-axis indicates number of HERV/LTR copies harboring HERV-TFBSs at each position. B) Number of TF-binding motifs in HERV-TFBSs mapped on each consensus position of LTR9. The X-axis indicates nucleotide position of the consensus sequence. The Y-axis indicates number of HERV/LTR copies harboring the TF-binding motifs at each position. Peaks of the motifs corresponding to HSREs are indicated by an asterisk (*) with motif names. C) An unrooted phylogenetic tree of LTR9 copies constructed using the maximum likelihood method. Fragmented and outlier copies were excluded from the analysis. In total, 1,077 (out of 2,011) of LTR9 copies were included in the tree. Representative supporting values calculated by SH-like test [68] are shown on the corresponding branches. D) Orthologous copies of LTR9 in reference genomes of other mammals. E) TFBSs on each LTR9 copy. F) TF-binding motifs on each copy at positions corresponding to HSREs. The black and gray colors respectively indicate the presence of motifs with p values of <0.0001 and <0.001. G) TFBSs on each LTR9 copy. Results of unique-read TFBSs are shown. (TIFF) [file pgen.1006883.s016.tiff]

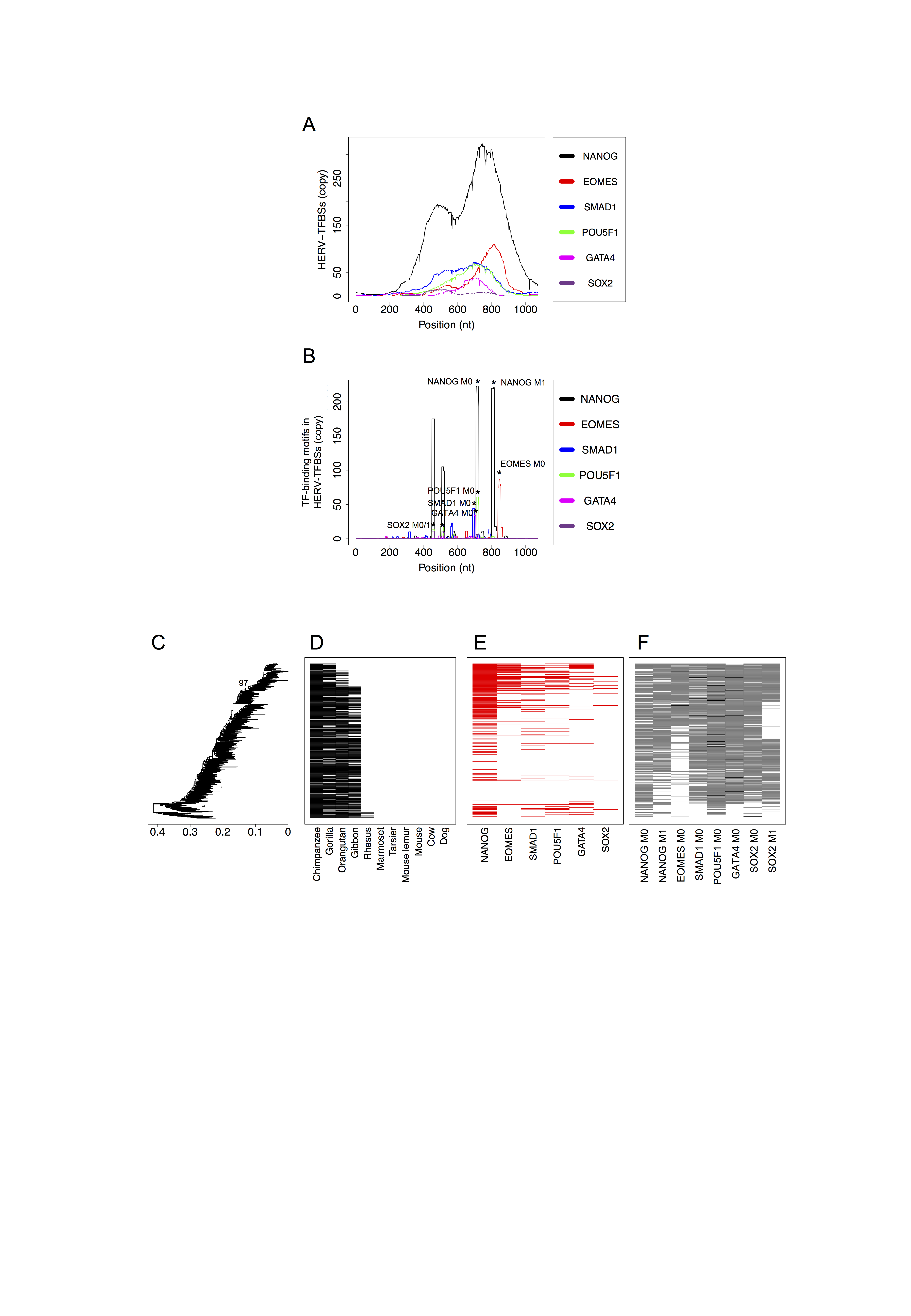

Supplement: S17 Fig — Results of all-read TFBSs are shown. A) Number of HERV-TFBSs mapped on each consensus position of MER11C. The X-axis indicates nucleotide position of the consensus sequence. The Y-axis indicates number of HERV/LTR copies harboring HERV-TFBSs at each position. B) Number of TF-binding motifs in HERV-TFBSs mapped on each consensus position of MER11C. The X-axis indicates nucleotide position of the consensus sequence. The Y-axis indicates number of HERV/LTR copies harboring the TF-binding motifs at each position. Peaks of the motifs corresponding to HSREs are indicated by an asterisk (*) with motif names. C) An unrooted phylogenetic tree of MER11C copies constructed using the maximum likelihood method. Fragmented and outlier copies were excluded from the analysis. In total, 748 (out of 866) of MER11C copies were included in the tree. Representative supporting values calculated by SH-like test [68] are shown on the corresponding branches. D) Orthologous copies of MER11C in reference genomes of other mammals. E) TFBSs on each MER11C copy. F) TF-binding motifs on each copy at positions corresponding to HSREs. The black and gray colors respectively indicate the presence of motifs with p values of <0.0001 and <0.001. (TIFF) [file pgen.1006883.s017.tiff]

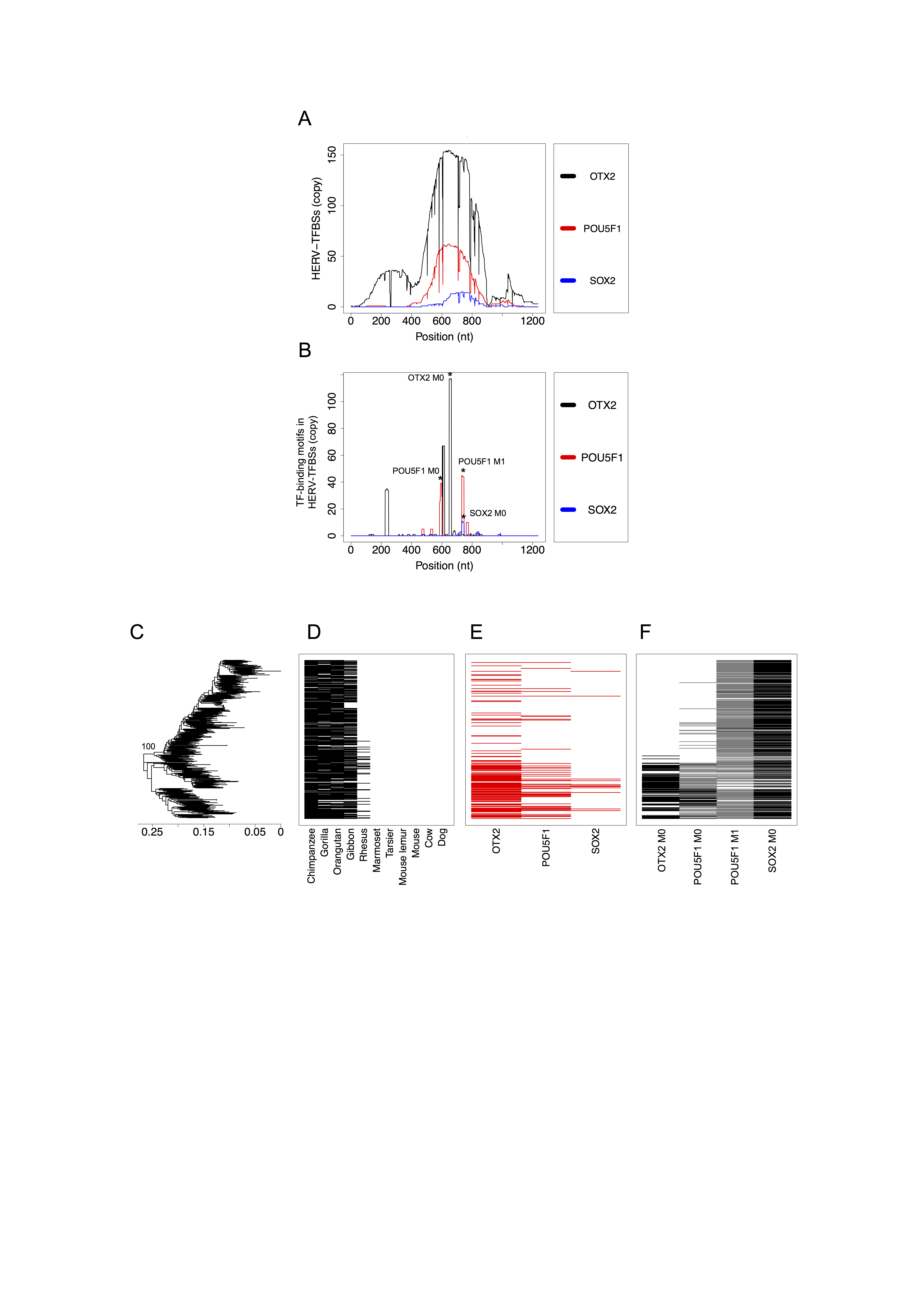

Supplement: S18 Fig — Results of all-read TFBSs are shown. A) Number of HERV-TFBSs mapped on each consensus position of MER11B. The X-axis indicates nucleotide position of the consensus sequence. The Y-axis indicates number of HERV/LTR copies harboring HERV-TFBSs at each position. B) Number of TF-binding motifs in HERV-TFBSs mapped on each consensus position of MER11B. The X-axis indicates nucleotide position of the consensus sequence. The Y-axis indicates number of HERV/LTR copies harboring the TF-binding motifs at each position. Peaks of the motifs corresponding to HSREs are indicated by an asterisk (*) with motif names. C) An unrooted phylogenetic tree of MER11B copies constructed using the maximum likelihood method. Fragmented and outlier copies were excluded from the analysis. In total, 377 (out of 548) of MER11B copies were included in the tree. Representative supporting values calculated by SH-like test [68] are shown on the corresponding branches. D) Orthologous copies of MER11B in reference genomes of other mammals. E) TFBSs on each MER11B copy. F) TF-binding motifs on each copy at positions corresponding to HSREs. The black and gray colors respectively indicate the presence of motifs with p values of <0.0001 and <0.001. (TIFF) [file pgen.1006883.s018.tiff]

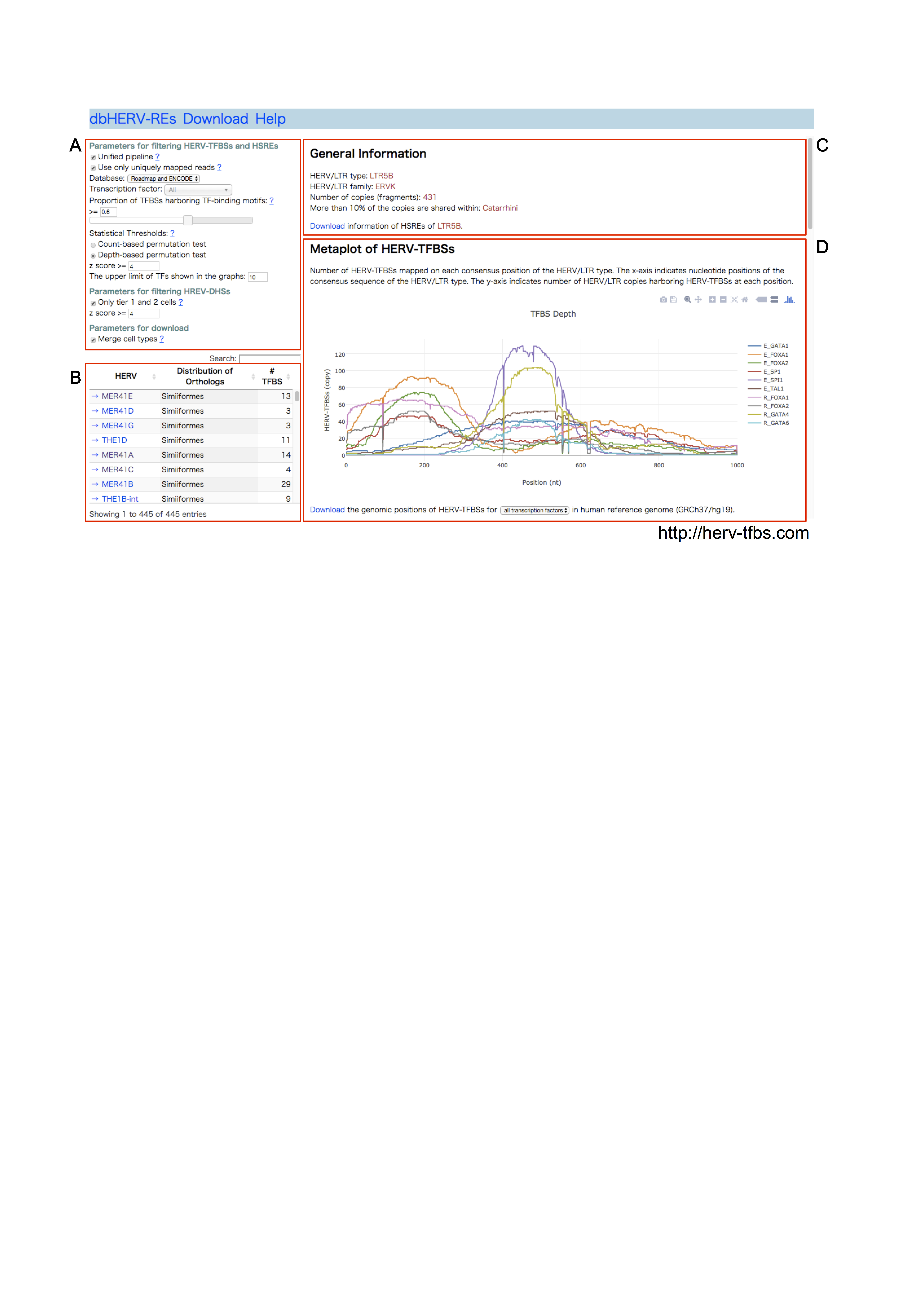

Supplement: S19 Fig — The screenshot when LTR5B was selected is shown. A) Statistical and other parameters filtering HERV-TFBSs, HSREs, and HERV-DHSs. B) The list of HERV/LTRs that can be selected under the parameters. C) General information of the selected HERV/LTRs. D) Visualized data. In this figure, the graph shows number of HERV-TFBSs mapped on each consensus position. (TIFF) [file pgen.1006883.s019.tiff]
