# Supplementary material for: Systematic identification and characterization of regulatory elements derived from human endogenous retroviruses
Source: PLoS Genet. 2017 Jul 12;13(7):e1006883. doi: 10.1371/journal.pgen.1006883 (PMC5529029; doi:10.1371/journal.pgen.1006883)
Supplement: S1 Table — Bold TFs were used for ChIP-Seq by ENCODE and Roadmap. (DOCX) [file pgen.1006883.s020.docx]

**S1 Table. TFs for which ChIP-Seq data was used in the present study.**

| **Dataset** | **TFs** |
| --- | --- |
| ENCODE | ATF3, BATF, BCL11A, BCL3, BCLAF1, BHLHE40, BRCA1, CEBPB, **CTCF**, CTCFL, E2F4, E2F6, EBF1, EGR1, ELF1, ELK4, ESR1, ETS1, FOS, FOSL1, FOSL2, **FOXA1**, **FOXA2**, FOXP2, GABPA, GATA1, GATA2, GATA3, **HNF4A**, HNF4G, IRF1, IRF3, JUN, JUNB, JUND, MAFF, MAFK, MAX, MEF2A, MEF2C, MXI1, **MYC**, **NANOG**, NFE2, NFKB1, NFYA, NFYB, NR2C2, NR3C1, NRF1, PAX5, PBX3, POU2F2, **POU5F1**, **PRDM1**, REST, RFX5, RXRA, SIX5, **SP1**, SP2, SPI1, SREBF1, SRF, STAT1, STAT2, STAT3, TAL1, TCF12, TCF7L2, THAP1, USF1, USF2, YY1, ZBTB33, ZBTB7A, ZNF143, ZNF263, ZNF274 |
| Roadmap | **CTCF**, EOMES, **FOXA1**, **FOXA2**, GATA4, GATA6, HAND1, HAND2, HEY1, HNF1B, **HNF4A**, KLF5, LEF1, **MYC**, **NANOG**, OTX2, PAX6, **POU5F1**, **PRDM1**, SMAD1, SMAD2/3, SMAD4, SNAI2, SOX17, SOX2, **SP1**, TCF4 |

Bold TFs were used for ChIP-Seq by ENCODE and Roadmap.
