# Supplementary material for: Systematic identification and characterization of regulatory elements derived from human endogenous retroviruses
Source: PLoS Genet. 2017 Jul 12;13(7):e1006883. doi: 10.1371/journal.pgen.1006883 (PMC5529029; doi:10.1371/journal.pgen.1006883)
Supplement: S2 Table — (DOCX) [file pgen.1006883.s021.docx]

**S2 Table. Sequencing and analytical pipelines of ChIP-Seq used in ENCODE and Roadmap.**

|  | **ENCODE (HAIB lab)** | **Roadmap (Tsankov *et al.* [41])** |
| --- | --- | --- |
| Read length | 25-50 bp | 25-36 bp |
| Layout | Single | Single/Paired |
| Platform | Genome Analyzer | HiSeq2000 |
| Mapping | Eland/Bowtie | MAQ/Bowtie2 |
| Filtering multiple mapped reads | Yes | No |
| Peak calling | SPP peak caller with calculation of IDR (Irreproducible Discovery Rate) | MACS |
| Using input control for peak calling | Yes | No |
