# Supplementary material for: Systematic identification and characterization of regulatory elements derived from human endogenous retroviruses
Source: PLoS Genet. 2017 Jul 12;13(7):e1006883. doi: 10.1371/journal.pgen.1006883 (PMC5529029; doi:10.1371/journal.pgen.1006883)
Supplement: S3 Table — Results of unique-read TFBSs are shown. Top 25 TFs with respect to proportions of HERV-TFBSs are shown. TFs in which TFBSs overlapped with HERV/LTRs at least 1,000 times are shown. (DOCX) [file pgen.1006883.s022.docx]

**S3 Table. Proportions of HERV-TFBSs in the entire TFBSs in respective TFs.**

| **TF** | **TFBSs** | **HERV-TFBSs** | **Proportion** |
| --- | --- | --- | --- |
| NFYB | 20,930 | 9,805 | 46.8% |
| NFYA | 8,786 | 1,999 | 22.8% |
| GATA6 | 32,230 | 7,073 | 21.9% |
| USF1 | 138,147 | 29,524 | 21.4% |
| GATA4 | 81,738 | 16,359 | 20.0% |
| TAL1 | 35,321 | 6,951 | 19.7% |
| SOX2 | 9,018 | 1,710 | 19.0% |
| SOX17 | 13,348 | 2,470 | 18.5% |
| USF2 | 33,600 | 6,205 | 18.5% |
| TCF4 | 12,764 | 2,209 | 17.3% |
| EOMES | 32,963 | 5,662 | 17.2% |
| STAT1 | 21,727 | 3,515 | 16.2% |
| YY1 | 193,183 | 30,506 | 15.8% |
| GATA1 | 49,133 | 7,714 | 15.7% |
| OTX2 | 126,138 | 19,576 | 15.5% |
| MAX | 268,057 | 41,571 | 15.5% |
| SPI1 | 131,487 | 19,731 | 15.0% |
| ZNF143 | 72,347 | 10,813 | 14.9% |
| GATA2 | 112,602 | 16,309 | 14.5% |
| SRF | 32,776 | 4,623 | 14.1% |
| NANOG | 102,008 | 13,903 | 13.6% |
| NFE2 | 56,155 | 7,528 | 13.4% |
| JUNB | 31,088 | 4,086 | 13.1% |
| JUND | 220,440 | 28,583 | 13.0% |
| STAT3 | 114,240 | 14,509 | 12.7% |

Top 25 TFs with respect to proportions of HERV-TFBSs are shown. TFs in which TFBSs overlapped with HERV/LTRs at least 1,000 times are shown.
