# Supplementary material for: Systematic identification and characterization of regulatory elements derived from human endogenous retroviruses
Source: PLoS Genet. 2017 Jul 12;13(7):e1006883. doi: 10.1371/journal.pgen.1006883 (PMC5529029; doi:10.1371/journal.pgen.1006883)
Supplement: S4 Table — Distance-based GO enrichment analysis using GREAT [53] algorithm was performed. Results of unique-read TFBSs are shown. TFBSs or HERV-TFBSs identified in cells treated with special conditions (e.g., supplement of interferon) were excluded. GO terms were summarized by REVIGO [73]. GO terms with hold enrichment scores >2 are shown. (DOCX) [file pgen.1006883.s023.docx]

**S4 Table. Distance-based GO enrichment analysis to ascertain biological processes in which HERV-TFBSs were more enriched compared to the other TFBSs.**

| **Cell** | **GO term (biological process)** | **P value (-log10)** | **Fold enrichment** |
| --- | --- | --- | --- |
| GM12878 | negative regulation of viral process | 62.4 | 4.5 |
|  | cytidine deamination | 51.5 | 3.5 |
|  | positive regulation of type 2 immune response | 43.6 | 2.6 |
|  | glutamate receptor signaling pathway | 42.9 | 2.1 |
|  | DNA cytosine deamination | 42.2 | 3.9 |
| H1-hESC | heparan sulfate proteoglycan metabolic process | 32.3 | 2.2 |
|  | negative regulation of viral process | 27.9 | 3.1 |
|  | serotonin receptor signaling pathway | 27.9 | 2.0 |
|  | presynaptic membrane assembly | 24.4 | 2.0 |
|  | positive regulation of fever generation | 23.8 | 2.7 |
| K562 | cholesterol catabolic process | 100.5 | 2.4 |
|  | cellular response to estrogen stimulus | 73.2 | 2.1 |
|  | heparan sulfate proteoglycan metabolic process | 51.7 | 2.3 |
|  | negative regulation of triglyceride catabolic process | 41.7 | 2.1 |
|  | positive regulation of type 2 immune response | 41.2 | 2.5 |
| HepG2 | flavonoid biosynthetic process | 61.4 | 2.9 |
|  | cellular glucuronidation | 46.7 | 2.3 |
|  | thyroid hormone metabolic process | 39.7 | 2.3 |
|  | doxorubicin metabolic process | 39.5 | 2.3 |
|  | cellular response to prostaglandin D stimulus | 39.2 | 3.3 |
| HeLa-S3 | cholesterol catabolic process | 44.9 | 2.8 |
|  | positive regulation of chemokine secretion | 22.9 | 2.6 |
|  | stabilization of membrane potential | 20.5 | 2.1 |
|  | androgen biosynthetic process | 19.0 | 2.1 |
|  | opioid receptor signaling pathway | 18.2 | 3.0 |
| HUVEC | flavonoid biosynthetic process | 8.2 | 3.6 |
|  | neuroligin clustering involved in postsynaptic membrane assembly | 8.1 | 2.1 |
|  | oligosaccharide biosynthetic process | 7.8 | 2.0 |
|  | cholesterol catabolic process | 7.1 | 2.3 |
|  | protection from natural killer cell mediated cytotoxicity | 7.0 | 3.4 |

Distance-based GO enrichment analysis using GREAT [53] algorithm was performed. Results of unique-read TFBSs are shown. TFBSs or HERV-TFBSs identified in cells treated with special conditions (e.g., supplement of interferon) were excluded. GO terms were summarized by REVIGO [73]. GO terms with hold enrichment scores >2 are shown.
