# Supplementary material for: Systematic identification and characterization of regulatory elements derived from human endogenous retroviruses
Source: PLoS Genet. 2017 Jul 12;13(7):e1006883. doi: 10.1371/journal.pgen.1006883 (PMC5529029; doi:10.1371/journal.pgen.1006883)
Supplement: S5 Table — Distance-based GO enrichment analysis using GREAT algorithm [53] was performed. Results of unique-read TFBSs are shown. HERV-TFBSs identified in cells treated with special conditions (e.g., supplement of interferon) were excluded. GO terms were summarized by REVIGO [73]. GO terms with hold enrichment scores >2 are shown. (DOCX) [file pgen.1006883.s024.docx]

**S5 Table. Distance-based GO enrichment analysis to ascertain biological processes in which HERV/LTRs harboring TFBSs were more enriched compared to entire HERV/LTRs.**

| **Cell** | **GO term (biological process)** | **P value (-log10)** | **Fold enrichment** |
| --- | --- | --- | --- |
| GM12878 | type I interferon signaling pathway | 55.0 | 2.6 |
|  | liver regeneration | 28.9 | 2.5 |
|  | blood vessel endothelial cell migration | 18.1 | 2.7 |
|  | negative regulation of viral genome replication | 16.7 | 2.1 |
|  | entrainment of circadian clock by photoperiod | 16.5 | 2.3 |
| H1-hESC | response to purine-containing compound | 8.4 | 2.1 |
|  | positive regulation of keratinocyte differentiation | 8.2 | 2.2 |
|  | positive regulation of meiotic nuclear division | 8.1 | 2.0 |
|  | negative regulation of protein dephosphorylation | 6.4 | 2.2 |
|  | high-density lipoprotein particle clearance | 6.3 | 2.7 |
| K562 | platelet aggregation | 37.5 | 2.0 |
|  | hepatocyte apoptotic process | 31.6 | 2.5 |
|  | liver regeneration | 30.9 | 2.1 |
|  | cholesterol catabolic process | 26.3 | 2.5 |
|  | regulation of cytokine production | 26.1 | 2.2 |
| HepG2 | cellular response to estrogen stimulus | 15.2 | 2.0 |
|  | platelet-derived growth factor receptor-beta signaling pathway | 13.8 | 2.8 |
|  | cholesterol catabolic process | 13.4 | 2.1 |
|  | L-serine transport | 12.7 | 2.3 |
|  | cellular response to nutrient levels | 10.9 | 2.3 |
| HeLa-S3 | cholesterol catabolic process | 17.7 | 2.9 |
|  | embryonic placenta development | 16.0 | 2.3 |
|  | liver regeneration | 15.4 | 2.1 |
|  | regulation of interferon-gamma-mediated signaling pathway | 14.5 | 2.3 |
|  | positive regulation of transcription from RNA polymerase II promoter in response to endoplasmic reticulum stress | 13.9 | 3.5 |
| HUVEC | programmed necrotic cell death | 11.1 | 3.0 |
|  | positive regulation of nitric-oxide synthase activity | 8.1 | 2.2 |
|  | protection from natural killer cell mediated cytotoxicity | 7.2 | 3.9 |
|  | high-density lipoprotein particle clearance | 6.7 | 4.3 |
|  | androgen biosynthetic process | 6.6 | 2.6 |

Distance-based GO enrichment analysis using GREAT algorithm [53] was performed. Results of unique-read TFBSs are shown. HERV-TFBSs identified in cells treated with special conditions (e.g., supplement of interferon) were excluded. GO terms were summarized by REVIGO [73]. GO terms with hold enrichment scores >2 are shown.
