# Supplementary material for: Systematic identification and characterization of regulatory elements derived from human endogenous retroviruses
Source: PLoS Genet. 2017 Jul 12;13(7):e1006883. doi: 10.1371/journal.pgen.1006883 (PMC5529029; doi:10.1371/journal.pgen.1006883)
Supplement: S6 Table — Results in unique-read TFBSs are shown. The GREAT enrichment analyses [53] were performed using sets of HERV-TFBSs harboring each type of HSRE in respective cell types, and then GO terms associated with many kinds of HSREs (>10) were summarized separately in cell types (up to 15 in each cell type). (DOCX) [file pgen.1006883.s025.docx]

**S6 Table. Biological processes in which many types of HSREs were enriched.**

| **Cell** | **GO term (biological process)** | **# of HSRE types associated with the GO term** |
| --- | --- | --- |
| GM12878 | negative regulation of transcription from RNA polymerase II promoter | 17 |
|  | interferon-gamma-mediated signaling pathway | 16 |
|  | transcription, DNA-templated | 14 |
|  | small molecule metabolic process | 14 |
|  | blood coagulation | 14 |
|  | response to estradiol | 13 |
|  | regulation of transcription, DNA-templated | 13 |
|  | protein phosphorylation | 12 |
|  | liver regeneration | 12 |
|  | inflammatory response | 12 |
|  | transforming growth factor beta receptor signaling pathway | 11 |
|  | positive regulation of defense response to virus by host | 11 |
| H1-hESC | transcription, DNA-templated | 16 |
|  | small molecule metabolic process | 14 |
|  | response to wounding | 14 |
|  | liver regeneration | 14 |
|  | defense response to bacterium | 13 |
|  | positive regulation of canonical Wnt signaling pathway | 12 |
|  | positive regulation of Wnt signaling pathway | 12 |
|  | negative regulation of transcription, DNA-templated | 12 |
|  | negative regulation of transcription from RNA polymerase II promoter | 12 |
|  | response to endoplasmic reticulum stress | 11 |
|  | positive regulation of transcription, DNA-templated | 11 |
|  | negative regulation of cell proliferation | 11 |
|  | negative regulation of NF-kappaB transcription factor activity | 11 |
| K562 | small molecule metabolic process | 79 |
|  | transcription, DNA-templated | 52 |
|  | protein phosphorylation | 49 |
|  | immune response | 41 |
|  | blood coagulation | 39 |
|  | innate immune response | 38 |
|  | negative regulation of transcription from RNA polymerase II promoter | 37 |
|  | oxidation-reduction process | 32 |
|  | negative regulation of transcription, DNA-templated | 31 |
|  | inflammatory response | 31 |
|  | viral process | 30 |
|  | cellular lipid metabolic process | 30 |
|  | xenobiotic metabolic process | 29 |
|  | transforming growth factor beta receptor signaling pathway | 29 |
|  | regulation of transcription, DNA-templated | 29 |
|  | gene expression | 29 |
| HepG2 | small molecule metabolic process | 23 |
|  | xenobiotic metabolic process | 20 |
|  | defense response to bacterium | 18 |
|  | viral process | 17 |
|  | transcription, DNA-templated | 14 |
|  | response to wounding | 14 |
|  | negative regulation of apoptotic process | 14 |
|  | kidney development | 14 |
|  | cell differentiation | 14 |
|  | blood coagulation | 14 |
|  | angiogenesis | 14 |
|  | ubiquitin-dependent protein catabolic process | 13 |
|  | positive regulation of transcription from RNA polymerase II promoter | 13 |
|  | chondroitin sulfate metabolic process | 13 |
|  | response to ethanol | 12 |
| HeLa-S3 | small molecule metabolic process | 14 |
|  | immune response | 14 |
|  | liver regeneration | 11 |
|  | interferon-gamma-mediated signaling pathway | 11 |
|  | cytokine-mediated signaling pathway | 11 |
|  | cellular response to lipopolysaccharide | 11 |
|  | cellular nitrogen compound metabolic process | 11 |

Results in unique-read TFBSs are shown. The GREAT enrichment analyses [53] were performed using sets of HERV-TFBSs harboring each type of HSRE in respective cell types, and then GO terms associated with many kinds of HSREs (>10) were summarized separately in cell types (up to 15 in each cell type).
