# Supplementary material for: Systematic identification and characterization of regulatory elements derived from human endogenous retroviruses
Source: PLoS Genet. 2017 Jul 12;13(7):e1006883. doi: 10.1371/journal.pgen.1006883 (PMC5529029; doi:10.1371/journal.pgen.1006883)
Supplement: S7 Table — Hi-C-based GO enrichment analysis with GREAT [53] algorithm was performed. Results of unique-read TFBSs are shown. TFBSs or HERV-TFBSs identified in cells treated with special conditions (e.g., supplement of interferon) were excluded. GO terms were summarized by REVIGO [73]. GO terms with hold enrichment scores >2 are shown. (DOCX) [file pgen.1006883.s026.docx]

**S7 Table. Hi-C-based GO enrichment analysis to ascertain biological processes in which HERV-TFBSs were more enriched than the other TFBSs.**

| **GO term (biological process)** | **P value (-log10)** | | **Fold enrichment** |
| --- | --- | --- | --- |
| N-glycan processing | | 50.0 | 4.3 |
| intraciliary transport | | 48.6 | 5.3 |
| positive regulation of interleukin-2 production | | 35.4 | 3.6 |
| dendritic cell chemotaxis | | 33.6 | 3.0 |
| positive regulation of alpha-beta T cell proliferation | | 32.6 | 3.7 |
| positive regulation of keratinocyte differentiation | | 32.4 | 2.7 |
| positive regulation of synapse maturation | | 28.4 | 3.5 |
| detection of chemical stimulus involved in sensory perception of smell | | 28.4 | 2.1 |
| phosphate ion transmembrane transport | | 27.1 | 2.6 |
| cellular response to exogenous dsRNA | | 25.7 | 3.0 |
| regulation of cysteine-type endopeptidase activity involved in apoptotic process | | 25.7 | 3.5 |
| positive regulation of dendrite morphogenesis | | 24.3 | 2.5 |
| regulation of phagocytosis | | 23.8 | 3.4 |
| positive regulation of protein oligomerization | | 23.7 | 3.5 |
| response to tumor necrosis factor | | 23.1 | 2.1 |

Hi-C-based GO enrichment analysis with GREAT [53] algorithm was performed. Results of unique-read TFBSs are shown. TFBSs or HERV-TFBSs identified in cells treated with special conditions (e.g., supplement of interferon) were excluded. GO terms were summarized by REVIGO [73]. GO terms with hold enrichment scores >2 are shown.
